# Supplementary material for: Asymmetric one-pot transformation of isoflavones to pterocarpans and its application in phytoalexin synthesis
Source: Nat Commun. 2020 Jun 18;11:3091. doi: 10.1038/s41467-020-16933-y (PMC7303153; doi:10.1038/s41467-020-16933-y)
Supplement: Supplementary file 1 — Supplementary Information [file 41467_2020_16933_MOESM1_ESM.pdf]

Supplementary Information

Asymmetric one-pot transformation of isoflavones to pterocarpans and its application in phytoalexin synthesis

Ciesielski et al.

Supplementary Note 1

General Information

DMF and MeCN were dried over 4 Å molecular sieves. Dry DCM and THF were obtained from a solvent purification system MB-SPS-800. Et_3N was freshly distilled over CaH_2. All other commercially available reagents were used as received without further purification. All reactions were performed under argon atmosphere if not stated otherwise. TLC was performed on Merck silica gel 60 F_{254} 0.2 mm precoated aluminium plates. Product spots were visualised by UV light (254 nm) and subsequently developed using anisaldehyde solution or permanganate solution as appropriate. Flash column chromatography was carried out using silica gel (Merck, particle size 40-63 microns). Melting points were measured on a Wagner & Munz PolyTherm A and are uncorrected. Infrared spectra were recorded on a Thermonicolet Avatar 360 instrument using ATR. NMR spectra were recorded on a Bruker AC-300 P (^{1}H: 300 MHz, ^{13}C: 75 MHz), on a Bruker DRX 500 P (^{1}H: 500 MHz, ^{13}C: 126 MHz) or a Bruker AC-600 P (^{1}H: 600 MHz, ^{13}C: 151 MHz) spectrometer. Chemical shifts (δ) are quoted in parts per million (ppm) downfield of tetramethylsilane, using residual proton-containing solvent as internal standard ($CDCl_3$ at 7.27 ppm (^{1}H) and 77.00 ppm (^{13}C) or MeOD-d_4 at 3.31 ppm (^{1}H) and 49.15 ppm (^{13}C) or acetone-d_6 at 2.05 ppm (^{1}H) and 29.92 ppm (^{13}C) or DMSO-d_4 at 2.50 ppm (^{1}H) and 39.51 ppm (^{13}C). Abbreviations used in the description of resonances are: s (singlet), d (doublet), t (triplet), q (quartet), br (broad), m (multiplet). Coupling constants (*J*) are quoted to the nearest 0.1 Hz. Mass spectra were recorded with an Agilent 5973N detector coupled with an Agilent 6890N GC (GC-MS, 70 eV) or else with a Bruker Esquire-LC (direct injection as a methanolic NH_4OAc solution, ESI). HRMS spectra were recorded on an Agilent 6500 Series Q-TOF (ESI-TOF). Optical rotations were measured on a Perkin Elmer 341 LC polarimeter. Elemental analysis was performed on a Hekatech EA 3000. Enantiomeric excesses were determined by chiral HPLC on an Agilent 1100 Series with photodiode array detector (DAD) using

the chiral columns Chiralpak® AD (DAICEL, 250 mm, inner diameter 4.6 mm, particle size 10 µm), Lux® Cellulose-1 (Phenomenex, 250 mm, inner diameter 4.6 mm, particle size 5 µm) and Lux® Amylose-1 (Phenomenex, 250 mm, inner diameter 4.6 mm, particle size 5 µm). All measurements were carried out at ambient temperature. Unless stated otherwise, racemic samples were prepared according to the procedures described for the enantiomerically pure series using a racemic mixture of the chiral ligand in asymmetric transfer hydrogenation reaction.

Supplementary Note 2

First-Generation Synthesis: Preparation of Isoflavone 17

Improved Synthesis of 7-(Benzyloxy)-3-iodo-4*H*-chromen-4-one (15)

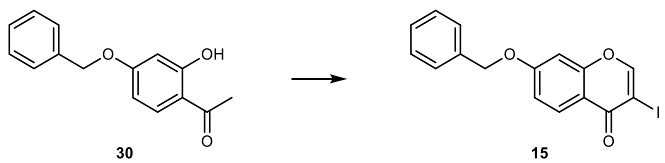

Acetophenone **30**[1] (6.079 g, 25.1 mmol) and *N*,*N*-dimethylformamide dimethyl acetal (13.3 mL, 100 mmol, 4.0 equiv.) were stirred in a 50 mL round-bottomed flask with distilling head at 95 °C for 6 h. Afterwards, all volatile components were distilled off in vacuo at 60 °C. The resulting red- to yellow-coloured solid was dissolved in $CHCl_3$ (50 mL), and pyridine (3.1 mL, 37.6 mmol, 1.5 equiv.) and iodine (12.74 g, 50.2 mmol, 2.0 equiv.) were added successively. The reaction mixture was stirred at room temperature for 15.5 h. Saturated aqueous sodium thiosulfate (30 mL) was added, and the mixture was stirred for 1 h. The aqueous layer was extracted four times with DCM, the combined organic layers were dried over $MgSO_4$, and the solvents were removed in vacuo. The residue was dissolved in EtOAc/isohexane 1:1 (20 mL), and the solvents were removed again in vacuo. The resulting solid was suspended in a small amount of EtOAc, filtered off, and washed twice with isohexane to yield iodochromone **15** (6.36 g, 67%). The solvents of the combined organic layers were removed in vacuo, and the residue was purified by flash chromatography (isohexane:DCM:EtOAc 8:8:1 *v/v*) to afford **15** (2.94 g, 31%) as a white to yellowish solid (combined yield 98%).

mp: 124–126 °C (lit.[1] 126–128 °C); TLC (isohexane:DCM:EtOAc 8:8:1 *v/v*): R_f = 0.35; ^{1}H NMR (500 MHz, $CDCl_3$): δ 8.21 (s, 1H), 8.16 (d, *J* = 8.8 Hz, 1H), 7.35–7.49 (m, 5H), 7.08 (dd, *J* = 9.0 Hz, *J* = 2.4 Hz, 1H), 6.92 (d, *J* = 2.2 Hz, 1H), 5.17 (s, 2H) ppm; ^{13}C NMR (125 MHz, $CDCl_3$): δ 172.56 (C_q), 163.30 (C_q), 157.80 (C_q), 157.19 (CH), 135.40 (C_q), 128.78 (CH), 128.49 (CH), 128.14 (CH), 127.52 (CH), 115.83 (C_q), 115.76 (CH), 101.11 (CH), 87.14 (C_q),

70.62 (CH_2) ppm; IR (ATR): 3067, 1621, 1435, 1268, 1064, 825, 733 cm^{-1}; GC-MS (EI): m/z = 378 $[M]^+$.

Synthesis of Iodobenzene 32

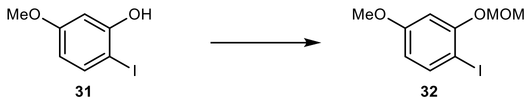

Iodophenol **31**[2] (1.653 g, 6.61 mmol) was dissolved in DCM (6.6 mL) and *N*,*N*-diisopropylethylamine (1.7 mL, 9.92 mmol, 1.5 equiv.) in a 10 mL round-bottomed flask and cooled to 0 °C. Methoxymethyl chloride (0.65 mL, 8.60 mmol, 1.3 equiv.) was added, and the reaction mixture was stirred at room temperature for 15 minutes. Saturated aqueous NH_4Cl solution was added. The aqueous layer was extracted three times with DCM, the combined organic layers were dried over $MgSO_4$, and the solvents were removed in vacuo. The residue was purified by flash chromatography (isohexane:EtOAc 4:1 *v/v*) to afford iodobenzene **32** (1.846 g, 95%) as a colourless liquid.

TLC (isohexane:EtOAc 4:1 *v/v*): R_f = 0.42; ^{1}H NMR (300 MHz, $CDCl_3$): δ 7.63 (d, *J* = 8.7 Hz, 1H), 6.70 (d, *J* = 2.6 Hz, 1H), 6.39 (dd, *J* = 8.7 Hz, *J* = 2.8 Hz, 1H), 5.23 (s, 2H), 3.79 (s, 3H), 3.52 (s, 3H) ppm; ^{13}C NMR (75 MHz, $CDCl_3$): δ 161.18 (C_q), 156.82 (C_q), 139.13 (CH), 109.06 (CH), 102.36 (CH), 95.01 (CH_2), 75.79 (C_q), 56.40 (CH_3), 55.51 (CH_3) ppm; IR (ATR): 2956, 1472, 1151, 982, 830 cm^{-1}; GC-MS (EI): m/z = 294 $[M]^+$; analysis (calcd., found for $C_9H_{11}IO_3$): C (36.76, 37.08), H (3.77, 3.84).

Suzuki Coupling of Iodide 15 and the Boronic Acid 16

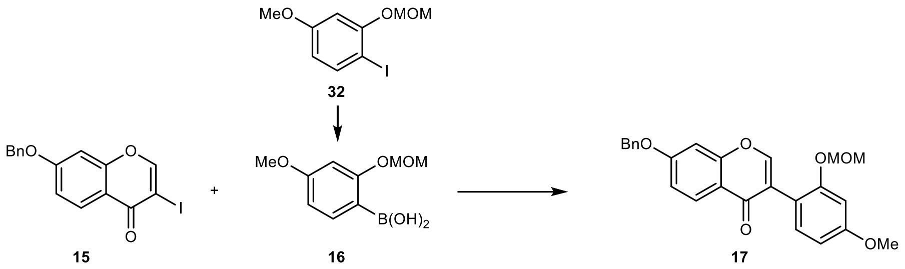

Iodobenzene **32** (1.833 g, 6.23 mmol, 2.0 equiv.) was dissolved in THF (14 mL) in a 50 mL round-bottomed flask and cooled to –78 °C. *n*-Butyllithium solution (5.1 mL, 1.6 M in hexanes, 8.10 mmol, 2.6 equiv.) was added over 15 min, and the reaction mixture was stirred for 5 min at –78 °C. Triisopropyl borate (2.9 mL, 12.5 mmol, 4.0 equiv.) was added, and the reaction mixture was stirred for 20 min at –78 °C. The dry-ice/acetone bath was removed, and the reaction mixture was stirred for 3.7 h and then poured onto 1 N hydrochloric acid. The aqueous layer was extracted three times with EtOAc, the combined organic layers were washed twice with brine and dried over $MgSO_4$, and the solvents were removed in vacuo. To the crude boronic acid was added iodide **15** (1.192 g, 3.15 mmol, 1.0 equiv.), potassium carbonate (1.307 g, 9.46 mmol, 3.0 equiv.), BHT (6.9 mg, 31.5 µmol, 1 mol%), 1,4-dioxane (13 mL), and water (5.6 mL). The mixture was purged with argon for 10 min. Tricyclohexylphosphine (70.7 mg, 0.25 mmol, 8 mol%) and $Pd_2(dba)_3$ (115.5 mg, 0.13 mmol, 4 mol%) were added successively, and the reaction mixture was stirred at 50 °C for 20 min. The reaction mixture was cooled to room temperature, and saturated aqueous NH_4Cl solution was added. The aqueous layer was extracted three times with EtOAc, the combined organic layers were dried over $MgSO_4$, and the solvents were removed in vacuo. The residue was purified by repeated flash chromatography (1st: DCM:EtOAc 8:1; 2nd: isohexane:EtOAc 1:1 *v/v*) to afford isoflavone **17** (1.104 g, 84%) as an off-white solid.

mp: 112–113 °C; TLC (isohexane:EtOAc 2:1 *v/v*): R_f = 0.30; ^{1}H NMR (300 MHz, $CDCl_3$): δ 8.21 (d, *J* = 9.1 Hz, 1H), 7.88 (s, 1H), 7.33–7.50 (m, 5H), 7.24 (d, *J* = 8.3 Hz, 1H), 7.07 (dd, *J*

= 9.0 Hz, *J* = 2.4 Hz, 1H), 6.94 (d, *J* = 2.5 Hz, 1H), 6.82 (d, *J* = 2.5 Hz, 1H), 6.63 (dd, *J* = 8.5 Hz, *J* = 2.5 Hz, 1H), 5.19 (s, 2H), 5.13 (s, 2H), 3.84 (s, 3H), 3.43 (s, 3H) ppm; ^{13}C NMR (75 MHz, $CDCl_3$): δ 175.59 (C_q), 162.85 (C_q), 160.96 (C_q), 157.88 (C_q), 156.42 (C_q), 153.54 (CH), 135.78 (C_q), 132.11 (CH), 128.76 (CH), 128.38 (CH), 127.86 (CH), 127.49 (CH), 122.49 (C_q), 118.64 (C_q), 114.80 (CH), 114.29 (C_q), 106.66 (CH), 102.22 (CH), 101.31 (CH), 95.10 (CH_2), 70.50 (CH_2), 56.11 (CH_3), 55.45 (CH_3) ppm; IR (ATR): 2955, 1606, 1256, 1006, 696 cm^{-1}; GC-MS (EI): m/z = 418 $[M]^+$; analysis (calcd., found for $C_{25}H_{22}O_6$): C (71.76, 71.56), H (5.30, 5.68).

Supplementary Note 3

First-Generation Synthesis: Preparation of Isoflavanone 18

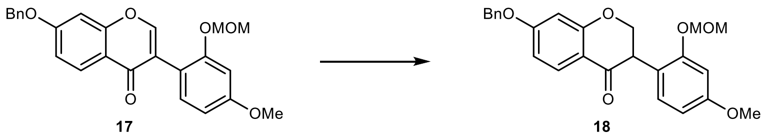

Isoflavone **17** (417.6 mg, 1.00 mmol, 1.0 equiv.) was dissolved in THF (8.0 mL) in a 25 mL round-bottomed flask and cooled to –78 °C. L-selectride (1.2 mL, 1 M in THF, 1.20 mmol, 1.2 equiv.) was added, and the reaction mixture was stirred at –78 °C for 65 min. The reaction was quenched by the addition of methanol (2 mL), warmed to room temperature, and saturated aqueous NH_4Cl solution was added. The aqueous layer was extracted three times with EtOAc, the combined organic layers were dried over $MgSO_4$, and the solvents were removed in vacuo. The residue was purified by flash chromatography (isohexane:EtOAc 2:1 *v/v*) to afford isoflavanone **18** (393.7 mg, ≈94%) as a white solid.

mp: 27–53 °C; TLC (isohexane:EtOAc 2:1 *v/v*): R_f = 0.46; ^{1}H NMR (300 MHz, $CDCl_3$): δ 7.94 (d, *J* = 8.9 Hz, 1H), 7.32–7.48 (m, 5H), 7.03 (d, *J* = 8.5 Hz, 1H), 6.75 (d, *J* = 2.5 Hz, 1H), 6.70 (dd, *J* = 8.8 Hz, *J* = 2.4 Hz, 1H), 6.50–6.58 (m, 2H), 5.14 (s, 2H), 5.12 (s, 2H), 4.63 (dd, *J* =

11.7 Hz, *J* = 10.8 Hz, 1H), 4.51 (dd, *J* = 10.8 Hz, *J* = 5.1 Hz, 1H), 4.26 (dd, *J* = 11.7 Hz, *J* = 5.5 Hz, 1H), 3.80 (s, 3H), 3.44 (s, 3H) ppm; ^{13}C NMR (75 MHz, $CDCl_3$): δ 191.32 (C_q), 164.82 (C_q), 163.68 (C_q), 160.37 (C_q), 156.17 (C_q), 135.96 (C_q), 130.77 (CH), 129.39 (CH), 128.72 (CH), 128.30 (CH), 127.51 (CH), 116.40 (C_q), 115.71 (C_q), 110.41 (CH), 106.61 (CH), 101.76 (CH), 101.73 (CH), 94.61 (CH_2), 71.26 (CH_2), 70.29 (CH_2), 56.19 (CH_3), 55.40 (CH_3), 47.69 (CH) ppm; IR (ATR): 2915, 1604, 1238, 1154, 992, 834 cm^{-1}; GC-MS (EI): m/z = 420 $[M]^+$; analysis (calcd., found for $C_{25}H_{24}O_6$): C (71.42, 71.79), H (5.75, 6.13).

Supplementary Note 4

First-Generation Synthesis: Preparation of Isoflavanone 9

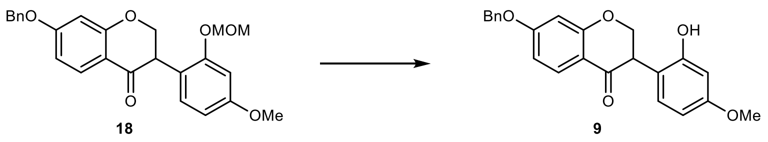

Isoflavanone **18** (731.8 mg, 1.74 mmol, 1.0 equiv.) was dissolved in THF (25 mL) and methanol (25 mL) in a 100 mL round-bottomed flask. Hydrochloric acid (8.7 mL, 6 M in water, 52 mmol, 30 equiv.) was added, and the reaction mixture was stirred at 60 °C for 35 min. The reaction mixture was cooled to room temperature, and saturated aqueous $NaHCO_3$ solution was added. The aqueous layer was extracted three times with EtOAc, the combined organic layers were dried over $MgSO_4$, and the solvents were removed in vacuo. The residue was purified by flash chromatography (DCM:EtOAc 20:1 *v/v*) to afford isoflavanone **9** (488.0 mg, 74%) as a white solid and starting material **18** (95.0 mg, 13%).

9: mp: 137–138 °C; TLC (isohexane:EtOAc 2:1 *v/v*): R_f = 0.43; ^1H NMR (300 MHz, $CDCl_3$): δ 8.37 (s, 1H), 7.86 (d, *J* = 8.9 Hz, 1H), 7.33–7.46 (m, 6H), 6.67 (dd, *J* = 9.0 Hz, *J* = 2.4 Hz, 1H), 6.55 (d, *J* = 2.6 Hz, 1H), 6.53 (d, *J* = 2.5 Hz, 1H), 6.47 (dd, *J* = 8.5 Hz, *J* = 2.6 Hz, 1H), 5.11 (s, 2H), 4.97 (dd, *J* = 11.9 Hz, *J* = 3.0 Hz, 1H), 4.82 (dd, *J* = 12.1 Hz, *J* = 4.5 Hz, 1H), 3.93

(dd, J = 4.5 Hz, J = 3.0 Hz, 1H), 3.76 (s, 3H) ppm; ^{13}C NMR (75 MHz, $CDCl_3$): δ 192.89 (C_q), 166.12 (C_q), 163.71 (C_q), 160.73 (C_q), 156.68 (C_q), 135.62 (C_q), 130.00 (CH), 128.75 (CH), 128.40 (CH), 127.49 (CH), 127.30 (CH), 115.01 (C_q), 112.79 (C_q), 111.32 (CH), 107.17 (CH), 103.44 (CH), 101.62 (CH), 70.42 (CH_2), 69.94 (CH_2), 55.29 (CH_3), 45.48 (CH) ppm; IR (ATR): 3201, 1435, 1243, 1166, 1107, 845, 730 cm^{-1}; MS (ESI): m/z = 377.5 $[M+H]^+$; analysis (calcd., found for $C_{23}H_{20}O_5$): C (73.39, 73.15), H (5.36, 5.46).

Supplementary Note 5

First-Generation Synthesis: Preparation of Isoflavan-4-ol 8 from Isoflavanone 9

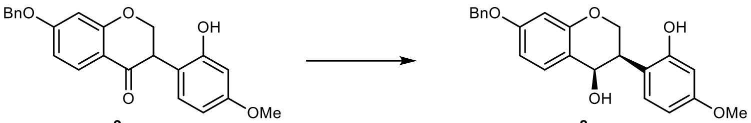

Preparation of the catalyst solution:

$[Ru(p\text{-cym})Cl_2]_2$ (22.3 mg, 36.4 µmol) and (*R*,*R*)-TsDPEN (27.3 mg, 72.8 µmol) were dissolved in EtOAc (2.2 mL) in a 5 mL round-bottomed flask. Another flask containing triethylamine (1.5 mL) was cooled to 0 °C, and formic acid (0.5 mL) was added. The mixture was stirred vigorously for 5 min at room temperature. An aliquot of this mixture (1.14 mL) was added to the ruthenium catalyst, and the resulting solution was stirred for another 5 min.

Reaction:

In a 5 mL round-bottomed flask isoflavanone **9** (450.9 mg, 1.20 mmol, 1.0 equiv.) was suspended in EtOAc (1.5 mL) at 45 °C, and an aliquot of the catalyst solution (2.73 mL, ca. 5 mol% catalyst) was added. The reaction mixture was stirred at 45 °C to 50 °C for 1.5 h, cooled to room temperature, and saturated aqueous NH_4Cl solution was added. The aqueous layer was extracted three times with EtOAc. The combined organic layers were dried over $MgSO_4$, the

solvents were removed in vacuo, and the residue was purified by flash chromatography (DCM:EtOAc 10:1 *v/v*) to afford isoflavanol **8** (403.2 mg, 89%, >99% *ee*) as a white solid.

mp: 141–142 °C; TLC (DCM:EtOAc 2:1 *v/v*): R_f = 0.39; $[\alpha]_D^{25}$ = +30.3 (*c* 1.0 $CHCl_3$); ^{1}H NMR (300 MHz, $CDCl_3$): δ 8.08 (s, 1H), 7.33–7.47 (m, 5H), 7.15 (d, *J* = 8.5 Hz, 1H), 7.02 (d, *J* = 8.3 Hz, 1H), 6.61 (dd, *J* = 8.4 Hz, *J* = 2.5 Hz, 1H), 6.53 (d, *J* = 2.5 Hz, 1H), 6.43–6.51 (m, 2H), 5.06 (s, 2H), 4.99 (br. s, 1H), 4.80 (dd, *J* = 12.4 Hz, *J* = 10.9 Hz, 1H), 4.21 (ddd, *J* = 10.8 Hz, *J* = 4.0 Hz, *J* = 1.1 Hz, 1H), 3.79 (s, 3H), 3.34 (dt, *J* = 12.1 Hz, *J* = 3.4 Hz, 1H), 2.47 (d, *J* = 2.5 Hz, 1H) ppm; ^{13}C NMR (75 MHz, $CDCl_3$): δ 160.61 (C_q), 160.51 (C_q), 156.43 (C_q), 155.12 (C_q), 136.64 (C_q), 132.17 (CH), 130.44 (CH), 128.62 (CH), 128.05 (CH), 127.43 (CH), 116.41 (C_q), 116.29 (C_q), 108.67 (CH), 106.71 (CH), 103.65 (CH), 102.63 (CH), 70.06 (CH_2), 68.67 (CH), 63.67 (CH_2), 55.30 (CH_3), 43.86 (CH) ppm; IR (ATR): 3490, 3360, 1164, 1018, 831, 731 cm^{-1}; MS (ESI): m/z = 377.5 $[M–H]^-$; analysis (calcd., found for $C_{23}H_{22}O_5$): C (73.00, 72.94), H (5.86, 6.22).

Supplementary Note 6

First-Generation Synthesis: Preparation of Isoflavone 10

Synthesis of Bromobenzene 34

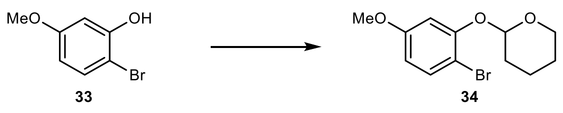

Bromophenol **33**[2] (1.001 g, 4.93 mmol, 1.0 equiv.) was dissolved in DCM (4.9 mL) in a 10 mL round-bottomed flask, and 3,4-dihydro-2*H*-pyran (1.8 mL, 19.7 mmol, 4.0 equiv.) and pyridinium *p*-toluenesulfonate (123.9 mg, 0.49 mmol, 0.1 equiv.) were added successively. The reaction mixture was stirred at room temperature for 2 h. Saturated aqueous $NaHCO_3$ solution was added, and the aqueous layer was extracted three times with DCM. The combined organic

layers were dried over $MgSO_4$, and the solvents were removed in vacuo. The residue was purified by flash chromatography (isohexane:EtOAc 4:1 *v/v*) to afford bromobenzene **34** (1.371 g, 97%) as a colourless liquid.

TLC (isohexane:EtOAc 4:1 *v/v*): R_f = 0.56; ^{1}H NMR (300 MHz, $CDCl_3$): δ 7.41 (d, *J* = 8.8 Hz, 1H), 6.77 (d, *J* = 2.8 Hz, 1H), 6.46 (dd, *J* = 8.8 Hz, *J* = 2.8 Hz, 1H), 5.49 (t, *J* = 2.8 Hz, 1H), 3.91 (td, *J* = 11.1 Hz, *J* = 3.0 Hz, 1H), 3.78 (s, 3H), 3.59–3.65 (m, 1H), 2.05–2.17 (m, 1H), 1.95–2.01 (m, 1H), 1.83–1.92 (m, 1H), 1.59–1.78 (m, 3H) ppm; ^{13}C NMR (126 MHz, $CDCl_3$): δ 159.92 (C_q), 154.02 (C_q), 132.95 (CH), 107.85 (CH), 103.76 (C_q), 103.56 (CH), 96.75 (CH), 61.78 (CH_2), 55.51 (CH_3), 30.14 (CH_2), 25.18 (CH_2), 18.27 (CH_2) ppm; IR (ATR): 2942, 1199, 1165, 1020, 965, 900 cm^{-1}; GC-MS (EI): m/z = 286 $[M]^+$; analysis (calcd., found for $C_{12}H_{15}BrO_3$): C (50.19, 50.53), H (5.27, 5.62).

Suzuki Coupling of Iodide 15 and the Boronic Acid 19

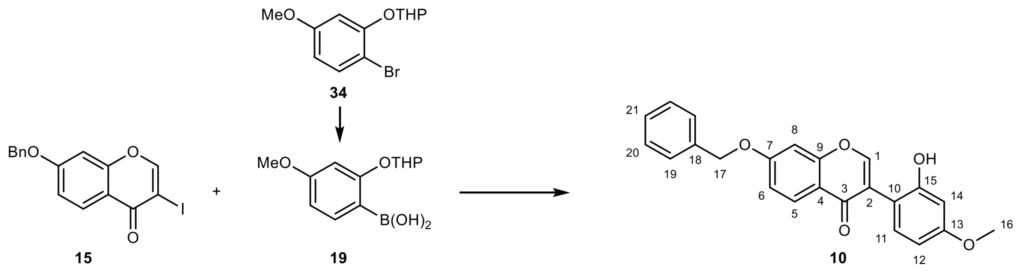

Bromobenzene **34** (961.5 mg, 3.35 mmol, 1.5 equiv.) was dissolved in THF (6.7 mL) and triisopropyl borate (1.6 mL, 6.70 mmol, 3.0 equiv.) in a 10 mL round-bottomed flask and cooled to –78 °C. *n*-Butyllithium solution (3.8 mL, 1.6 M in hexanes, 6.03 mmol, 2.7 equiv.) was added over 15 min, and the reaction mixture was stirred for 45 min at –78 °C. The dry-ice/acetone bath was removed, and the reaction mixture was stirred for 1.5 h and then poured onto saturated aqueous NH_4Cl solution. The aqueous layer was extracted three times with EtOAc, the combined organic layers were washed twice with brine and dried over $MgSO_4$, and the solvents were removed in vacuo. To the crude boronic acid was added iodide **15** (844.2 mg, 2.23 mmol,

1.0 equiv.), potassium carbonate (925.6 mg, 6.70 mmol, 3.0 equiv.), BHT (4.9 mg, 22.3 µmol, 1 mol%), 1,4-dioxane (9.2 mL), and water (3.9 mL). The mixture was purged with argon for 5 min. Tricyclohexylphosphine (31.3 mg, 0.11 mmol, 5 mol%) and $Pd_2(dba)_3$ (51.1 mg, 55.8 µmol, 2.5 mol%) were added successively, and the reaction mixture was stirred at 50 °C for 1 h. Concentrated hydrochloric acid (2.2 mL) was added carefully and the reaction mixture was stirred for 10 min at 50 °C. The reaction mixture was cooled to room temperature and diluted with water. The aqueous layer was extracted three times with DCM, the combined organic layers were dried over $MgSO_4$, and the solvents were removed in vacuo. The residue was suspended in pentane (40 mL), and the product **10** was filtered off (655.0 mg, 78%) as a yellowish solid.

mp: 170–172 °C; TLC (DCM:isohexane:EtOAc 10:5:1 *v/v*): R_f = 0.37; ^{1}H NMR (600 MHz, $CDCl_3$): δ 9.33 (s, 1H, OH-15), 8.28 (d, 1H, $^3J_{5/6}$ = 9.0 Hz, H-5), 8.05 (s, 1H, H-1), 7.36–7.49 (m, 5H, H-19, H-20, H-21), 7.15 (dd, 1H, $^3J_{6/5}$ = 9.0 Hz, $^4J_{6/8}$ = 2.3 Hz, H-6), 7.07 (d, 1H, $^3J_{11/12}$ = 8.7 Hz, H-11), 7.00 (d, 1H, $^4J_{8/6}$ = 2.3 Hz, H-8), 6.66 (d, 1H, $^4J_{14/12}$ = 2.6 Hz, H-14), 6.55 (dd, 1H, $^3J_{12/11}$ = 8.5 Hz, $^4J_{12/14}$ = 2.4 Hz, H-12), 5.21 (s, 2H, H-17), 3.83 (s, 3H, H-16) ppm; ^{13}C NMR (151 MHz, $CDCl_3$): δ 178.76 (C_q, C-3), 163.76 (C_q, C-7), 161.98 (C_q, C-13), 157.94 (C_q, C-9), 157.76 (C_q, C-15), 154.60 (CH, C-1), 135.41 (C_q, C-18), 130.18 (CH, C-11), 128.82 (CH, C-20), 128.52 (CH, C-21), 127.92 (CH, C-5), 127.53 (CH, C-19), 124.88 (C_q, C-2), 117.19 (C_q, C-4), 116.15 (CH, C-6), 112.86 (C_q, C-10), 107.63 (CH, C-12), 104.30 (CH, C-14), 100.83 (CH, C-8), 70.72 (CH_2, C-17), 55.36 (CH_3, C-16) ppm; IR (ATR): 1542, 1235, 1198, 843, 806, 690 cm^{-1}; MS (ESI): m/z = 375.1 $[M+H]^+$; analysis (calcd., found for $C_{23}H_{18}O_5$): C (73.79, 73.73), H (4.85, 4.81).

Supplementary Note 7

First-Generation Synthesis: ATH Cascade from Isoflavone 10 to Isoflavanol 8

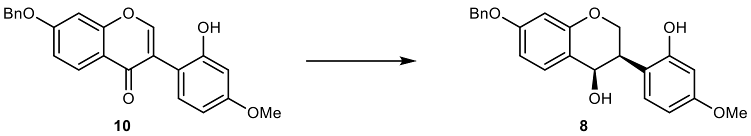

Preparation of the catalyst solution:

[Ru(*p*-cym)Cl_2]$_2$ (4.3 mg, 7.0 µmol) and (*R*,*R*)-TsDPEN (7.8 mg, 21.3 µmol) were dissolved in EtOAc (0.42 mL) in a 5 mL round-bottomed flask. Another flask containing triethylamine (1.5 mL) was cooled to 0 °C, and formic acid (0.5 mL) was added. The mixture was stirred vigorously for 5 min at room temperature. An aliquot of this mixture (1.1 mL) was added to the ruthenium catalyst, and the resulting solution was stirred for another 5 min.

Reaction:

In a 5 mL round-bottomed flask isoflavone **10** (359.1 mg, 0.96 mmol, 1.0 equiv.) was suspended in EtOAc (1.2 mL) at 45 °C, and an aliquot of the catalyst solution (1.04 mL, ca. 1 mol% catalyst) was added. The reaction mixture was stirred at 45 °C for 17 h, cooled to room temperature, and saturated aqueous NH_4Cl solution was added. The aqueous layer was extracted three times with EtOAc. The combined organic layers were dried over $MgSO_4$, the solvents were removed in vacuo, and the residue was purified by flash chromatography (DCM:EtOAc 10:1 *v/v*) to afford isoflavanol **8** (333.1 mg, 92%, >99% *ee*) as a white solid.

For analytical data, see Supplementary Note 5.

Supplementary Note 8

First-Generation Synthesis: Preparation of Vestitol (7)

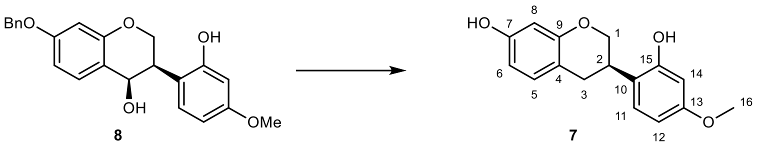

Isoflavanol **8** (52.5 mg, 0.14 mmol, 1.0 equiv.) was dissolved in dry ethanol (6.0 mL) in a 10 mL round-bottomed flask, and 10% Pd/C (14.8 mg, 13.9 µmol, 0.1 equiv.) was added. The reaction mixture was stirred under hydrogen atmosphere (balloon) for 23.5 h at room temperature. The mixture was filtered through a short pad of celite, and the solvent was removed in vacuo. The residue was purified by flash chromatography (DCM:EtOAc 10:1 *v/v*) to afford vestitol (**7**, 29.2 mg, 77%, 97% *ee*) as a white solid.

mp: 151–153 °C (lit.[3] 144–145 °C); TLC (DCM:EtOAc 5:1 *v/v*): R_f = 0.38; $[\alpha]_D^{20}$ = –9.4 (*c* 0.42 MeOH) (lit.[3] $[\alpha]_D^{20}$ = –5 (*c* 0.27 MeOH), lit.[4] $[\alpha]_D^{22}$ = –18.9 (*c* 0.5 MeOH)); ^{1}H NMR (600 MHz, acetone-d_6): δ 8.49 (s, 1H, OH-15), 8.06 (s, 1H, OH-7), 7.05 (d, 1H, $^3J_{11/12}$ = 8.3 Hz, H-11), 6.89 (d, 1H, $^3J_{5/6}$ = 8.3 Hz, H-5), 6.50 (d, 1H, $^4J_{14/12}$ = 2.6 Hz, H-14), 6.43 (dd, 1H, $^3J_{12/11}$ = 8.5 Hz, $^4J_{12/14}$ = 2.4 Hz, H-12), 6.36 (dd, 1H, $^3J_{6/5}$ = 8.1 Hz, $^4J_{6/}$ = 2.4 Hz, H-6), 6.28 (d, 1H, $^4J_{8/6}$ = 2.6 Hz, H-8), 4.24 (ddd, 1H, $^2J_{1a/1b}$ = 10.4 Hz, $^3J_{1a/2}$ = 3.4Hz, $^4J_{1a/3b}$ = 2.1 Hz, H-1a), 3.98 (t, 1H, $^2J_{1b/1a}$ = $^3J_{1b/2}$ = 10.2 Hz, H-1b), 3.72 (s, 3H, H-16), 3.48 (dddd, 1H, $^3J_{2/3a}$ = 10.9 Hz, $^3J_{2/1b}$ = 10.2 Hz, $^3J_{2/3b}$ = 4.9 Hz, $^3J_{2/1a}$ = 3.4 Hz, H-2), 2.97 (ddd, 1H, $^2J_{3a/3b}$ = 15.4 Hz, $^3J_{3a/2}$ = 10.9 Hz, $^4J_{3a/1b}$ = 0.8 Hz, H-3a), 2.81 (ddd, 1H, $^2J_{3b/3a}$ = 15.5 Hz, $^3J_{3b/2}$ = 5.0 Hz, $^4J_{3b/1a}$ = 2.1 Hz, H-3b) ppm; ^{13}C NMR (151 MHz, acetone-d_6): δ 160.49 (C_q, C-13), 157.60 (C_q, C-7), 156.79 (C_q, C-15), 156.20 (C_q, C-9), 131.10 (CH, C-5), 128.84 (CH, C-11), 121.02 (C_q, C-10), 114.41 (C_q, C-4), 108.84 (CH, C-6), 105.78 (CH, C-12), 103.76 (CH, C-8), 102.60 (CH, C-14), 70.58 (CH_2, C-1), 55.45 (CH_3, C-16), 32.71 (CH, C-2), 31.15 (CH_2, C-3) ppm; IR (ATR): 3350, 1141,

1114, 1031, 794 cm^{-1}; MS (ESI): m/z = 273.1 $[M+H]^+$; analysis (calcd., found for $C_{16}H_{16}O_4$): C (70.58, 70.84), H (5.92, 5.89).

Supplementary Note 9

First-Generation Synthesis: Preparation of Isoflavan-4-ol 20

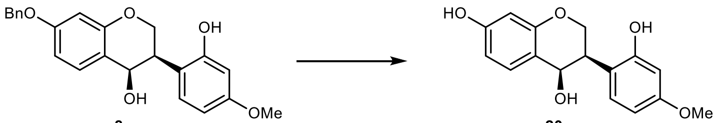

Isoflavanol **8** (46.8 mg, 0.12 mmol, 1.0 equiv.) was dissolved in dry ethanol (6.2 mL) in a 10 mL round-bottomed flask, and 10% Pd/C (13.2 mg, 12.4 µmol, 0.1 equiv.) and pyridine (0.01 mL, 0.14 mmol, 1.1 equiv.) were added successively. The reaction mixture was stirred under hydrogen atmosphere (balloon) for 1.8 h at room temperature. The mixture was filtered through a short pad of celite, and the solvent was removed in vacuo. The residue was purified by flash chromatography (DCM:EtOAc 5:1 *v/v*) to afford isoflavanol **20** (41.3 mg, ≈96%, >99% *ee*) as a white solid with small impurities.

mp: 181–183 °C; TLC (DCM:EtOAc 5:1 *v/v*): R_f = 0.18; $[\alpha]_D^{20}$ = +148.8 (*c* 0.4 acetone); ^{1}H NMR (300 MHz, acetone-d_6): δ 8.87 (s, 1H), 8.31 (s, 1H), 7.11 (d, *J* = 8.3 Hz, 1H), 7.08 (d, *J* = 8.3 Hz, 1H), 6.45 (d, *J* = 2.6 Hz, 1H), 6.37–6.43 (m, 2H), 6.30 (d, *J* = 2.5 Hz, 1H), 4.82 (br. s., 1H), 4.62 (dd, *J* = 12.5 Hz, *J* = 10.2 Hz, 1H), 4.57 (br. s., 1H), 4.10 (ddd, *J* = 10.2 Hz, *J* = 3.6 Hz, *J* = 1.3 Hz, 1H), 3.73 (s, 3H), 3.46 (dt, *J* = 12.2 Hz, *J* = 3.3 Hz, 1H) ppm; ^{13}C NMR (75 MHz, acetone-d_6): δ 160.78 (C_q), 159.38 (C_q), 157.34 (C_q), 156.13 (C_q), 132.32 (CH), 131.51 (CH), 118.57 (C_q), 118.17 (C_q), 108.88 (CH), 105.78 (CH), 103.50 (CH), 102.93 (CH), 66.90 (CH), 64.83 (CH_2), 55.44 (CH_3), 40.78 (CH) ppm; IR (ATR): 3551, 3424, 3190, 1444, 1152, 1104, 1029, 821, 798 cm^{-1}; MS (ESI): m/z = 286.7 $[M–H]^-$; analysis (calcd., found for $C_{16}H_{16}O_5$): C (66.66, 66.40), H (5.59, 5.57).

Supplementary Note 10

First-Generation Synthesis: Preparation of Medicarpin (5) from Isoflavan-4-ol 8

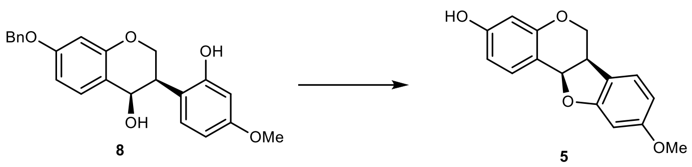

Isoflavanol **8** (720.0 mg, 1.85 mmol, 1.0 equiv.) was dissolved in dry ethanol (90 mL) in a 250 mL round-bottomed flask, and 10% Pd/C (202.5 mg, 0.19 mmol, 0.1 equiv.) and pyridine (0.17 mL, 2.0 mmol, 1.1 equiv.) were added successively. The reaction mixture was stirred under hydrogen atmosphere (balloon) for 50 min at room temperature. The hydrogen atmosphere was replaced by argon, hydrochloric acid (0.21 mL) was added, and the reaction mixture was stirred for 20 min at room temperature. The mixture was filtered through a short pad of celite/$NaHCO_3$ (1:1), and the solvent was removed in vacuo. The residue was purified by flash chromatography (DCM:EtOAc 10:1 *v/v*) to afford medicarpin (**5**, 449.9 mg, 90%, >99% *ee*) as a white solid.

mp: 128–129 °C (lit.[5] 193 °C); TLC (DCM:EtOAc 10:1 *v/v*): R_f = 0.46; $[\alpha]_D^{20}$ = –196.4 (*c* 0.05 $CHCl_3$) (lit.[5] $[\alpha]_D^{20}$ = –188 (*c* 0.106 $CHCl_3$)); ^{1}H NMR (500 MHz, $CDCl_3$): δ 7.39 (d, *J* = 8.5 Hz, 1H), 7.12–7.16 (m, 1H), 6.55 (dd, *J* = 8.5 Hz, *J* = 2.5 Hz, 1H), 6.45–6.49 (m, 2H), 6.42 (d, *J* = 2.5 Hz, 1H), 5.51 (d, *J* = 6.9 Hz, 1H), 5.38 (br. s, 1H), 4.25 (dd, *J* = 11.0 Hz, *J* = 4.7 Hz, 1H), 3.78 (s, 3H), 3.64 (t, *J* = 11.0 Hz, 1H), 3.54 (ddd, *J* = 11.0 Hz, *J* = 6.5 Hz, *J* = 4.9 Hz, 1H) ppm; ^{13}C NMR (126 MHz, $CDCl_3$): δ 161.03 (C_q), 160.57 (C_q), 157.00 (C_q), 156.58 (C_q), 132.18 (CH), 124.75 (CH), 119.09 (C_q), 112.53 (C_q), 109.78 (CH), 106.41 (CH), 103.64 (CH), 96.89 (CH), 78.53 (CH), 66.49 (CH_2), 55.50 (CH_3), 39.42 (CH) ppm; IR (ATR): 3399, 1454, 1145, 1016, 941, 822, 789 cm^{-1}; GC-MS (EI): m/z = 270 $[M]^+$.

Supplementary Note 11

First-Generation Synthesis: Preparation of Homopterocarpin (4)

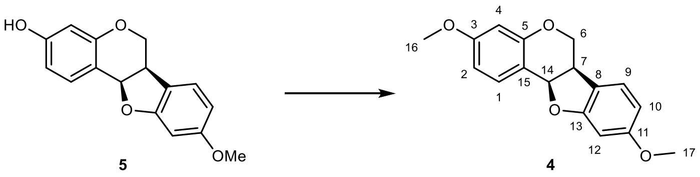

Medicarpin (**5**, 236.3 mg, 0.87 mmol, 1.0 equiv.) was dissolved in acetone (8.7 mL) in a 10 mL round-bottomed flask. Potassium carbonate (483.3 mg, 3.50 mmol, 4.0 equiv.) and iodomethane (0.22 mL, 3.50 mmol, 4.0 equiv.) were added successively. The reaction flask was sealed and heated to 56 °C for 23.5 h. The reaction mixture was cooled to room temperature, and 0.5 M hydrochloric acid was added. The aqueous layer was extracted three times with EtOAc, and the combined organic layers were dried over $MgSO_4$. The solvents were removed in vacuo, and the residue was purified by flash chromatography (DCM:EtOAc 15:1 *v/v*) to afford homopterocarpin (**4**, 238.7 mg, 96%, >99% *ee*) as a white solid.

mp: 85–86 °C (lit.[6] 86 °C); TLC (DCM:EtOAc 15:1 *v/v*): R_f = 0.63; $[\alpha]_D^{20}$ = –211.2 (*c* 1.16 $CHCl_3$) (lit.[6] $[\alpha]_D^{25}$ = –205 (*c* 0.02 $CHCl_3$)); ^{1}H NMR (600 MHz, $CDCl_3$): δ 7.44 (d, 1H, $^3J_{1/2}$ = 8.5 Hz, H-1), 7.14 (d, 1H, $^3J_{9/10}$ = 8.8 Hz, H-9), 6.65 (dd, 1H, $^3J_{2/1}$ = 8.6 Hz, $^4J_{2/4}$ = 2.5 Hz, H-2), 6.48 (d, 1H, $^4J_{4/2}$ = 2.4 Hz, H-4), 6.44–6.47 (m, 2H, H-10, H-12), 5.52 (d, 1H, $^3J_{14/7}$ = 6.8 Hz, H-14), 4.26 (dd, 1H, $^2J_{6a/6b}$ = 11.1 Hz, $^3J_{6a/7}$ = 5.1 Hz, H-6a), 3.80 (s, 3H, H-16/H-17), 3.78 (s, 3H, H-16/H-17), 3.65 (t, 1H, $^2J_{6b/6a}$ = $^3J_{6b/7}$ = 10.5 Hz, H-6b), 3.55 (ddd, $^3J_{7/6b}$ = 11.2 Hz, $^3J_{7/14}$ = 6.5 Hz, $^3J_{7/6a}$ = 5.1 Hz, H-7) ppm; ^{13}C NMR (151 MHz, $CDCl_3$): δ 161.13 (C_q, C-3/C-11/C-13), 161.03 (C_q, C-3/C-11/C-13), 160.72 (C_q, C-3/C-11/C-13), 156.61 (C_q, C-5), 131.82 (CH, C-1), 124.71 (CH, C-9), 119.12 (C_q, C-8), 112.34 (C_q, C-15), 109.16 (CH, C-2), 106.35 (CH, C-10), 101.62 (CH, C-4), 96.90 (CH, C-12), 78.57 (CH-14), 66.58 (CH_2, C-6), 55.49 (CH_3, C-16/C-17), 55.37 (CH_3, C-16/C-17), 39.54 (CH, C-7) ppm; IR (ATR): 2904, 1620, 1494, 1158,

1116, 1027, 837, 798, 635 cm^{-1}; GC-MS (EI): m/z = 284 $[M]^+$; analysis (calcd., found for $C_{17}H_{16}O_4$): C (71.82, 72.09), H (5.67, 5.75).

Supplementary Note 12

First-Generation Synthesis: Preparation of Variabilin (3) and Isosativanone (21)

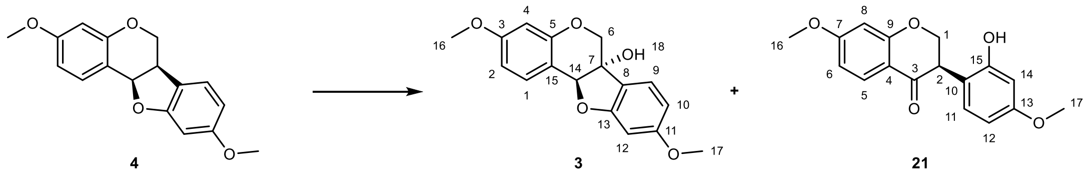

Homopterocarpin (**4**, 97.5 mg, 0.34 mmol, 1.0 equiv.), *N*-hydroxyphthalimide (55.9 mg, 0.34 mmol, 1.0 equiv.) and cobalt(II) acetate (30.4 mg, 0.17 mmol, 0.5 equiv.) were stirred in MeCN (6.9 mL) in a 20 mL test tube with ground joint at room temperature for 2 h under argon atmosphere. The reaction mixture was cooled to 0 °C and stirred for 24 h under oxygen atmosphere (balloon). The oxygen atmosphere was replaced by argon, PPh_3 (134.9 mg, 0.51 mmol, 1.5 equiv.) was added, and the reaction was stirred for 20 min at room temperature. Saturated aqueous NH_4Cl solution was added, and the aqueous layer was extracted three times with EtOAc. The combined organic layers were dried over $MgSO_4$, the solvents were removed in vacuo, and the residue was purified by flash chromatography (DCM:EtOAc 10:1 *v/v*) to afford a mixture of **3** and **21**. The mixture was separated by preparative HPLC (DCM:EtOAc 15:1 *v/v*) to afford variabilin (**3**, 65.9 mg, 64%, >99% *ee*) as a semi-crystalline opaque gum and isosativanone (**21**, 10.3 mg, 10%, 91% *ee*) as a yellowish solid.

Variabilin (**3**): mp: 115–116 °C; TLC (DCM:EtOAc 15:1 *v/v*): R_f = 0.33; $[\alpha]_D^{20}$ = –284.8 (*c* 0.8 $CHCl_3$) (lit.[7] $[\alpha]_D^{25}$ = –220.8 (*c* 0.68 $CHCl_3$)); ^{1}H NMR (600 MHz, $CDCl_3$): δ 7.41 (d, 1H, $^3J_{1/2}$ = 8.7 Hz, H-1), 7.27 (d, 1H, $^3J_{9/10}$ = 8.3 Hz, H-9), 6.67 (dd, 1H, $^3J_{2/1}$ = 8.6 Hz, $^4J_{2/4}$ = 2.5 Hz, H-2), 6.54 (dd, 1H, $^3J_{10/9}$ = 8.4 Hz, $^4J_{10/12}$ = 2.2 Hz, H-10), 6.46 (d, 1H, $^4J_{4/2}$ = 2.6 Hz, H-4),

6.42 (d, 1H, $^4J_{12/10}$ = 2.3 Hz, H-12), 5.32 (s, 1H, H-14), 4.22 (dd, 1H, $^2J_{6a/6b}$ = 11.6 Hz, $^4J_{6a/14}$ = 0.8 Hz, H-6a), 4.03 (d, 1H, $^2J_{6b/6a}$ = 11.5 Hz, H-6b), 3.78 (s, 3H, H-16/H-17), 3.77 (s, 3H, H-17/ H-16), 2.42 (s, 1H, OH-7) ppm; ^{13}C NMR (151 MHz, $CDCl_3$): δ 162.53 (C_q, C-11), 161.05 (C_q, C-3), 160.88 (C_q, C-13), 155.76 (C_q, C-5), 131.86 (CH, C-1), 123.82 (CH, C-9), 120.04 (C_q, C-8), 112.36 (C_q, C-15), 109.79 (CH, C-2), 107.68 (CH, C-10), 101.64 (CH, C-4), 97.06 (CH, C-12), 84.91 (CH, C-14), 76.74 (C_q, C-7), 69.68 (CH_2, C-6), 55.52 (CH_3, C-16/C-17), 55.37 (CH_3, C-17/16) ppm; IR (ATR): 3440, 2920, 1620, 1497, 1270, 1129, 1030, 958, 830 cm^{-1}; GC-MS (EI): m/z = 300 $[M]^+$.

Isosativanone **21**: mp: 120–123 °C; TLC (DCM:EtOAc 15:1 *v/v*): R_f = 0.33; $[\alpha]_D^{20}$ = +333.5 (*c* 0.23 $CHCl_3$); ^{1}H NMR (600 MHz, $CDCl_3$): δ 8.42 (s, 1H, OH-15), 7.85 (d, 1H, $^3J_{5/6}$ = 9.0 Hz, H-5), 7.44 (d, 1H, $^3J_{11/12}$ = 8.7 Hz, H-11), 6.60 (dd, 1H, $^3J_{6/5}$ = 8.8 Hz, $^4J_{6/8}$ = 1.7 Hz, H-6), 6.55 (d, 1H, $^4J_{14/12}$ = 2.3 Hz, H-14), 6.48 (dd, 1H, $^3J_{12/11}$ = 8.7 Hz, $^4J_{12/14}$ = 2.3 Hz, H-12), 6.45 (d, 1H, $^4J_{8/6}$ = 2.3 Hz, H-8), 4.98 (dd, 1H, $^2J_{1a/1b}$ = 12.0 Hz, $^3J_{1a/2}$ = 2.3 Hz, H-1a), 4.83 (dd, 1H, $^2J_{1b/1a}$ = 12.0 Hz, $^3J_{1b/2}$ = 4.5 Hz, H-1b), 3.93 (dd, 1H, $^3J_{2/1b}$ = 4.5 Hz, $^3J_{2/1a}$ = 2.3 Hz, H-2), 3.86 (s, 3H, H-16), 3.76 (s, 3H, H-17) ppm; ^{13}C NMR (151 MHz, $CDCl_3$): δ 192.90 (C_q, C-3), 167.07 (C_q, C-7), 163.80 (C_q, C-9), 160.73 (C_q, C-13), 156.71 (C_q, C-15), 129.95 (CH, C-5), 127.24 (CH, C-11), 115.06 (C_q, C-10), 112.56 (C_q, C-4), 110.89 (CH, C-6), 107.18 (CH, C-12), 103.44 (CH, C-14), 100.56 (CH, C-8), 69.92 (CH_2, C-1), 55.74 (CH_3, C-16), 55.29 (CH_3, C-17), 45.43 (CH, C-2) ppm; IR (ATR): 3329, 1596, 1437, 1240, 1199, 1160, 1103, 1016, 823 cm^{-1}; MS (ESI): m/z = 301.1 $[M+H]^+$; analysis (calcd., found for $C_{17}H_{16}O_5$): C (67.99, 67.78), H (5.37, 5.35).

Supplementary Note 13

Second-Generation Synthesis: One-pot Transformation from 2’-Hydroxydaidzein (23) to 3,9-Dihydroxypterocarpan (6)

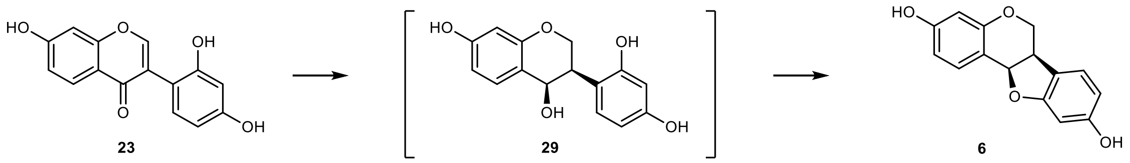

Preparation of the catalyst solution:

[Ru(*p*-cym)Cl_2]$_2$ (22.6 mg, 36.9 µmol) and (*R*,*R*)-TsDPEN (30.4 mg, 81.2 µmol) were dissolved in DMSO (0.44 mL) in a 5 mL round-bottomed flask. Another flask containing triethylamine (1.8 mL) was cooled to 0 °C, and formic acid (0.6 mL) was added. The mixture was stirred vigorously for 5 min at room temperature. An aliquot of this mixture (1.2 mL) was added to the ruthenium catalyst, and the resulting solution was stirred for another 3 min.

Reaction:

In a 5 mL round-bottomed flask isoflavone **23** (271.3 mg, 1.00 mmol, 1.0 equiv., for preparation, see Supplementary Note 16) was dissolved in DMSO (1.25 mL), and water (0.18 mL, 10.4 mmol, 10 equiv.) and an aliquot of the catalyst solution (1.1 mL, ca. 5 mol% catalyst) were added. The reaction mixture was stirred at 45 °C for 19.5 h, cooled to room temperature, and hydrochloric acid (37%, 0.52 mL, 6.24 mmol, 6.2 equiv.) was added. The mixture was stirred for 10 min. The reaction was quenched by addition of saturated aqueous NH_4Cl solution, and the mixture was extracted three times with EtOAc. The combined organic layers were dried over $MgSO_4$, the solvents were removed in vacuo, and the residue was purified by flash chromatography (DCM:EtOAc 3:1 *v/v*) to afford pterocarpan **6** (187.8 mg, 73%, 99% *ee*) as a white solid foam.

3,9-Dihydroxypterocarpan (**6**): mp: 171–172 °C (lit.[8] 166–168 °C); TLC (DCM:EtOAc 1:1 *v/v*): R_f = 0.64; $[\alpha]_D^{20}$ = −240.8 (*c* 0.47 acetone); ^{1}H NMR (500 MHz, acetone-d_6): δ 8.56 (s,

1H), 8.33 (s, 1 H), 7.32 (d, *J* = 8.4 Hz, 1H), 7.13 (d, *J* = 8.0 Hz, 2H), 6.55 (dd, *J* = 8.4, *J* = 2.4 Hz, 1H), 6.33–6.40 (m, 2H), 6.28 (d, *J* = 2.0 Hz, 1H), 5.47 (d, *J* = 6.0 Hz, 1H), 4.20–4.29 (m, 1H), 3.50–3.59 (m, 2H) ppm; ^{13}C NMR (126 MHz, acetone-d_6): δ 161.84 (C_q), 159.72 (C_q), 159.63 (C_q), 157.78 (C_q), 133.19 (CH), 125.99 (CH), 119.30 (C_q), 112.97 (C_q), 110.51 (CH), 108.34 (CH), 103.98 (CH), 98.60 (CH), 79.34 (CH), 67.26 (CH_2), 40.39 (CH) ppm; IR (ATR): 3335, 1625, 1496, 1291, 1149, 1111, 956, 828, 792, 627 cm^{-1}; MS (ESI): m/z = 257.3 $[M+H]^+$; analysis (calcd., found for $C_{15}H_{12}O_4$): C (70.31, 70.33), H (4.72, 4.76).

Without addition of hydrochloric acid to the reaction mixture, the intermediate isoflavanol **29** may be isolated as an off-white solid (70% yield).

2’,4’,7-Trihydroxyisoflavan-4-ol (**29**): mp: 161–162 °C; TLC (DCM:EtOAc 1:1 *v/v*): R_f = 0.32; $[\alpha]_D^{20}$ = +137.9 (*c* 0.89 acetone); ^{1}H NMR (600 MHz, acetone-d_6): δ 8.74 (s, 1H), 8.30 (s, 1H), 8.09 (s, 1H), 7.07 (d, *J* = 8.3 Hz, 1H), 7.01 (d, *J* = 8.3 Hz, 1H), 6.38–6.41 (m, 2H), 6.31 (dd, *J* = 8.3 Hz, *J* = 2.6 Hz, 1H), 6.29 (d, *J* = 2.3 Hz, 1H), 4.80 (br. t, *J* = 3.0 Hz, 1H), 4.60 (dd, *J* = 12.4 Hz, *J* = 10.2 Hz, 1H), 4.50 (d, *J* = 4.1 Hz, 1H), 4.09 (ddd, *J* = 10.2 Hz, *J* = 3.6 Hz, *J* = 1.3 Hz, 1H), 3.44 (dt, *J* = 12.2 Hz, *J* = 3.1 Hz, 1H) ppm; ^{13}C NMR (151 MHz, acetone-d_6): δ 159.37 (C_q), 158.32 (C_q), 157.34 (C_q), 156.16 (C_q), 132.35 (CH), 131.46 (CH), 118.22 (C_q), 117.27 (C_q), 108.86 (CH), 107.66 (CH), 104.15 (CH), 103.50 (CH), 66.96 (CH), 64.91 (CH_2), 40.74 (CH) ppm; IR (ATR): 3478, 3364, 1600, 1163, 1113, 1033, 843, 798, 629 cm^{-1}; MS (ESI): m/z = 272.7 $[M–H]^–$; analysis (calcd., found for $C_{15}H_{14}O_5$): C (65.69, 65.75), H (5.15, 5.11).

Supplementary Note 14

Second-Generation Synthesis: Preparation of Homopterocarpin (4) from Pterocarpan 6

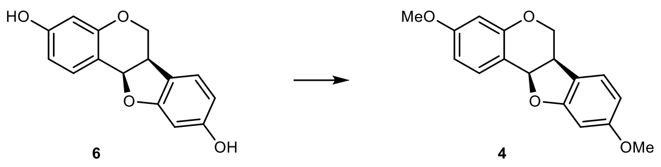

Pterocarpan **6** (82.7 mg, 0.32 mmol, 1.0 equiv.) was dissolved in DMF (1.6 mL) in a 10 mL round-bottomed flask and cooled to 0 °C. Potassium carbonate (178.5 mg, 1.29 mmol, 4.0 equiv.) and iodomethane (0.08 mL, 1.29 mmol, 4.0 equiv.) were added successively, and the reaction mixture was stirred at room temperature for 4 h. 2 N hydrochloric acid (1.5 mL), water, and EtOAc were added successively. The aqueous layer was extracted twice with EtOAc, the combined organic layers were dried over $MgSO_4$, the solvents were removed in vacuo, and the residue was purified by flash chromatography (DCM:EtOAc 15:1 *v/v*) to afford pterocarpan **4** (89.1 mg, 97%, 99% *ee*) as a white solid.

For analytical data, see Supplementary Note 11; for oxidation to variabilin (**3**), see Supplementary Note 12.

Supplementary Note 15

Synthesis of 1 and 2: Preparation of 7-Hydroxy-2',4'-dimethoxyisoflavone (22)

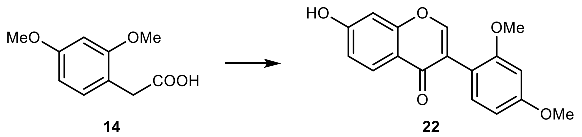

(2,4-Dimethoxyphenyl)acetic acid (**14**, 1.962 g, 10.0 mmol) and resorcinol (1.652 mmol, 15.0 mmol, 1.5 equiv.) were suspended in boron trifluoride diethyl etherate (7.4 mL, 60.0 mmol, 6.0 equiv.) in a 50 mL round-bottomed flask and stirred at 65 °C for 5 h. The reaction mixture was cooled in an ice-bath, and DMF (15.4 mL) was added carefully. The mixture was stirred at

room temperature for 15 min. The rubber septum was removed, and the open reaction flask was heated to 50 °C. At this temperature, mesyl chloride (2.3 mL, 30.0 mmol, 3.0 equiv.) in DMF (4.0 mL) was added over 15 min, and the resulting solution was heated to 75 °C for 17.5 h. The reaction was cooled to room temperature, poured into sodium acetate solution (200 mL, 12 g NaOAc/100 mL), and stirred for 1.5 h. The red solid was collected and refluxed in chloroform:ethanol 1:1 *v/v* (30 mL) for 40 min. After cooling to room temperature, the solid was collected by filtration and dried in vacuo to afford isoflavone **22** (1.912 g, 64%) as an off-white to orange solid.

mp: 260–272 °C (lit.[9] 265–270 °C); TLC (DCM:EtOAc 4:1 *v/v*): R_f = 0.28; ^{1}H NMR (300 MHz, DMSO-d_6): δ 10.75 (s, 1H), 8.12 (s, 1H), 7.91 (d, J = 8.7 Hz, 1H), 7.13 (d, J = 8.3 Hz, 1H), 6.92 (dd, J = 8.8 Hz, J = 2.2 Hz, 1H), 6.86 (d, J = 2.1 Hz, 1H), 6.63 (d, J = 2.1 Hz, 1H), 6.56 (dd, J = 8.3 Hz, J = 2.3 Hz, 1H), 3.80 (s, 3H), 3.70 (s, 3H) ppm; ^{13}C NMR (75 MHz, DMSO-d_6): δ 174.38 (C_q), 162.42 (C_q), 160.66 (C_q), 158.42 (C_q), 157.50 (C_q), 153.88 (CH), 132.03 (CH), 127.17 (CH), 121.60 (C_q), 116.55 (C_q), 115.02 (CH), 113.50 (C_q), 104.57 (CH), 102.13 (CH), 98.58 (CH), 55.53 (CH_3), 55.27 (CH_3) ppm; IR (ATR): 3191, 1572, 1236, 1157, 1031, 745 cm^{-1}; GC-MS (EI): m/z = 298.1 [M]$^+$; analysis (calcd., found for $C_{17}H_{14}O_5$): C (68.45, 68.57), H (4.73, 4.85).

Supplementary Note 16

Synthesis of 1 and 2: Preparation of 2’,4’,7-Trihydroxyisoflavone 23

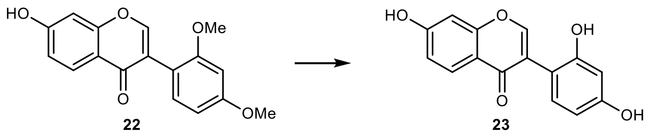

Isoflavone **22** (2.305 g, 7.73 mmol) was suspended in DCM (62 mL) in a 100 mL round-bottomed flask and cooled to 0 °C. Boron tribromide (3.7 mL, 38.6 mmol, 5.0 equiv.) was added

within 1 min and the reaction was stirred for 3 h at 0 °C. The reaction was quenched carefully with methanol (10 mL) and transferred to a separatory funnel with water and EtOAc. The mixture was extracted six times with EtOAc, and the solvents were removed in vacuo. The residue was suspended in DMF (6 mL), and 2N aqueous hydrochloric acid (70 mL) was added. The precipitate was collected by filtration and dried at 105 °C overnight to afford isoflavone **23** (1.943 g, 93%) as a slightly pinkish solid. Instead of the aqueous workup, the solvents can be removed in vacuo after addition of methanol, and the product can be precipitated from DMF with 2N aqueous hydrochloric acid, too.

mp: 282–284 °C (lit.[10] 284 °C); TLC (isohexane:EtOAc 2:3 *v/v*): R_f = 0.25; ^{1}H NMR (300 MHz, DMSO-d_6): δ 10.75 (s, 1H), 9.32 (s, 1H), 9.23 (s, 1H), 8.14 (s, 1H), 7.93 (d, *J* = 8.7 Hz, 1H), 6.97 (d, *J* = 8.3 Hz, 1H), 6.93 (dd, *J* = 8.8 Hz, *J* = 2.2 Hz, 1H), 6.86 (d, *J* = 2.3 Hz, 1H), 6.35 (d, *J* = 2.3 Hz, 1H), 6.26 (dd, *J* = 8.3 Hz, *J* = 2.3 Hz, 1H) ppm; ^{13}C NMR (75 MHz, DMSO-d_6): δ 175.15 (C_q), 162.43 (C_q), 158.36 (C_q), 157.43 (C_q), 156.37 (C_q), 154.32 (CH), 132.08 (CH), 127.20 (CH), 121.78 (C_q), 116.57 (C_q), 115.04 (CH), 110.11 (C_q), 106.26 (CH), 102.84 (CH), 102.05 (CH) ppm; IR (ATR): 3416, 3060, 1613, 1381, 1268, 1160, 1121, 846, 796, 629 cm^{-1}; MS (ESI): m/z = 271.0 $[M+H]^+$; analysis (calcd., found for $C_{15}H_{10}O_5$): C (66.67, 66.37), H (3.73, 3.79).

Supplementary Note 17

Synthesis of 1 and 2: Preparation of ATH-Precursor 13 by Chemoselective Propargylation

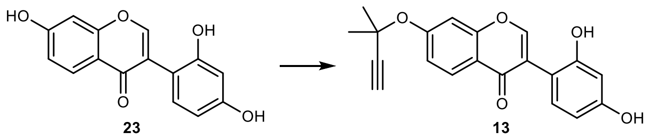

Isoflavone **23** (405.4 mg, 1.50 mmol), copper(I) iodide (28.6 mg, 0.15 mmol, 0.1 equiv.), potassium carbonate (228.0 mg, 1.65 mmol, 1.1 equiv.) and potassium iodide (298.8 mg, 1.8 mmol, 1.2 equiv.) were suspended in DMF (5.0 mL). 3-Chloro-3-methylbut-1-yne (0.20 mL, 1.8 mmol, 1.2 equiv.) was added dropwise, and the resulting reaction mixture was stirred for 17.5 h at room temperature. The reaction was quenched by addition of 1 N aqueous hydrochloric acid. The mixture was extracted three times with EtOAc, the combined organic layers were dried over $MgSO_4$, and the solvents were removed in vacuo. The residue was purified by flash chromatography (EtOAc:isohexane 1:1 *v/v*) to afford isoflavone **13** (419.1 mg, 83%) as a white to yellowish solid.

mp: 152–154 °C; TLC (isohexane:EtOAc 1:1 *v/v*): R_f = 0.43; ^{1}H NMR (300 MHz, acetone-d_6): δ 9.02 (s, 1H), 8.45 (s, 1H), 8.33 (s, 1H), 8.18 (d, *J* = 8.9 Hz, 1H), 7.51 (d, *J* = 2.3 Hz, 1H), 7.30 (dd, *J* = 9.0 Hz, *J* = 2.4 Hz, 1H), 7.16 (d, *J* = 8.1 Hz, 1H), 6.42–6.50 (m, 2H), 3.41 (s, 1H), 1.78 (s, 6H) ppm; ^{13}C NMR (75 MHz, acetone-d_6): δ 178.70 (C_q), 161.83 (C_q), 160.46 (C_q), 158.69 (C_q), 158.04 (C_q), 156.53 (CH), 132.30 (CH), 127.87 (CH), 124.84 (C_q), 119.85 (CH), 118.91 (C_q), 112.60 (C_q), 108.70 (CH), 106.89 (CH), 105.83 (CH), 85.39 (C_q), 77.57 (CH), 74.09 (C_q), 29.83 (CH_3) ppm; IR (ATR): 3262, 1917, 1592, 1433, 1116, 883, 698 cm^{-1}; MS (ESI): m/z = 337.2 $[M+H]^+$; analysis (calcd., found for $C_{20}H_{16}O_5$): C (71.42, 71.43), H (4.80, 4.65).

Supplementary Note 18

Synthesis of 1 and 2: Preparation of Isoflavone 13 *via* Suzuki Coupling

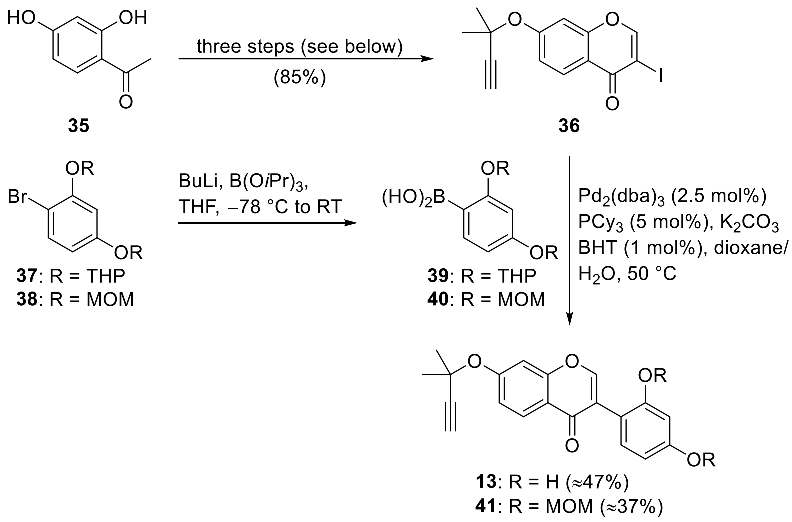

Iodochromone **36** was synthesized over three steps from acetophenone **35** in a procedure adapted from Gammill,[11] and the boronic acids **39** and **40** were obtained from the reported 4-bromoresorcinol derivates **37** and **38**.[12,13] Unfortunately, purification of the products **13** (after acidic work-up) and **41** of the Suzuki coupling proved to be very difficult, and the yields were poor. Therefore, deprotection of **41** was not even attempted.

Improved Procedure for the Propargylation of Acetophenone 35

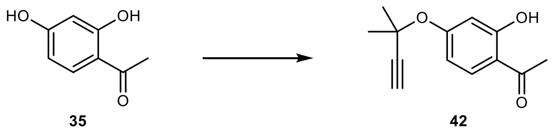

A 100 mL round-bottomed flask was charged with 2’,4’-dihydroxyacetophenone (**35**, 2.844 g, 18.7 mmol), copper(I) iodide (356.0 mg, 1.87 mmol, 0.1 equiv.), potassium carbonate (7.492 g, 54.2 mmol, 2.9 equiv.) and potassium iodide (9.309 g, 56.1 mmol, 3.0 equiv.) and acetone (62 mL) was added. 3-Chloro-3-methylbut-1-yne (4.4 mL, 39.3 mmol, 2.1 equiv.) was added dropwise at room temperature. The resulting mixture was stirred for 1 h at room temperature.

The reaction was quenched by addition of 2N aqueous hydrochloric acid. The mixture was extracted three times with EtOAc, the combined organic layers were dried over $MgSO_4$, and the solvents were removed in vacuo. The residue was purified by flash chromatography (iso-hexane:EtOAc 4:1 *v/v*) to afford acetophenone **42** (3.916 g, 96%) as a white solid.

mp: 59–61 °C; TLC (isohexane:EtOAc 4:1 *v/v*): R_f = 0.57; ^{1}H NMR (300 MHz, $CDCl_3$): δ 12.61 (s, 1H), 7.64 (d, *J* = 8.9 Hz, 1H), 6.90 (d, *J* = 2.5 Hz, 1H), 6.67 (dd, *J* = 8.9 Hz, *J* = 2.5 Hz, 1H), 2.67 (s, 1H), 2.57 (s, 3H), 1.73 (s, 6H) ppm; ^{13}C NMR (126 MHz, $CDCl_3$): δ 202.75 (C_q), 164.32 (C_q), 162.59 (C_q), 131.78 (CH), 114.47 (C_q), 111.01 (CH), 106.44 (CH), 84.67 (C_q), 75.07 (CH), 72.32 (C_q), 29.52 (CH_3), 26.26 (CH_3) ppm; IR (ATR): 3262, 1611, 1365, 1235, 794, 717 cm^{-1}; MS (ESI): m/z = 219.1 $[M+H]^+$; analysis (calcd., found for $C_{13}H_{14}O_3$): C (71.54, 71.25), H (6.47, 6.42).

Preparation of Enamine 43

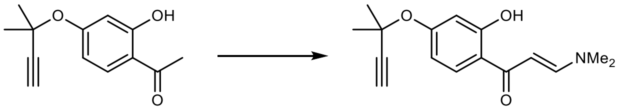

In a 10 mL round-bottomed flask acetophenone **42** (1.772 g, 8.12 mmol) was dissolved in *N,N*-dimethylformamide dimethyl acetal (4.3 mL, 32.5 mmol, 4.0 equiv.) and heated to 65 °C for 2.5 h and afterwards to 75 °C for 30 min. The reaction mixture was cooled to room temperature and poured onto water. The aqueous layer was extracted once with EtOAc and twice with DCM, the combined organic layers were dried over $MgSO_4$, and the solvents were removed in vacuo. The residue was purified by flash chromatography (EtOAc:isohexane 2:1 *v/v*) to afford enamine **43** (2.003 g, 90%) as a bright yellow solid.

mp: 133–134 °C; TLC (EtOAc:isohexane 2:1 *v/v*): R_f = 0.46; ^{1}H NMR (500 MHz, $CDCl_3$): δ 14.29 (s, 1H), 7.85 (d, *J* = 12.0 Hz, 1H), 7.61 (d, *J* = 9.1 Hz, 1H), 6.88 (d, *J* = 2.2 Hz, 1H), 6.60

(dd, J = 8.8 Hz, J = 2.5 Hz, 1H), 5.70 (d, J = 12.3 Hz, 1H), 3.18 (br. s., 3H), 2.96 (br. s., 3H), 2.63 (s, 1H), 1.70 (s, 6H) ppm; ^{13}C NMR (126 MHz, $CDCl_3$): δ 190.74 (C_q), 164.59 (C_q), 160.65 (C_q), 154.08 (CH), 129.12 (CH), 114.87 (C_q), 110.45 (CH), 107.37 (CH), 89.92 (CH), 85.28 (C_q), 74.51 (CH), 72.03 (C_q), 45.28 (CH_3, very weak), 37.28 (CH_3, very weak), 29.57 (CH_3) ppm; IR (ATR): 3220, 1627, 1131, 1110, 843, 774 cm^{-1}; MS (ESI): m/z = 274.1 $[M+H]^+$; analysis (calcd., found for $C_{16}H_{19}NO_3$): C (70.31, 70.36), H (7.01, 7.23), N (5.21, 5.10).

Preparation of Iodide 36

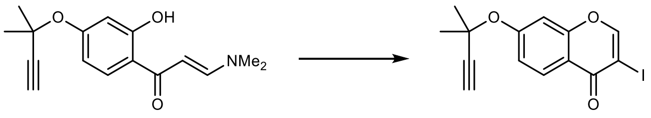

In a 50 mL round-bottomed flask enamine **43** (2.000 g, 7.32 mmol) was dissolved in chloroform (14.6 mL), and pyridine (0.89 mL, 11.0 mmol, 1.5 equiv.) and iodine (3.714 g, 14.6 mmol, 2.0 equiv.) were added successively. The reaction mixture was stirred for 1 h at room temperature, and then quenched with saturated aqueous sodium thiosulfate solution (23 mL). The mixture was stirred for 5 min and diluted with water. The aqueous layer was extracted three times with DCM, the combined organic layers were dried over $MgSO_4$, and the solvents were removed in vacuo. The residue was purified by flash chromatography (isohexane:EtOAc 4:1 *v/v*) to afford iodide **36** (2.583 g, 98%) as a yellowish solid.

mp: 86–105 °C; TLC (isohexane:EtOAc 4:1 *v/v*): R_f = 0.42; ^{1}H NMR (500 MHz, $CDCl_3$): δ 8.23 (s, 1H), 8.14 (d, J = 9.1 Hz, 1H), 7.35 (d, J = 2.5 Hz, 1H), 7.20 (dd, = 9.0 Hz, J = 2.4 Hz, 1H), 2.70 (s, 1H), 1.75 (s, 6H) ppm; ^{13}C NMR (126 MHz, $CDCl_3$): δ 172.69 (C_q), 160.62 (C_q), 157.31 (CH), 157.17 (C_q), 127.53 (CH), 118.94 (CH), 116.31 (C_q), 105.92 (CH), 87.01 (C_q), 84.43 (C_q), 75.48 (CH), 72.91 (C_q), 29.43 (CH_3) ppm; IR (ATR): 3246, 1613, 1134, 1059, 863,

683 cm^{-1}; MS (ESI): m/z = 355.0 $[M+H]^+$; analysis (calcd., found for $C_{14}H_{11}IO_3$): C (47.48, 47.32), H (3.13, 3.12).

Preparation of THP Protected 4-Bromoresorcinol 37

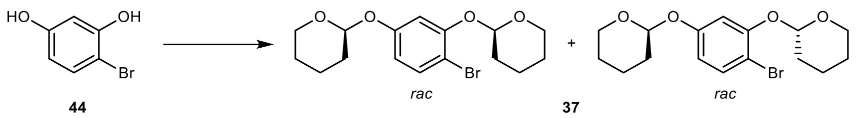

In a 25 mL round-bottomed flask 4-bromoresorcinol (**44**, 1.674 g, 8.59 mmol) was dissolved in DCM (8.6 mL) and 3,4-dihydro-2*H*-pyran (4.7 mL, 51.6 mmol, 6.0 equiv.) and pyridinium *p*-toluenesulfonate (215.9 mg, 0.86 mmol, 0.1 equiv.) was added. The reaction mixture was stirred for 85 min at room temperature and subsequently poured onto 2 N aqueous sodium hydroxide. The aqueous layer was extracted three times with DCM, the combined organic layers were dried over $MgSO_4$, and the solvents were removed in vacuo. The residue was purified by flash chromatography (isohexane:EtOAc 4:1 *v/v*) to afford bromobenzene **37** (2.995 g, 98%) as a white solid (diastereomeric mixture 1:1).

mp: 60–61 °C (lit.[12] 68–69 °C); TLC (isohexane:EtOAc 4:1 *v/v*): R_f = 0.54; ^{1}H NMR (600 MHz, $CDCl_3$): δ 7.40 (d, *J* = 8.7 Hz, 1H), 7.39 (d, *J* = 8.8 Hz, 1H), 6.90 (d, *J* = 2.6 Hz, 1H), 6.89 (d, *J* = 2.6 Hz, 1H), 6.64 (dd, *J* = 8.7 Hz, *J* = 2.6 Hz, 1H), 6.62 (dd, *J* = 8.8 Hz, *J* = 2.5 Hz, 1H), 5.51 (t, *J* = 2.9 Hz, 1H), 5.49 (t, *J* = 2.9 Hz, 1H), 5.40 (t, *J* = 3.2 Hz, 1H), 5.36 (t, *J* = 3.2 Hz, 1H), 3.85–3.96 (m, 4H), 3.57–3.66 (m, 4H), 2.06–2.15 (m, 2H), 1.93–2.03 (m, 4H), 1.81–1.90 (m, 6H), 1.56–1.77 (m, 12H) ppm; ^{13}C NMR (151 MHz, $CDCl_3$): δ 157.45 (C_q), 157.31 (C_q), 153.99 (C_q), 153.84 (C_q), 132.91 (CH), 110.61 (CH), 110.42 (CH), 106.24 (CH), 105.70 (CH), 104.80 (C_q), 104.64 (C_q), 96.82 (CH), 96.72 (CH), 96.56 (CH), 96.36 (CH), 61.99 (CH_2), 61.91 (CH_2), 61.78 (CH_2), 61.74 (CH_2), 30.24 (CH_2), 30.19 (CH_2), 30.14 (CH_2), 30.11 (CH_2), 25.20 (CH_2), 25.11 (CH_2), 18.64 (CH_2), 18.56 (CH_2), 18.27 (CH_2), 18.24 (CH_2) ppm; IR (ATR): 2939,

1104, 992, 871 cm^{-1}; MS (ESI): m/z = 379.1 $[M+H]^{+}$; analysis (calcd., found for $C_{16}H_{21}BrO_4$): C (53.79, 54.06), H (5.93, 6.06).

Suzuki Coupling of Iodide 36 and the THP Protected Boronic Acid 39

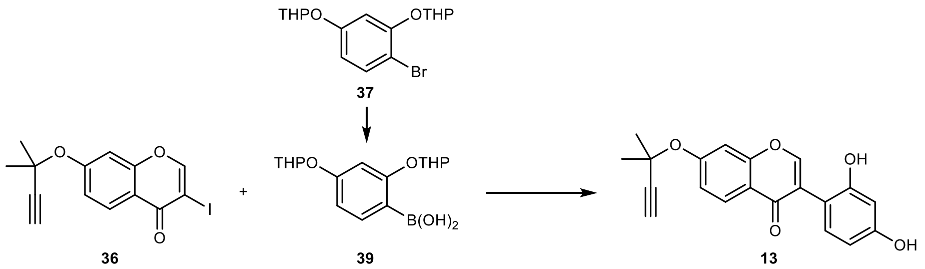

Bromobenzene **37** (964.6 mg, 2.70 mmol, 1.8 equiv.) was dissolved in THF (5.4 mL) and triisopropyl borate (1.25 mL, 5.40 mmol, 3.6 equiv.) in a 25 mL round-bottomed flask and cooled to –78 °C. *n*-Butyllithium solution (3.0 mL, 1.6 M in hexanes, 4.86 mmol, 3.2 equiv.) was added over 15 min, and the reaction mixture was stirred for 60 min at –78 °C. The dry-ice/acetone bath was removed, and the reaction mixture was stirred for 2 h and then poured onto saturated aqueous NH_4Cl solution. The aqueous layer was extracted three times with EtOAc, the combined organic layers were dried over $MgSO_4$, and the solvents were removed in vacuo. To the crude boronic acid was added iodide **36** (531.2 mg, 1.50 mmol, 1.0 equiv.), potassium carbonate (622.0 mg, 4.50 mmol, 3.0 equiv.), BHT (3.3 mg, 15.0 µmol, 1 mol%), 1,4-dioxane (6.2 mL) and water (2.6 mL). The mixture was purged with argon for 5 min. Tricyclohexylphosphine (21.0 mg, 75.0 µmol, 5 mol%) and $Pd_2(dba)_3$ (34.3 mg, 37.5 µmol, 2.5 mol%) were added successively, and the reaction mixture was stirred at 50 °C for 65 min. The reaction mixture was diluted with THF (6.2 mL), and concentrated hydrochloric acid (1.5 mL) was added carefully. After stirring at 50 °C for 25 min, the reaction mixture was cooled to room temperature, and water was added. The aqueous layer was extracted three times with EtOAc, the combined organic layers were dried over $MgSO_4$, and the solvents were removed in vacuo. The residue

was purified by repeated flash chromatography (isohexane:EtOAc 1st: 1.1; 2nd: 3:2 *v/v*) to afford isoflavone **13** (237.3 mg, ≈47%, impure) as a brown solid.

For analytical details see Supplementary Note 17

Suzuki Coupling of Iodide 36 and the MOM Protected Boronic Acid 40

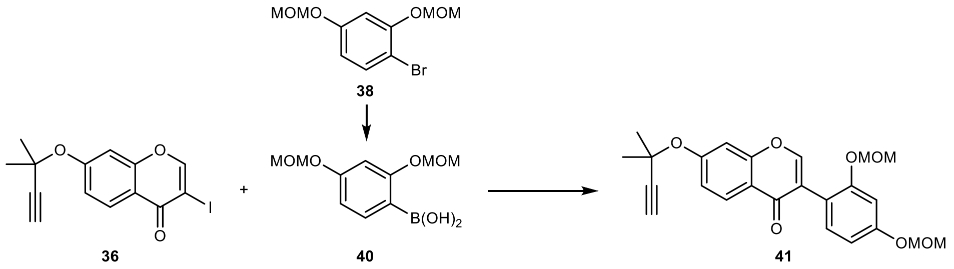

Bromobenzene **38**[13] (700.0 mg, 2.52 mmol, 1.8 equiv.) was dissolved in THF (5.1 mL) and triisopropyl borate (1.2 mL, 5.05 mmol, 3.6 equiv.) in a 10 mL round-bottomed flask and cooled to –78 °C. *n*-Butyllithium solution (2.2 mL, 1.6 M in hexanes, 3.54 mmol, 2.5 equiv.) was added over 30 min, and the reaction mixture was stirred for 15 min at –78 °C. The dry-ice/acetone bath was removed, and the reaction mixture was stirred for 3.3 h and then poured onto saturated 1 N aqueous hydrochloric acid. The aqueous layer was extracted three times with EtOAc, the combined organic layers were dried over $MgSO_4$, and the solvents were removed in vacuo. To the crude boronic acid was added iodide **36** (497.0 mg, 1.40 mmol, 1.0 equiv.), potassium carbonate (581.9 mg, 4.21 mmol, 3.0 equiv.), BHT (3.1 mg, 14.0 µmol, 1 mol%), 1,4-dioxane (5.8 mL) and water (2.5 mL). The mixture was purged with argon for 5 min. Tricyclohexylphosphine (19.7 mg, 70.2 µmol, 5 mol%) and $Pd_2(dba)_3$ (32.1 mg, 35.1 µmol, 2.5 mol%) were added successively, and the reaction mixture was stirred at 50 °C for 45 min. The reaction mixture was cooled to room temperature, and saturated aqueous NH_4Cl solution was added. The aqueous layer was extracted three times with EtOAc, the combined organic layers were dried over $MgSO_4$, and the solvents were removed in vacuo. The residue was purified by repeated flash chromatography (isohexane:EtOAc 1st: 1.1; 2nd: 2:1 *v/v*) to afford isoflavone **41**

(223.8 mg, ≈37%, impure) as a yellow gum. An analytical sample was obtained by preparative HPLC.

TLC (isohexane:EtOAc 2:1 *v/v*): R_f = 0.33; ^{1}H NMR (300 MHz, $CDCl_3$): δ 8.18 (d, *J* = 8.9 Hz, 1H), 7.90 (s, 1H), 7.38 (d, *J* = 2.3 Hz, 1H), 7.23 (d, *J* = 8.3 Hz, 1H), 7.18 (dd, *J* = 8.9 Hz, *J* = 2.3 Hz, 1H), 6.93 (d, *J* = 2.5 Hz, 1H), 6.78 (dd, *J* = 8.4 Hz, *J* = 2.4 Hz, 1H), 5.20 (s, 2H), 5.13 (s, 2H), 3.50 (s, 3H), 3.43 (s, 3H), 2.71 (s, 1H), 1.75 (s, 6H) ppm; ^{13}C NMR (75 MHz, $CDCl_3$): δ 175.65 (C_q), 160.08 (C_q), 158.51 (C_q), 157.19 (C_q), 156.30 (C_q), 153.68 (CH), 132.09 (CH), 127.17 (CH), 122.41 (C_q), 119.16 (C_q), 118.31 (CH), 115.45 (C_q), 108.95 (CH), 106.47 (CH), 104.18 (CH), 95.02 (CH_2), 94.43 (CH_2), 84.74 (C_q), 75.20 (CH), 72.76 (C_q), 56.13 (CH_3), 56.02 (CH_3), 29.45 (CH_3) ppm; IR (ATR): 1608, 1437, 1128, 997 cm^{-1}; MS (ESI): m/z = 425.3 $[M+H]^+$; analysis (calcd., found for $C_{24}H_{24}O_7$): C (67.91, 67.76), H (5.70, 5.52).

Supplementary Note 19

Synthesis of 1 and 2: Preparation of Pterocarpan 24 by ATH Cascade and Cyclisation

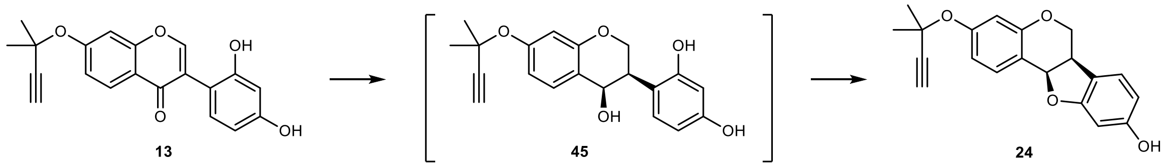

Preparation of the catalyst solution:

$[Ru(p\text{-cym})Cl_2]_2$ (3.7 mg, 6.0 µmol) and (*R*,*R*)-TsDPEN (5.0 mg, 13.3 µmol) were dissolved in EtOAc (0.36 mL) in a 5 mL round-bottomed flask. Another flask containing triethylamine (1.5 mL) was cooled to 0 °C, and formic acid (0.5 mL) was added. The mixture was stirred vigorously for 5 min at room temperature. An aliquot of this mixture (0.94 mL) was added to the ruthenium catalyst, and the resulting solution was stirred for another 5 min.

Reaction:

In a 5 mL round-bottomed flask isoflavone **13** (146.6 mg, 0.44 mmol, 1.0 equiv.) was suspended in EtOAc (0.54 mL) at 45 °C, and an aliquot of the catalyst solution (0.47 mL, ca. 1 mol% catalyst) was added. The reaction mixture was stirred at 45 °C for 19 h, cooled to room temperature and diluted with ethanol (4.4 mL). Hydrochloric acid (37%, 0.11 mL, ca. 3.0 equiv.) was added, the mixture was stirred for 13 min, and another portion of hydrochloric acid (37%, 0.11 mL, ca. 3.0 equiv.) was added. After 7 min, the reaction was quenched by addition of saturated aqueous NH_4Cl solution, and the mixture was extracted three times with EtOAc. The combined organic layers were dried over $MgSO_4$, the solvents were removed in vacuo, and the residue was purified by flash chromatography (DCM:EtOAc 2:1 *v/v*) to afford pterocarpan **24** (115.2 mg, 82%, >99% *ee*) as an off-white solid foam.

Pterocarpan **24**:

mp: 27–45 °C; TLC (DCM:EtOAc 2:1 *v/v*): R_f = 0.86; $[\alpha]_D^{25}$ = –156.2 (*c* 0.97 $CHCl_3$); ^{1}H NMR (500 MHz, $CDCl_3$): δ 7.43 (d, *J* = 8.5 Hz, 1H), 7.09 (d, *J* = 8.8 Hz, 1H), 6.91 (dd, *J* = 8.4 Hz, *J* = 2.4 Hz, 1H), 6.89 (d, *J* = 2.2 Hz, 1H), 6.35–6.41 (m, 2H), 5.52 (d, *J* = 6.9 Hz, 1H), 5.15 (br. s., 1H), 4.25 (dd, *J* = 11.0 Hz, *J* = 4.7 Hz, 1H), 3.60–3.66 (m, 1H), 3.53 (ddd, *J* = 11.3 Hz, *J* = 6.6 Hz, *J* = 4.7 Hz, 1H), 2.60 (s, 1H), 1.67 (s, 3H), 1.66 (s, 3H) ppm; ^{13}C NMR (151 MHz, $CDCl_3$): δ 160.65 (C_q), 156.99 (C_q), 156.89 (C_q), 155.86 (C_q), 131.21 (CH), 124.98 (CH), 119.27 (C_q), 115.09 (CH), 114.14 (C_q), 109.06 (CH), 107.66 (CH), 98.36 (CH), 85.71 (C_q), 78.60 (CH), 74.17 (CH), 72.40 (C_q), 66.56 (CH_2), 39.41 (CH), 29.54 (CH_3), 29.49 (CH_3) ppm; IR (ATR): 3394, 3281, 1494, 1104, 1080, 957 cm^{-1}; GC-MS (EI): m/z = 322.3 $[M]^+$; analysis (calcd., found for $C_{20}H_{18}O_4$): C (74.52, 74.22), H (5.63, 6.01).

The isoflavan-4-ol (+)-**45** may be isolated, too, without the addition of ethanol and hydrochloric acid by flash chromatography (DCM:EtOAc 2:1 *v/v*) as a white solid in 70% yield.

Isoflavan-4-ol **45**:

mp: 145–147 °C; TLC (DCM:EtOAc 2:1 *v/v*): R_f = 0.30; $[\alpha]_D^{25}$ = +134.8 (*c* 0.58 acetone); ^{1}H NMR (300 MHz, acetone-d_6): δ 8.70 (s, 1H), 8.11 (s, 1H), 7.12–7.20 (m, 1H), 7.02 (d, *J* = 8.5 Hz, 1H), 6.68–6.76 (m, 2H), 6.41 (d, *J* = 2.5 Hz, 1H), 6.32 (dd, *J* = 8.3 Hz, *J* = 2.5 Hz, 1H), 4.79–4.88 (m, 1H), 4.62 (dd, *J* = 12.1 Hz, *J* = 10.2 Hz, 1H), 4.57 (d, *J* = 4.3 Hz, 1H), 4.13 (ddd, *J* = 10.3 Hz, *J* = 3.7 Hz, *J* = 1.3 Hz, 1H), 3.47 (dt, *J* = 12.3 Hz, *J* = 3.2 Hz, 1H), 3.17 (s, 1H), 1.62 (s, 6H) ppm; ^{13}C NMR (75 MHz, acetone-d_6): δ 158.33 (C_q), 157.58 (C_q), 157.28 (C_q), 155.53 (C_q), 131.69 (CH), 131.32 (CH), 121.28 (C_q), 117.05 (C_q), 114.17 (CH), 109.39 (CH), 107.65 (CH), 104.05 (CH), 86.99 (C_q), 75.74 (CH), 72.92 (C_q), 66.73 (CH), 65.00 (CH_2), 40.23 (CH), 30.07 (CH_3), 30.01 (CH_3) ppm; IR (ATR): 3379, 3270, 1112, 1031, 878, 657 cm^{-1}; HRMS (m/z): $[M–OH]^+$ calcd. for $C_{20}H_{19}O_4$, 323.1278; found, 323.1273.

Supplementary Note 20

Synthesis of 1 and 2: Preparation of Pterocarpan 12

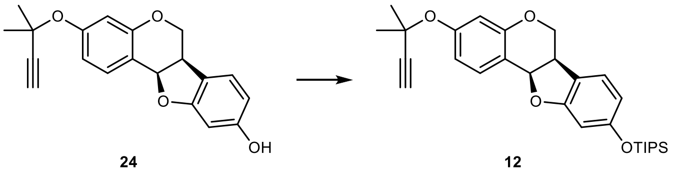

To a solution of pterocarpan **24** (1.291 g, 4.02 mmol, >99% *ee*) in DCM (8.0 mL) DMAP (48.9 mg, 0.40 mmol, 0.1 equiv.), triethylamine (1.7 mL, 12.0 mmol, 3.0 equiv.) and TIPSCl (2.6 mL, 12.0 mmol, 3.0 equiv.) were added subsequently. The reaction was stirred for 19 h at room temperature and quenched by addition of saturated aqueous NH_4Cl solution. The mixture was extracted three times with DCM, the combined organic layers were dried over $MgSO_4$, the solvents were removed in vacuo, and the residue was purified by flash chromatography (DCM:pentane 2:1 *v/v*) to afford pterocarpan **12** (1.863 g, 97%, >99% *ee*) as a colourless oil.

TLC (DCM:pentane 2:1 *v/v*): R_f = 0.68; $[\alpha]_D^{25}$ = –119.5 (*c* 0.45 $CHCl_3$); ^1H NMR (500 MHz, $CDCl_3$): δ 7.43 (d, *J* = 8.5 Hz, 1H), 7.07 (d, *J* = 7.6 Hz, 1H), 6.92 (dd, *J* = 8.4 Hz, *J* = 2.4 Hz, 1H), 6.89 (d, *J* = 2.5 Hz, 1H), 6.42–6.47 (m, 2H), 5.50 (d, *J* = 6.6 Hz, 1H), 4.26 (dd, *J* = 10.4 Hz, *J* = 4.7 Hz, 1H), 3.59 (t, *J* = 11.0 Hz, 1H), 3.52 (ddd, *J* = 11.3 Hz, *J* = 6.5 Hz, *J* = 4.9 Hz, 1H), 2.60 (s, 1H), 1.67 (s, 3H), 1.67 (s, 3H), 1.20–1.30 (m, 3H), 1.07–1.13 (m, 18H) ppm; ^{13}C NMR (126 MHz, $CDCl_3$): δ 160.38 (C_q), 157.33 (C_q), 156.99 (C_q), 155.87 (C_q), 131.16 (CH), 124.55 (CH), 119.44 (C_q), 115.08 (CH), 114.25 (C_q), 112.48 (CH), 109.12 (CH), 102.65 (CH), 85.76 (C_q), 78.38 (CH), 74.10 (CH), 72.36 (C_q), 66.65 (CH_2), 39.51 (CH), 29.56 (CH_3), 29.51 (CH_3), 17.88 (CH_3), 12.60 (CH) ppm; IR (ATR): 3289, 1492, 1145, 1106, 969, 880, 636 cm^{-1}; MS (ESI): m/z = 479.4 $[M+H]^+$; analysis (calcd., found for $C_{29}H_{38}O_4Si$): C (72.76, 72.88), H (8.00, 8.28).

Supplementary Note 21

Synthesis of 1 and 2: Preparation of 6a-Hydroxypterocarpan 11 and Isoflavanone 25

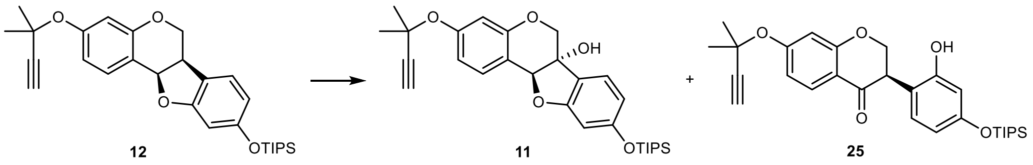

Pterocarpan **12** (125.9 mg, 0.26 mmol, >99% *ee*), *N*-hydroxyphthalimide (42.9 mg, 0.26 mmol, 1.0 equiv.) and cobalt(II) acetate (23.3 mg, 0.13 mmol, 0.5 equiv.) were dissolved in MeCN (5.3 mL) in a 10 mL round-bottomed flask, and 1,1,1,3,3,3-hexafluoropropan-2-ol (0.08 mL, 0.79 mmol, 3.0 equiv.) was added. The argon atmosphere was replaced with oxygen (balloon), and the reaction mixture was stirred for 1 h at room temperature. The oxygen atmosphere was replaced with argon, and triphenylphosphine (69.0 mg, 0.26 mmol, 1.0 equiv.) was added. After additional stirring for 45 min, the reaction was quenched by addition of saturated aqueous

NH_4Cl solution. The mixture was extracted three times with EtOAc, the combined organic layers were dried over $MgSO_4$, the solvents were removed in vacuo, and the residue was purified by flash chromatography (DCM:EtOAc 30:1 *v/v*) to afford 6a-hydroxypterocarpan **11** (72.2 mg, 55%, >99% *ee*) as a brown gum and isoflavanone **25** (24.6 mg, 19%, 92% *ee*) as a yellowish solid.

6a-Hydroxypterocarpan **11**:

TLC (DCM:EtOAc 30:1 *v/v*): R_f = 0.45; $[\alpha]_D^{25}$ = –137.0 (*c* 0.36 $CHCl_3$); ^1H NMR (500 MHz, $CDCl_3$): δ 7.40 (d, *J* = 8.5 Hz, 1 H), 7.18 (d, *J* = 8.2 Hz, 1H), 6.93 (dd, *J* = 8.5 Hz, *J* = 2.5 Hz, 1H), 6.90 (d, *J* = 2.5 Hz, 1H), 6.52 (dd, *J* = 8.0 Hz, *J* = 2.0 Hz, 1H), 6.41 (d, *J* = 1.9 Hz, 1H), 5.25 (s, 1H), 4.23 (dd, *J* = 11.7 Hz, *J* = 0.9 Hz, 1H), 3.91 (d, *J* = 11.7 Hz, 1H), 2.60 (br. s, 2H), 1.66 (s, 6H), 1.20–1.31 (m, 3H), 1.07–1.13 (m, 18H) ppm; ^{13}C NMR (126 MHz, $CDCl_3$): δ 160.45 (C_q), 158.78 (C_q), 157.04 (C_q), 155.03 (C_q), 131.25 (CH), 123.60 (CH), 120.49 (C_q), 115.55 (CH), 113.94 (C_q), 113.33 (CH), 108.95 (CH), 103.02 (CH), 85.67 (C_q), 84.25 (CH), 76.79 (C_q), 74.21 (CH), 72.33 (C_q), 70.06 (CH_2), 29.52 (CH_3), 29.50 (CH_3), 17.86 (CH_3), 12.59 (CH) ppm; IR (ATR): 1493, 1123, 970 cm^{-1}; MS (ESI): m/z = 495.4 $[M+H]^+$; analysis (calcd., found for $C_{29}H_{38}O_5Si$): C (70.41, 70.27), H (7.74, 8.02).

Isoflavanone **25**:

mp: 34–36 °C; TLC (DCM:EtOAc 30:1 *v/v*): R_f = 0.54; $[\alpha]_D^{25}$ = +254.8 (*c* 1.18 $CHCl_3$); ^1H NMR (300 MHz, $CDCl_3$): δ 8.24 (s, 1H), 7.84 (d, *J* = 8.9 Hz, 1H), 7.35 (d, *J* = 8.5 Hz, 1H), 6.91 (d, *J* = 2.5 Hz, 1H), 6.82 (dd, *J* = 8.9 Hz, *J* = 2.3 Hz, 1H), 6.52 (d, *J* = 2.5 Hz, 1H), 6.44 (dd, *J* = 8.4 Hz, *J* = 2.5 Hz, 1H), 4.96 (dd, *J* = 11.9 Hz, 2.8 Hz, 1H), 4.81 (dd, *J* = 11.9 Hz, *J* = 4.5 Hz, 1H), 3.93 (t, *J* = 3.8 Hz, 1H), 2.69 (s, 1H), 1.73 (s, 6H), 1.17–1.32 (m, 3H), 1.04–1.13 (m, 18H) ppm; ^{13}C NMR (75 MHz, $CDCl_3$): δ 193.20 (C_q), 163.50 (C_q), 163.12 (C_q), 157.16 (C_q), 156.41 (C_q), 129.36 (CH), 127.18 (CH), 115.47 (C_q), 114.14 (CH), 113.25 (C_q), 112.69 (CH), 109.43 (CH), 105.84 (CH), 84.54 (C_q), 75.28 (CH), 72.52 (C_q), 70.00 (CH_2), 45.64 (CH),

29.65 (CH_3), 29.37 (CH_3), 17.91 (CH_3), 12.63 (CH) ppm; IR (ATR): 3291, 1600, 1243, 1123, 1104, 989, 847, 634 cm^{-1}; MS (ESI): m/z = 495.6 $[M+H]^+$; analysis (calcd., found for $C_{29}H_{38}O_5Si$): C (70.41, 70.30), H (7.74, 7.59).

Supplementary Note 22

Synthesis of 1 and 2: Preparation of 26 and 27 *via* Thermal Pyran Ring Annellation in DMSO

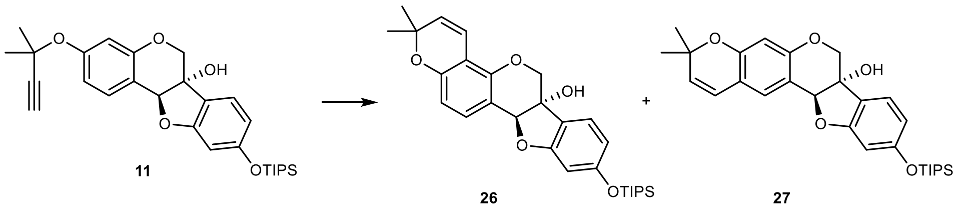

A solution of 6a-hydroxypterocarpan **11** (291.4 mg, 0.59 mmol, >99% *ee*) in DMSO (5.7 mL) was heated to 175 °C for 40 min. The reaction mixture was cooled to room temperature, diluted with EtOAc (40 mL) and washed twice with water (2x 40 mL). The organic layer was dried over $MgSO_4$, the solvents were removed in vacuo, and the residue was purified twice by flash chromatography (DCM:EtOAc 40:1 *v/v*) to afford 9-*O*-TIPS-glyceollin I (**26**, 202.3 mg, 69%, >99% *ee*) as a yellowish solid and 9-*O*-TIPS-glyceollin II (**27**, 33.8 mg, 12%, >99% *ee*, **27**/**26** ≈ 29:1) as a yellowish solid.

9-*O*-TIPS-glyceollin I (**26**):

mp: 51–54 °C; TLC (DCM:EtOAc 40:1 *v/v*): R_f = 0.48; $[\alpha]_D^{25}$ = –131.9 (*c* 0.39 $CHCl_3$); ^1H NMR (300 MHz, $CDCl_3$): δ 7.25 (d, *J* = 8.7 Hz, 1H), 7.19 (d, *J* = 8.1 Hz, 1H), 6.63 (d, *J* = 10.0 Hz, 1H), 6.56 (d, *J* = 8.5 Hz, 1H), 6.51 (dd, *J* = 8.1 Hz, *J* = 2.1 Hz, 1H), 6.40 (d, *J* = 2.1 Hz, 1H), 5.58 (d, *J* = 10.0 Hz, 1H), 5.24 (s, 1H), 4.25 (dd, *J* = 11.5 Hz, *J* = 0.9 Hz, 1H), 3.93 (d, *J* = 11.5 Hz, 1H), 2.45 (s, 1H), 1.43 (s, 3H), 1.42 (s, 3H), 1.17–1.33 (m, 3H), 1.02–1.16 (m, 18H) ppm; ^{13}C NMR (75 MHz, $CDCl_3$): δ 160.54 (C_q), 158.81 (C_q), 154.13 (C_q), 150.35 (C_q), 130.80

(CH), 129.28 (CH), 123.57 (CH), 120.49 (C_q), 116.46 (CH), 113.28 (CH), 112.00 (C_q), 111.13 (CH), 110.29 (C_q), 103.02 (CH), 84.66 (CH), 76.73 (C_q), 76.21 (C_q), 70.10 (CH_2), 27.84 (CH_3), 27.79 (CH_3), 17.88 (CH_3), 12.60 (CH) ppm; IR (ATR): 3374, 1150, 1116, 1082, 970, 682 cm^{-1}; MS (ESI): m/z = 495.6 $[M+H]^+$; analysis (calcd., found for $C_{29}H_{38}O_5Si$): C (70.41, 70.45), H (7.74, 8.01).

<u>9-*O*-TIPS-glyceollin II (**27**):</u>

mp: 49–51 °C; TLC (DCM:EtOAc 40:1 *v/v*): R_f = 0.39; $[\alpha]_D^{25}$ = –152.0 (*c* 0.15 $CHCl_3$); ^1H NMR (500 MHz, $CDCl_3$): δ 7.19 (d, *J* = 8.2 Hz, 1H), 7.12 (s, 1H), 6.51 (dd, *J* = 8.2 Hz, *J* = 2.2 Hz, 1H), 6.40 (d, *J* = 1.9 Hz, 1H), 6.39 (s, 1H), 6.32 (d, *J* = 10.1 Hz, 1H), 5.56 (d, *J* = 9.8 Hz, 1H), 5.24 (s, 1H), 4.21 (dd, *J* = 11.7 Hz, *J* = 0.9 Hz, 1H), 3.92 (d, *J* = 11.7 Hz, 1H), 2.42 (s, 1H), 1.44 (s, 3H), 1.41 (s, 3H), 1.20–1.29 (m, 3H), 1.07–1.12 (m, 18H) ppm; ^{13}C (125 MHz, $CDCl_3$): δ 160.47 (C_q), 158.82 (C_q), 155.43 (C_q), 154.61 (C_q), 129.35 (CH), 128.34 (CH), 123.59 (CH), 121.53 (CH), 120.44 (C_q), 116.92 (C_q), 113.35 (CH), 112.08 (C_q), 104.81 (CH), 103.01 (CH), 84.41 (CH), 76.63 (C_q), 69.99 (CH_2), 28.14 (CH_3), 28.00 (CH_3), 17.87 (CH_3), 12.61 (CH) ppm; IR (ATR): 3405, 1492, 1124, 970, 682 cm^{-1}; MS (ESI): m/z = 495.4 $[M+H]^+$; analysis (calcd., found for $C_{29}H_{38}O_5Si$): C (70.41, 70.75), H (7.74, 7.74).

Supplementary Note 23

Synthesis of 1 and 2: Preparation of 26 and 27 *via* Thermal Pyran Ring Annellation in DMF

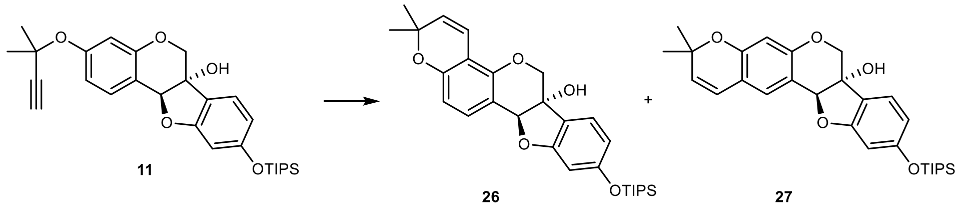

A 20 mL sealed tube was charged with 6a-hydroxypterocarpan **11** (204.8 mg, 0.41 mmol, >99% *ee*) in DMF (4.1 mL) and heated to 175 °C for 1 h. The reaction was cooled to room temperature, water was added, and the resulting mixture was extracted three times with EtOAc. The combined organic layers were dried over $MgSO_4$, the solvents were removed in vacuo, and the residue was subjected twice to flash chromatography (DCM:EtOAc 40:1 *v/v*) to afford a first fraction with 9-*O*-TIPS-glyceollin I (**26**, 145.0 mg, 70%, >99% *ee*, yellowish solid) and a second fraction (36.3 mg, 11% **27** + 7% **26**, >99% *ee*) as a yellowish solid. For complete separation repeated flash chromatography is necessary.

For analytical data, see Supplementary Note 22.

Supplementary Note 24

Synthesis of 1 and 2: Preparation of 26 and 27 *via* Gold(I)-Catalysed Hydroarylation

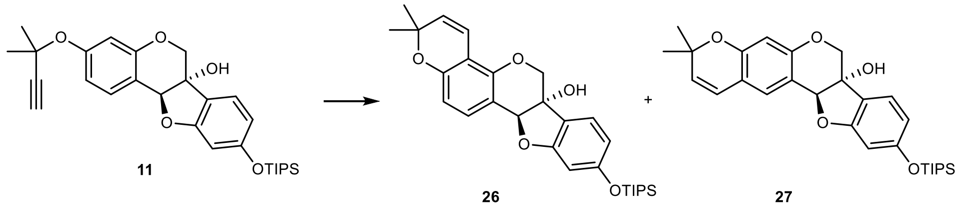

In a 10 mL round-bottomed flask 6a-hydroxypterocarpan **11** (285.9 mg, 0.58 mmol, >99% *ee*) was dissolved in dichloroethane (5.8 mL), and (2-di-*tert*-butylphosphino-3,4,5,6-tetramethyl-2′,4′,6′-triisopropylbiphenyl)gold(I) bis(trifluoromethanesulfonyl)imide (**28**, 2.8 mg, 2.9 µmol, 1 mol%) was added. The reaction was stirred for 3 h at room temperature and quenched with saturated aqueous NH_4Cl solution. The mixture was extracted three times with DCM, the combined organic layers were dried over $MgSO_4$, and the solvents were removed in vacuo. The residue was purified once by flash chromatography (DCM:EtOAc 40:1 *v/v*) to afford a first fraction with 9-*O*-TIPS-glyceollin I (**26**, 148.5 mg, 52%, >99% *ee*) as a yellowish solid and a

second fraction (101.9 mg, 13% **26** + 23% **27**, >99 % *ee*) as a yellowish solid. For separation repeated flash chromatography is necessary.

For analytical data, see Supplementary Note 22.

Supplementary Note 25

Synthesis of 1 and 2: Preparation of Glyceollin I (1)

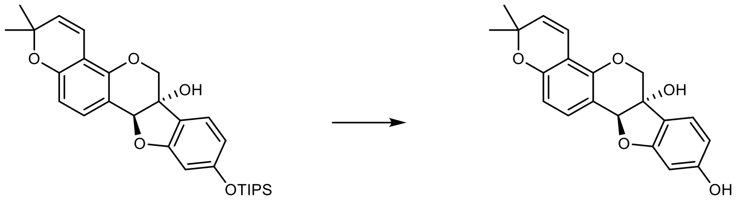

9-*O*-TIPS-Glyceollin I (**26**, 142.9 mg, 0.29 mmol, >99% *ee*) was dissolved in THF (5.0 mL) in a 10 mL round-bottomed flask and cooled to 0 °C. A solution of tetrabutylammonium fluoride (1 M in THF containing 5% water, 0.43 mL, 0.43 mmol, 1.5 equiv.) was added dropwise, and the resulting solution was stirred for 11 min at 0 °C. The reaction mixture was quenched with water at 0 °C, diluted with saturated aqueous NH_4Cl and extracted three times with Et_2O. The combined organic layers were dried over $MgSO_4$, and the solvents were removed at 25 °C in vacuo. The residue was purified by flash chromatography (Et_2O:pentane 3:1 *v/v*), the solvents were removed in vacuo, and the product was dissolved in methanol (1 mL). Water (2 mL) was added, the solvents were removed in vacuo, and the resulting white solid was dried over P_2O_5 to yield glyceollin I (**1**, 88.3 mg, 90%, >99% *ee*) as a white solid.

mp: 99–101 °C (lit.[14] 95–101 °C); TLC (Et_2O:pentane 3:1 *v/v*): R_f = 0.39; $[\alpha]_D^{20}$ = –218.0 (*c* 0.14 EtOAc) (lit.[14] $[\alpha]_D^{25}$ = –202.6 (*c* 0.15 EtOAc)); ^{1}H NMR (300 MHz, MeOD-d_4): δ 7.21 (d, *J* = 8.5 Hz, 1H), 7.17 (d, *J* = 8.1 Hz, 1H), 6.60 (d, *J* = 10.0 Hz, 1H), 6.47 (d, *J* = 8.3 Hz, 1H), 6.41 (dd, *J* = 8.1 Hz, *J* = 2.1 Hz, 1H), 6.24 (d, *J* = 2.1 Hz, 1H), 5.60 (d, *J* = 9.8 Hz, 1H), 5.17 (s, 1H), 4.17 (dd, *J* = 11.4 Hz, *J* = 0.7 Hz, 1H), 3.94 (d, *J* = 11.5 Hz, 1H), 1.38 (s, 3H), 1.36 (s,

3H) ppm; ^{13}C (75 MHz, MeOD-d_4): δ 162.27 (C_q), 161.29 (C_q), 155.39 (C_q), 151.97 (C_q), 132.35 (CH), 130.47 (CH), 125.31 (CH), 121.28 (C_q), 117.58 (CH), 114.31 (C_q), 111.70 (CH), 111.38 (C_q), 109.54 (CH), 99.13 (CH), 86.07 (CH), 77.32 (C), 77.24 (C_q), 71.25 (CH_2), 28.17 (CH_3) ppm; IR (ATR): 3360, 1609, 1115, 1081, 961 cm^{-1}; MS (ESI): m/z = 339.2 $[M+H]^+$; analysis (calcd., found for $C_{20}H_{18}O_5$): C (71.00, 70.83), H (5.36, 5.56).

Supplementary Note 26

Synthesis of 1 and 2: Preparation of Glyceollin II (2)

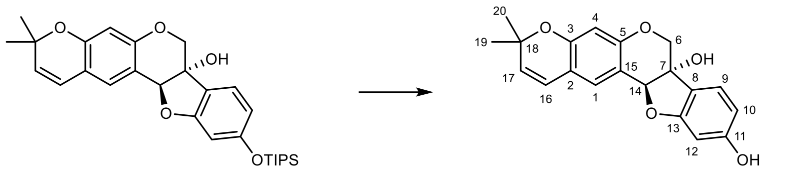

9-*O*-TIPS-Glyceollin II (**27**, 46.0 mg, 93.0 µmol, >99% *ee*) was dissolved in THF (1.9 mL) in a 10 mL round-bottomed flask and cooled to 0 °C. A solution of tetrabutylammonium fluoride (1 M in THF containing 5 % water, 0.14 mL, 0.14 mmol, 1.5 equiv.) was added dropwise, and the resulting solution was stirred for 11 min at 0 °C. The reaction mixture was quenched with water at 0 °C, diluted with saturated aqueous NH_4Cl and extracted three times with Et_2O. The combined organic layers were dried over $MgSO_4$, and the solvents were removed at 25 °C in vacuo. The residue was purified by flash chromatography (Et_2O:pentane 3:1 *v/v*), the solvents were removed in vacuo, and the product was dissolved in methanol (1 mL). Water (2 mL) was added, the solvents were removed in vacuo, and the resulting white solid was dried over P_2O_5 to yield glyceollin II (**2**, 30.8 mg, 98%, >99% *ee*) as a white solid.

mp: 101–102 °C (lit.[15] 89–93 °C); TLC (Et_2O:pentane 3:1 *v/v*): R_f = 0.33; $[\alpha]_D^{20}$ = –307.0 (*c* 0.13 EtOAc); ^1H NMR (600 MHz, acetone-d_6): δ 8.46 (s, 1H, OH-11), 7.21 (d, 1H, $^3J_{9/10}$ = 7.9 Hz, H-9), 7.14 (s, H, H-1), 6.43 (dd, 1H, $^3J_{10/9}$ = 8.1 Hz, $^4J_{10/12}$ = 2.1 Hz, H-10), 6.41 (d, 1H, $^3J_{16/17}$ = 9.8 Hz, H-16), 6.25 (d, 1H, $^4J_{12/10}$ = 2.3 Hz, H-12), 6.21 (s, 1H, H-4), 5.65 (d, 1H,

$^{3}J_{17/16}$ = 9.8 Hz, H-17), 5.25 (s, 1H, H-14), 4.95 (s, 1H, OH-7), 4.13 (d, 1H, $^{2}J_{6a/6b}$ = 11.3 Hz, H-6a), 4.04 (d, 1H, $^{2}J_{6b/6a}$ = 11.3 Hz, H-6b), 1.39 (s, 3H, H-19), 1.37 (s, 3H, H-20) ppm; ^{13}C (151 MHz, acetone-d_6): δ 162.04 (C, C-13), 160.84 (C, C-11), 156.91 (C, C-5), 155.33 (C, C-3), 130.02 (CH, C-17), 129.87 (CH, C-1), 125.27 (CH, C-9), 122.38 (CH, C-16), 121.45 (C, C-8), 117.25 (C, C-2), 114.56 (C, C-15), 109.07 (CH, C-10), 104.89 (CH, C-4), 98.79 (CH, C-12), 85.84 (CH, C-14), 77.29 (C, C-18), 76.76 (C, C-7), 70.67 (CH_2, C-6), 28.41 (CH_3, C-19/C-20), 28.36 (CH_3, C-20/C-19) ppm; IR (ATR): 3367, 1622, 1492, 1122, 962 cm^{-1}; MS (ESI): m/z = 339.3 $[M+H]^+$; analysis (calcd., found for $C_{20}H_{18}O_5$): C (71.00, 70.64), H (5.36, 5.67).

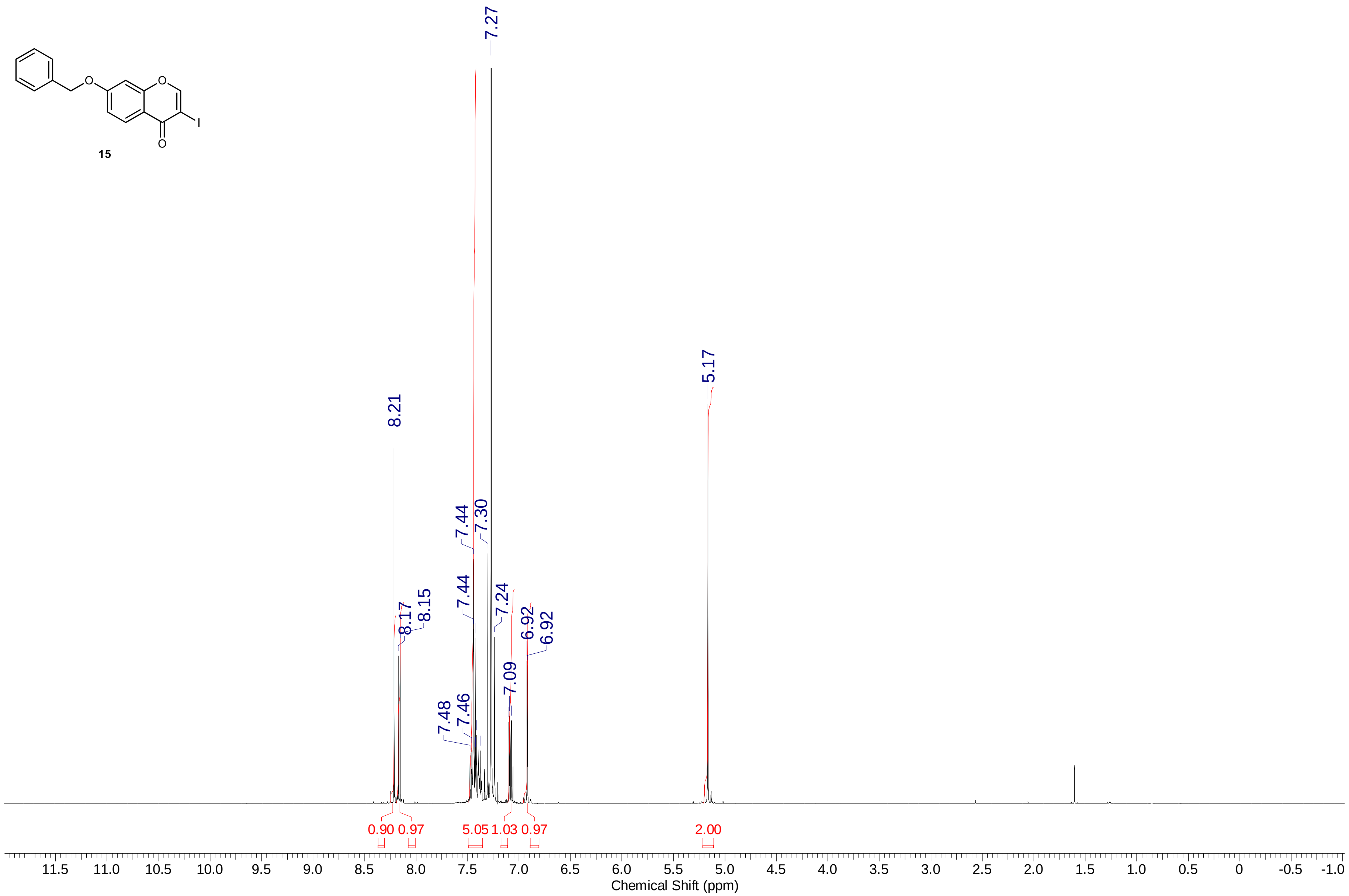

Supplementary Figure 1. ^{1}H NMR of **15**

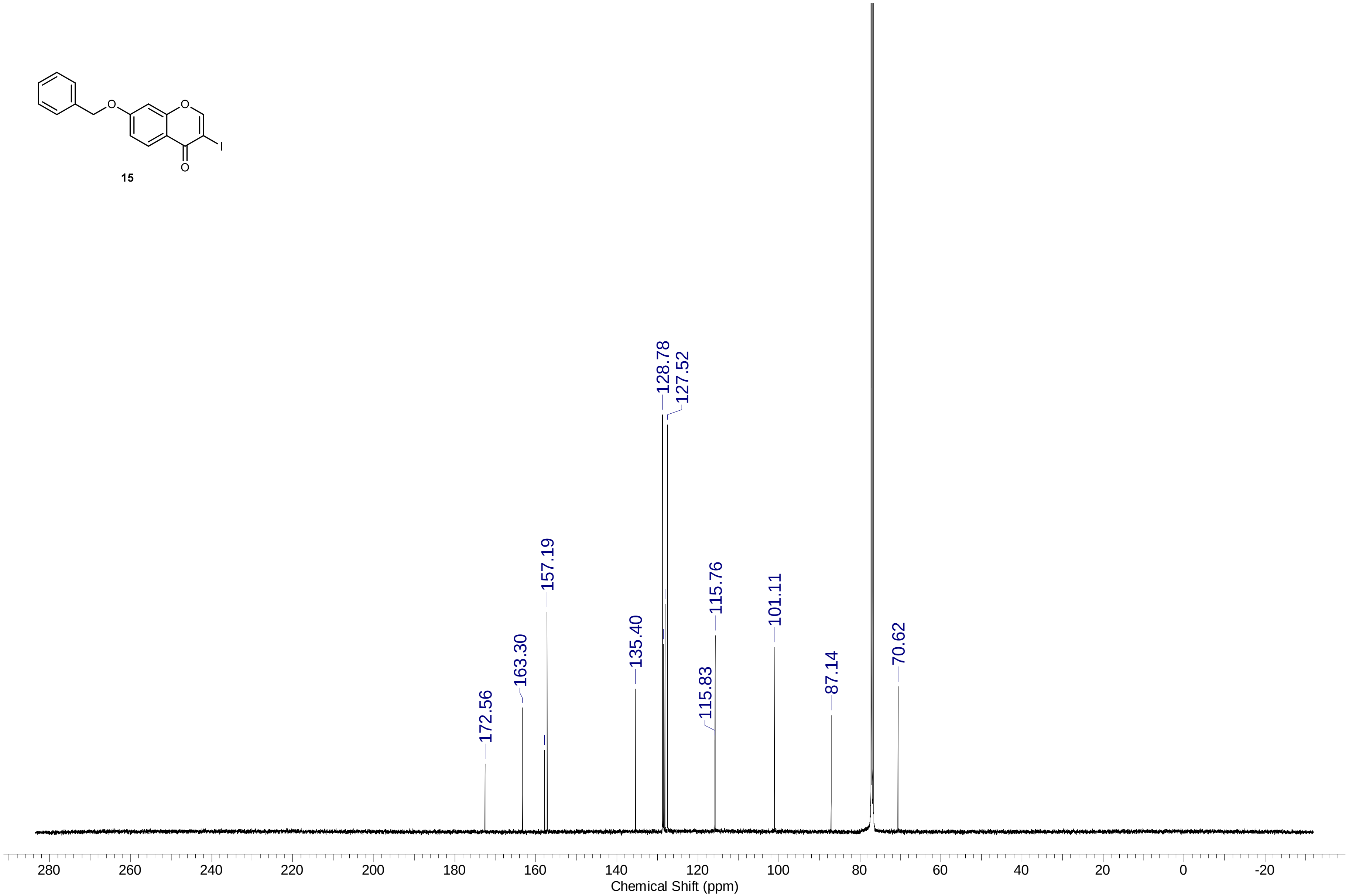

Supplementary Figure 2. ^{13}C NMR of **15**

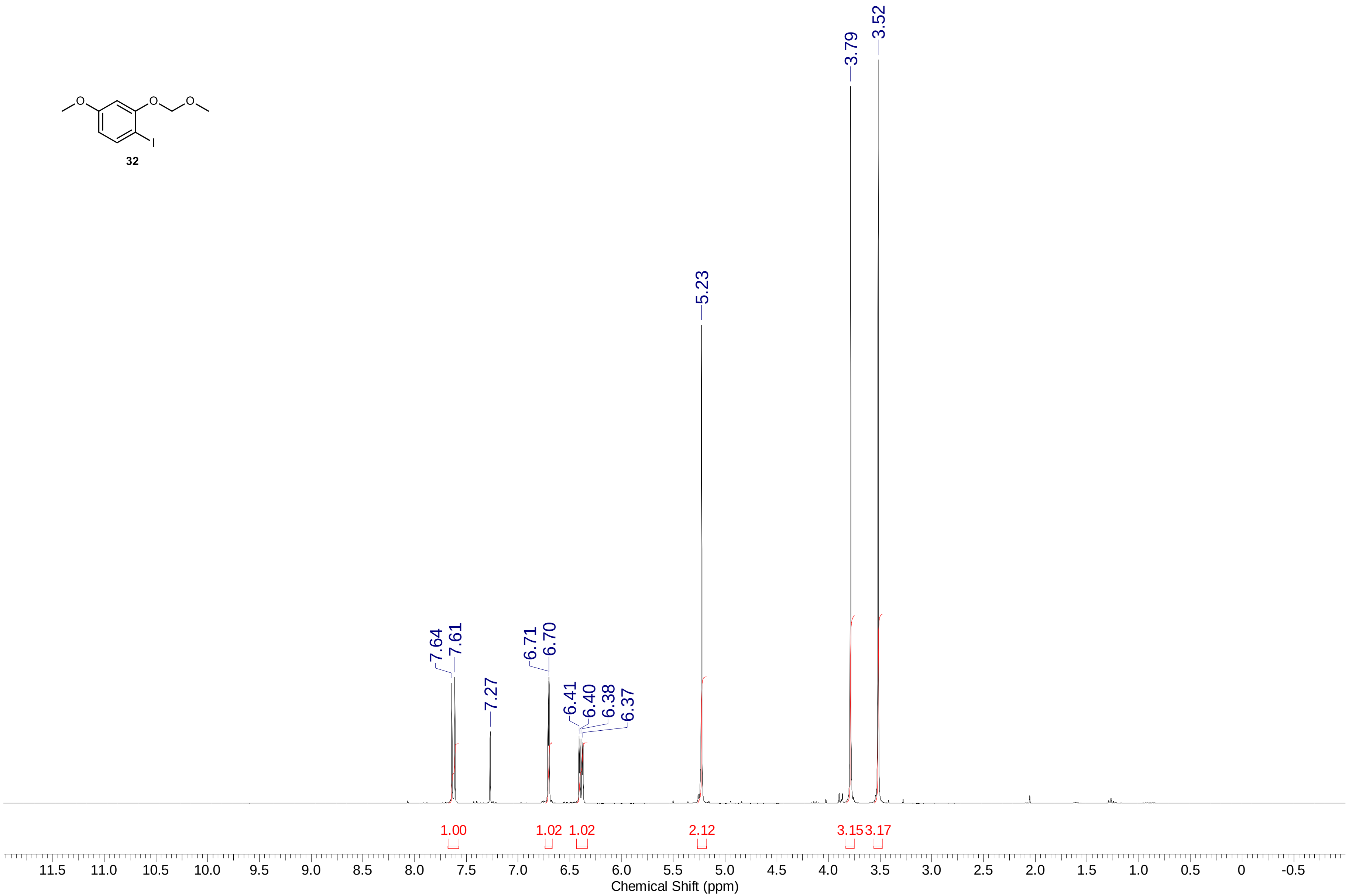

Supplementary Figure 3. ^{1}H NMR of **32**

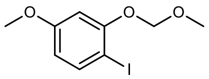

32

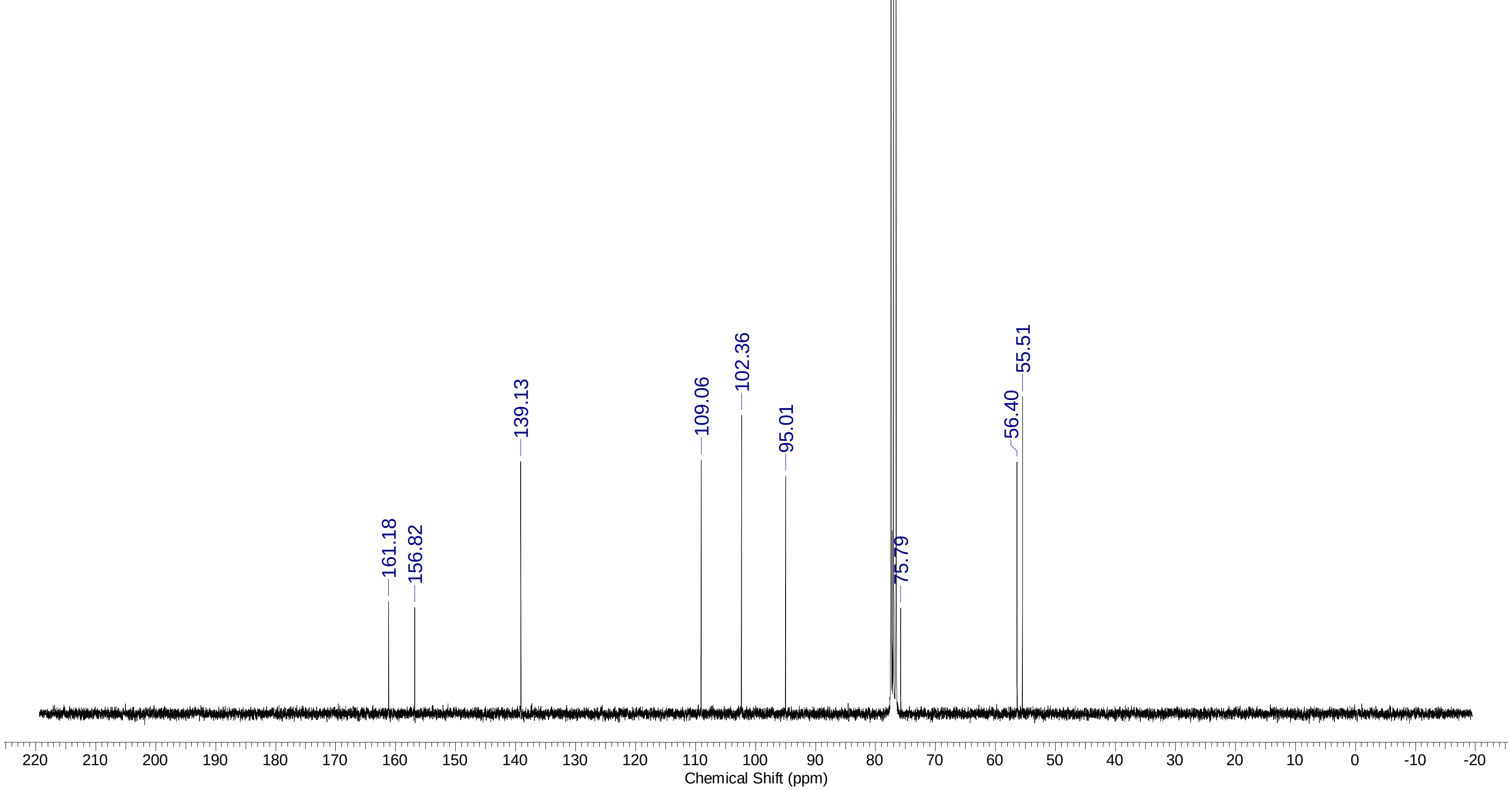

Supplementary Figure 4. ^{13}C NMR of **32**

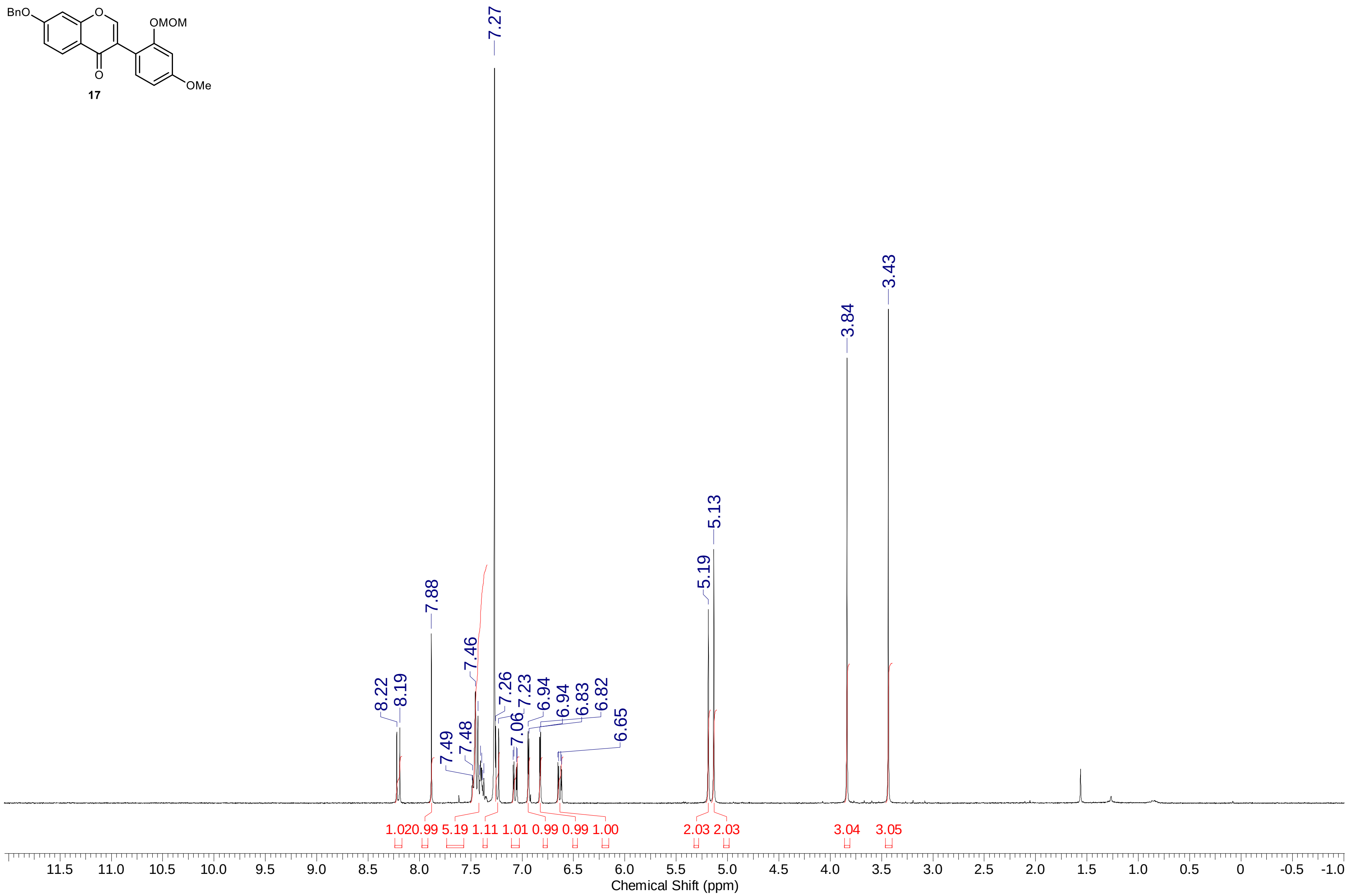

Supplementary Figure 5. ^{1}H NMR of **17**

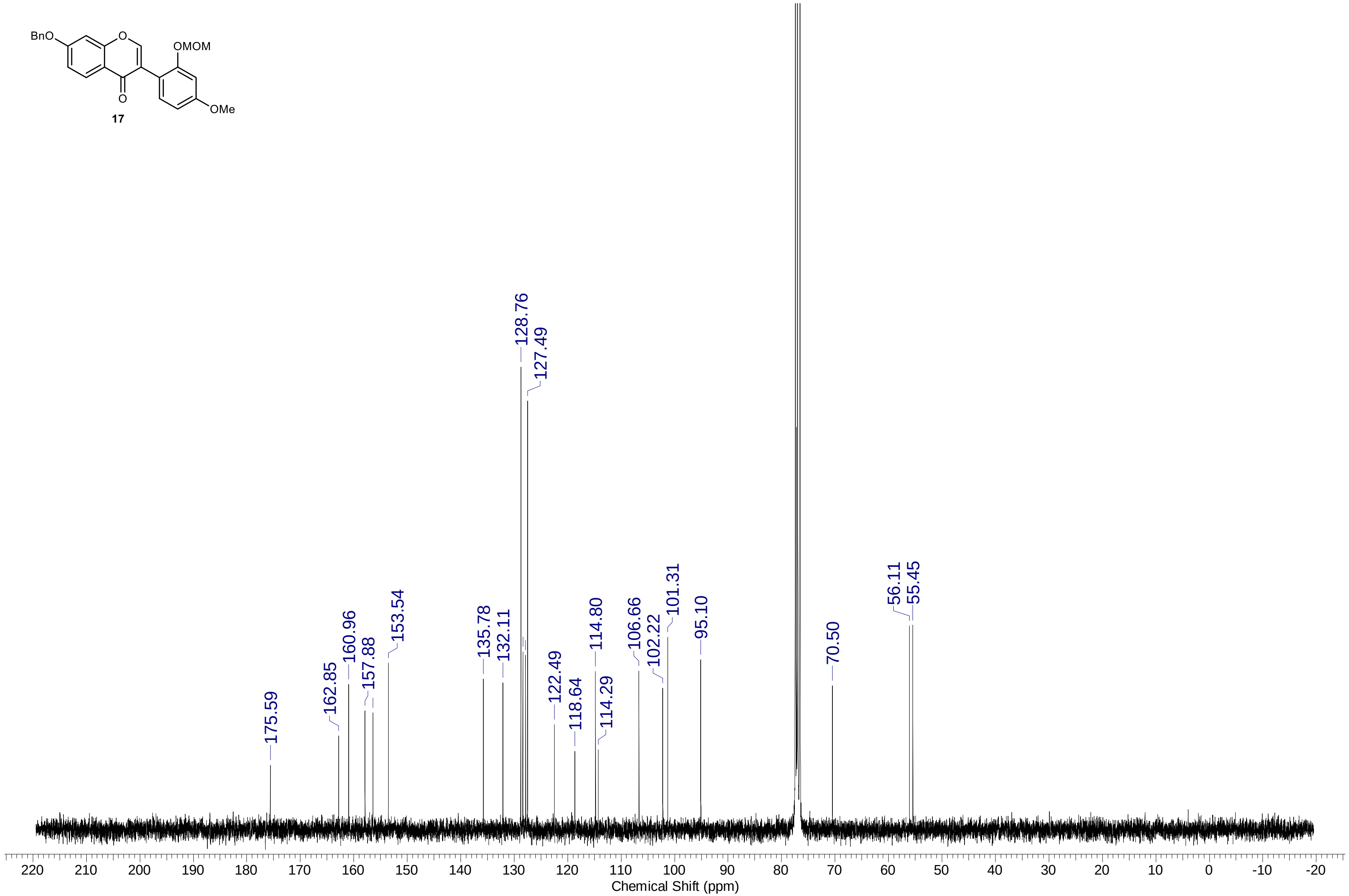

Supplementary Figure 6. ^{13}C NMR of **17**

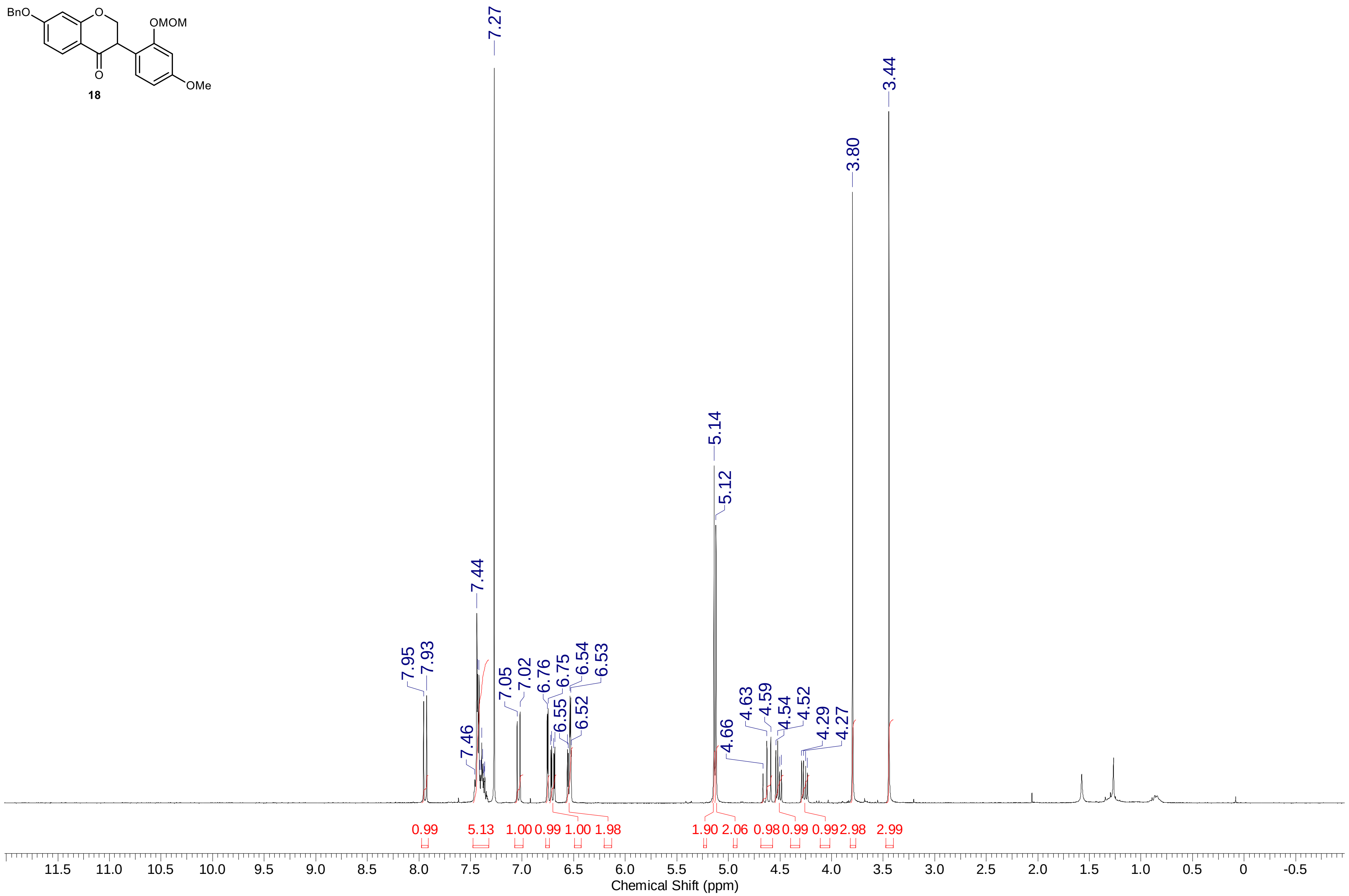

Supplementary Figure 7. ^{1}H NMR of **18**

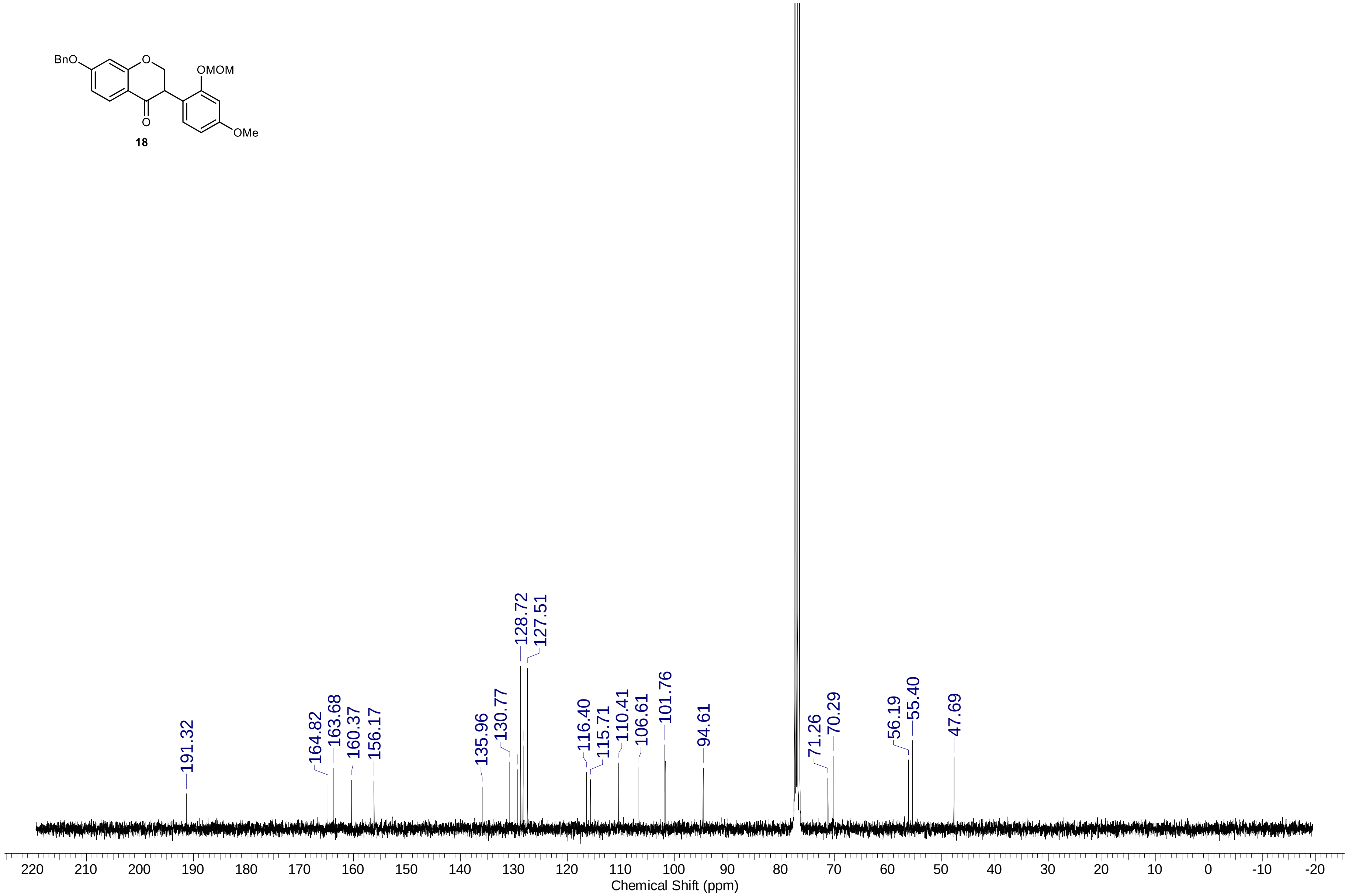

Supplementary Figure 8. ^{13}C NMR of **18**

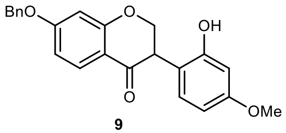

9

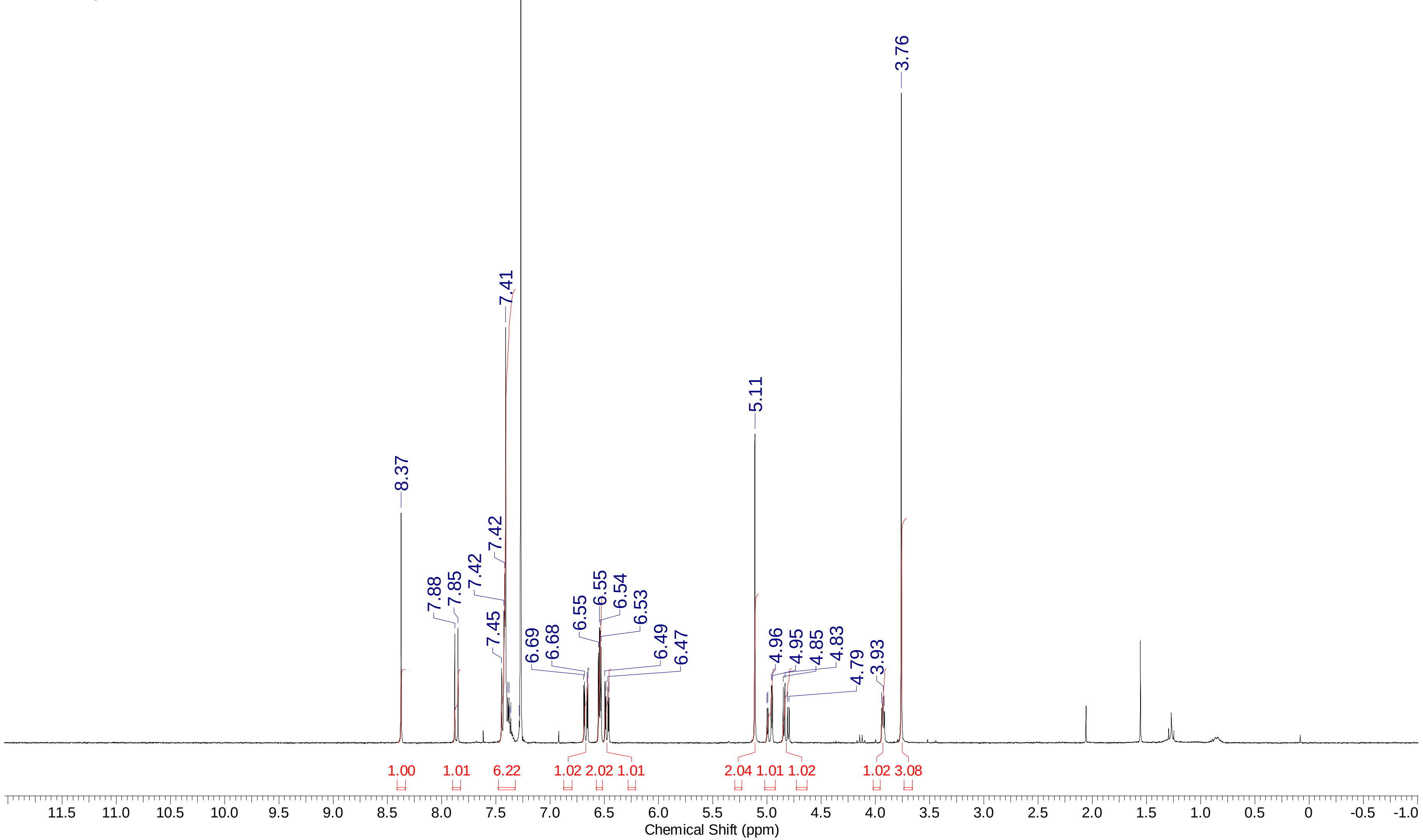

Supplementary Figure 9. ^{1}H NMR of **9**

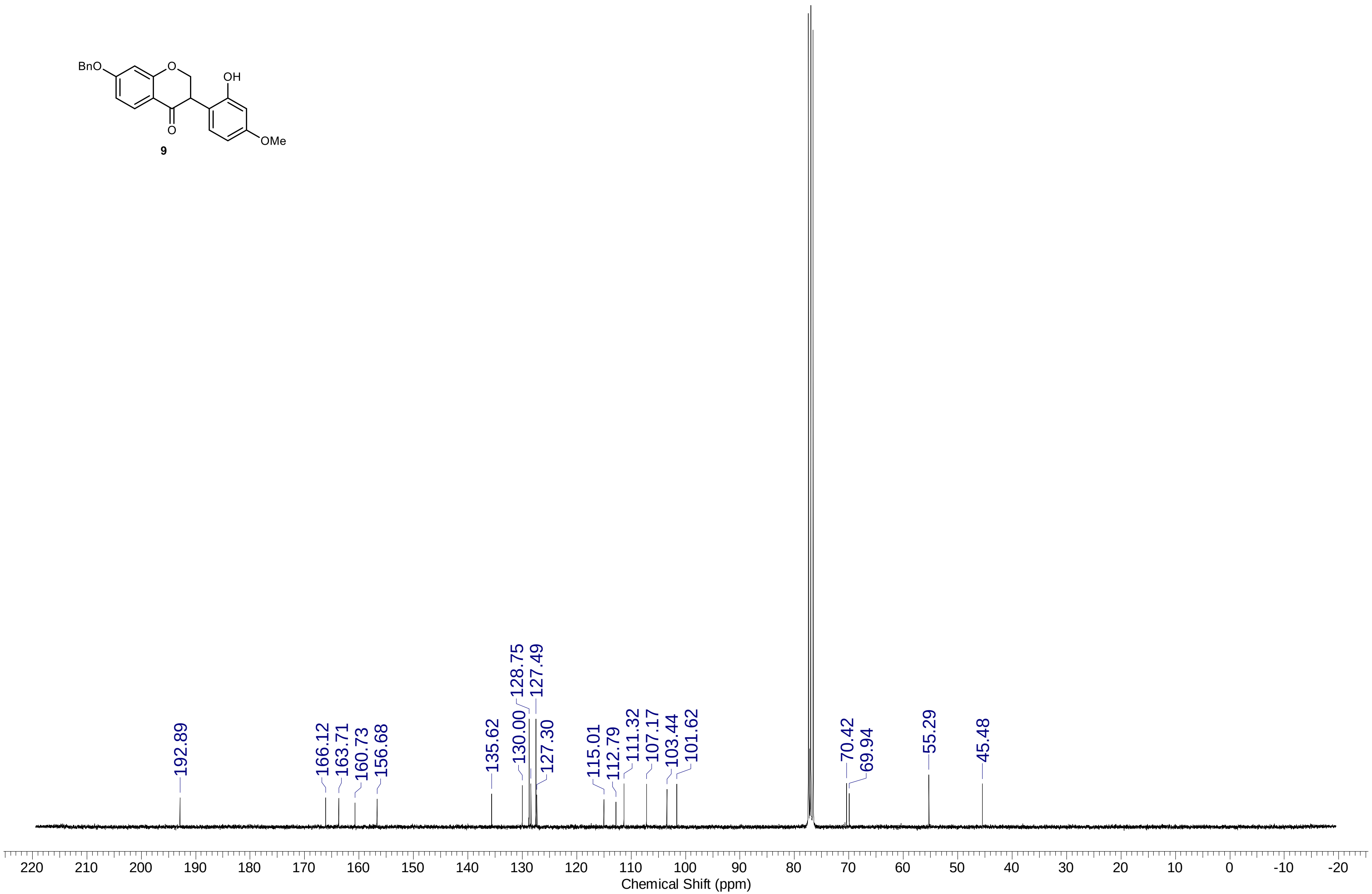

Supplementary Figure 10. ^{13}C NMR of **9**

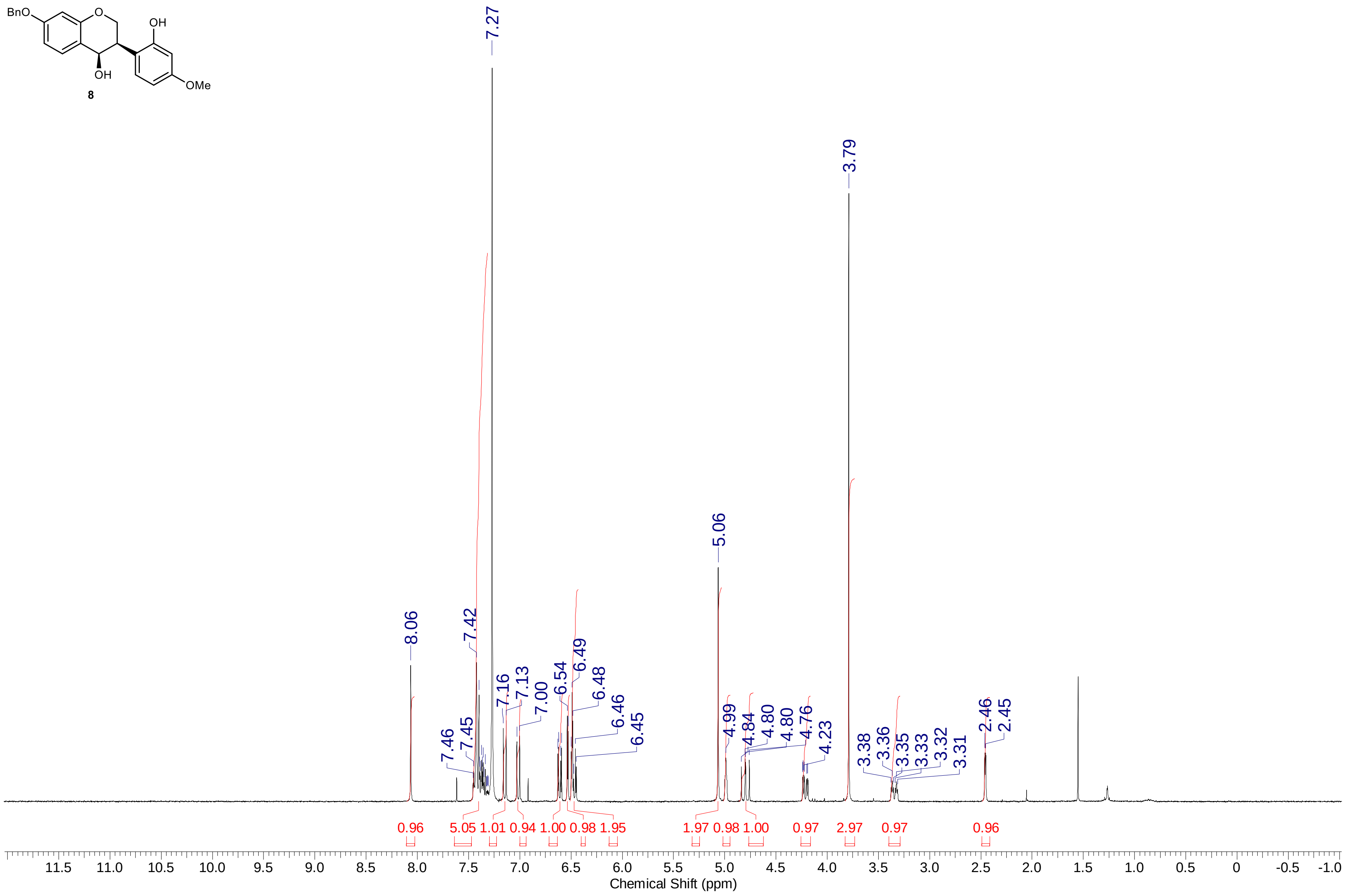

Supplementary Figure 11. ^{1}H NMR of **8**

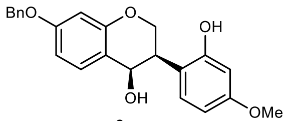

8

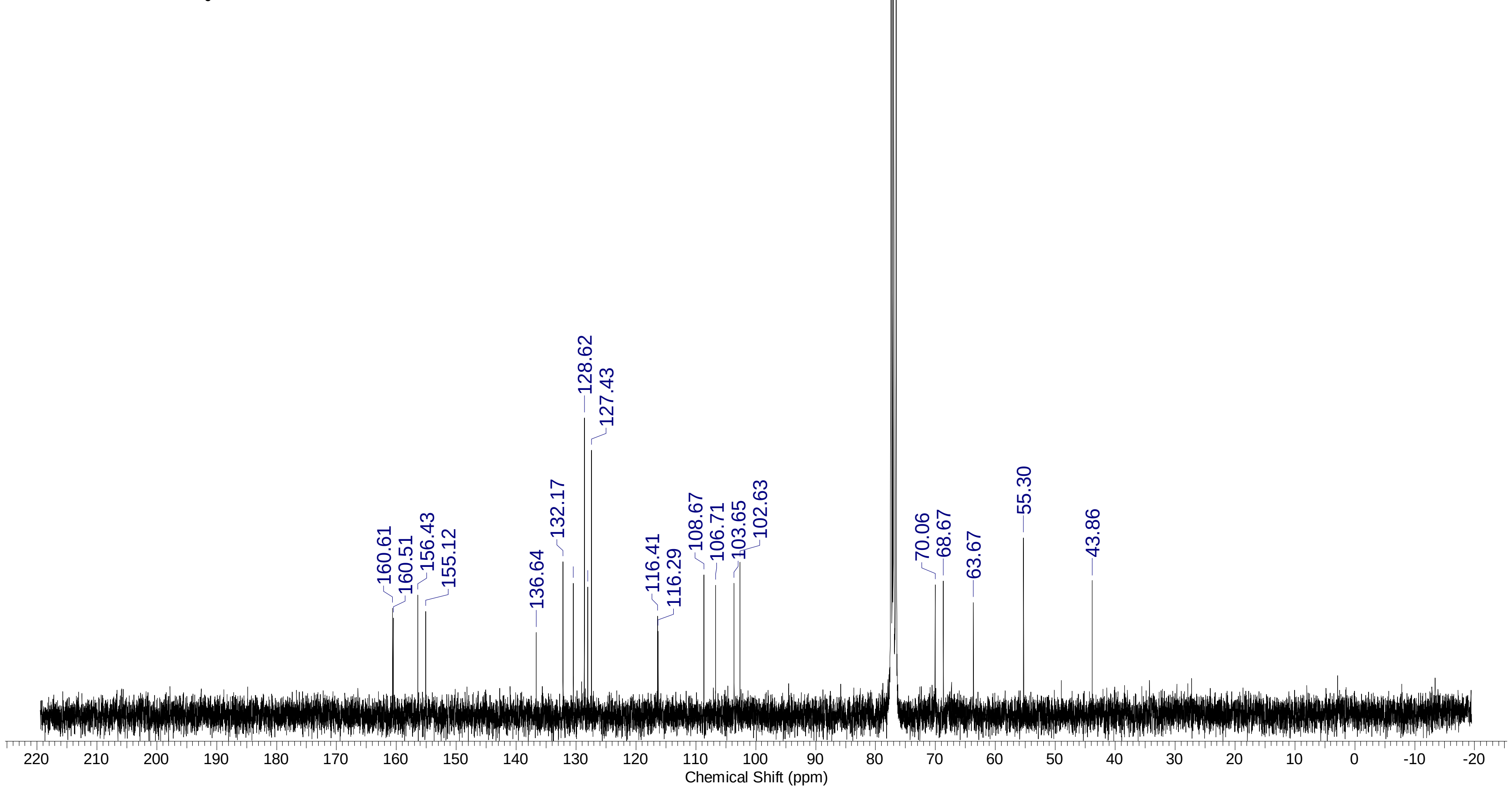

Supplementary Figure 12. ^{13}C NMR of **8**

Chiral HPLC trace of *rac*-**8** (bottom) and (*R*,*R*)-**8** (top, >99% *ee*); Method: Lux Amylose-1, hexane:isopropanol 70:30 *v/v*, 0.8 mL/min

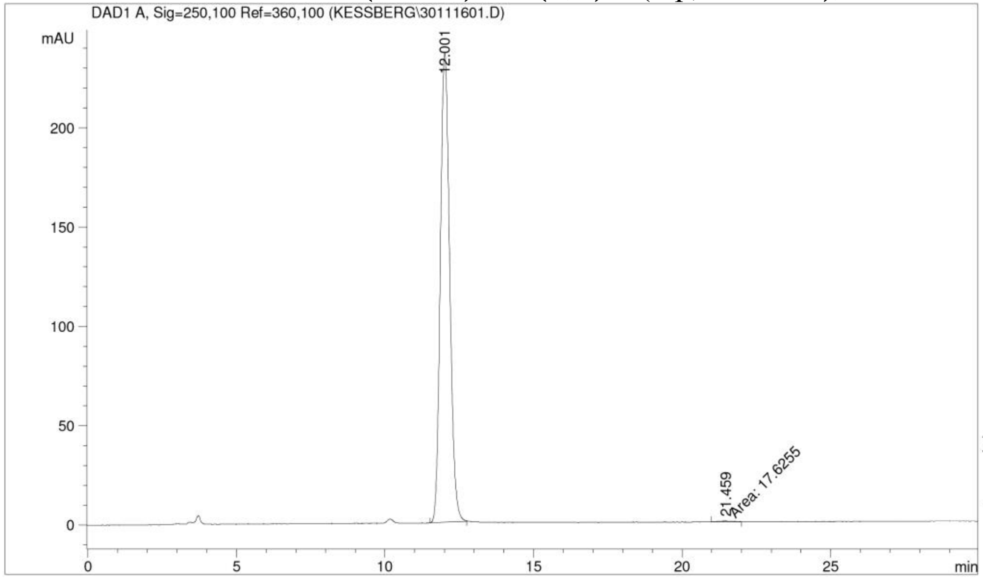

Peak #	RetTime [min]	Type	Width [min]	Area [mAU*s]	Height [mAU]	Area %
1	12.001	BB	0.3396	5171.82568	236.10640	99.6604
2	21.459	MM T	0.6350	17.62548	4.62577e-1	0.3396

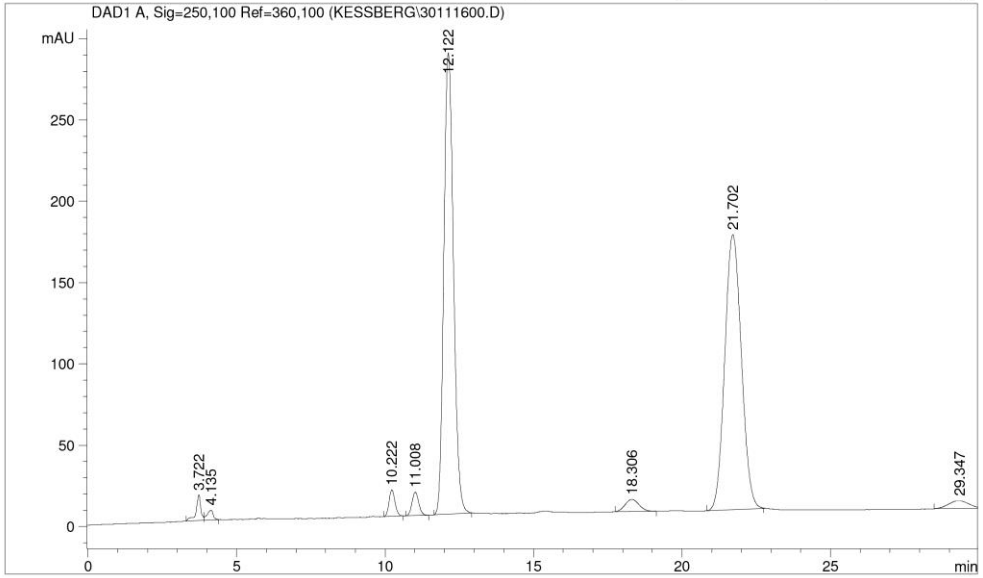

Supplementary Figure 13. Chiral HPLC trace of **8**

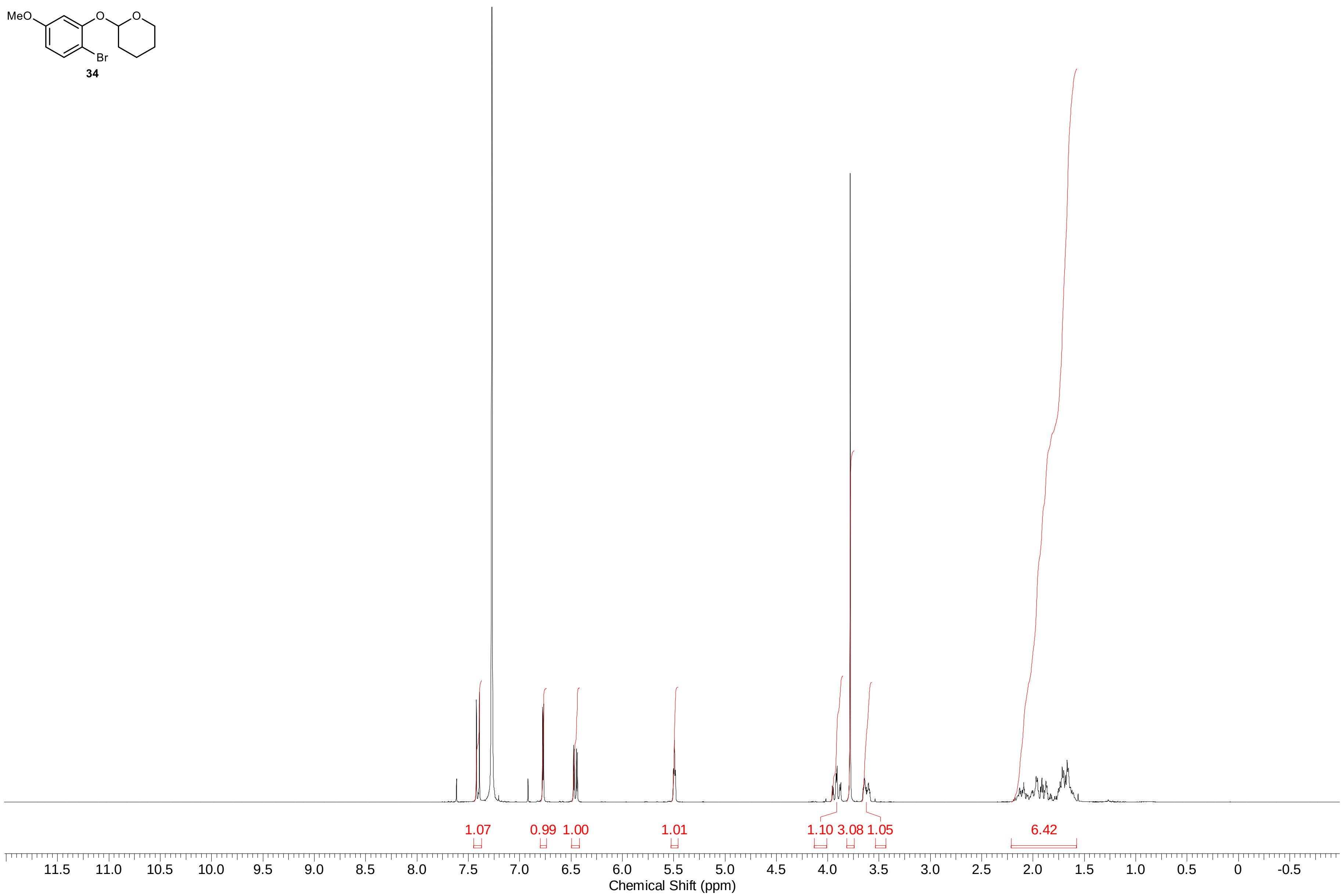

Supplementary Figure 14. ^{1}H NMR of **34**

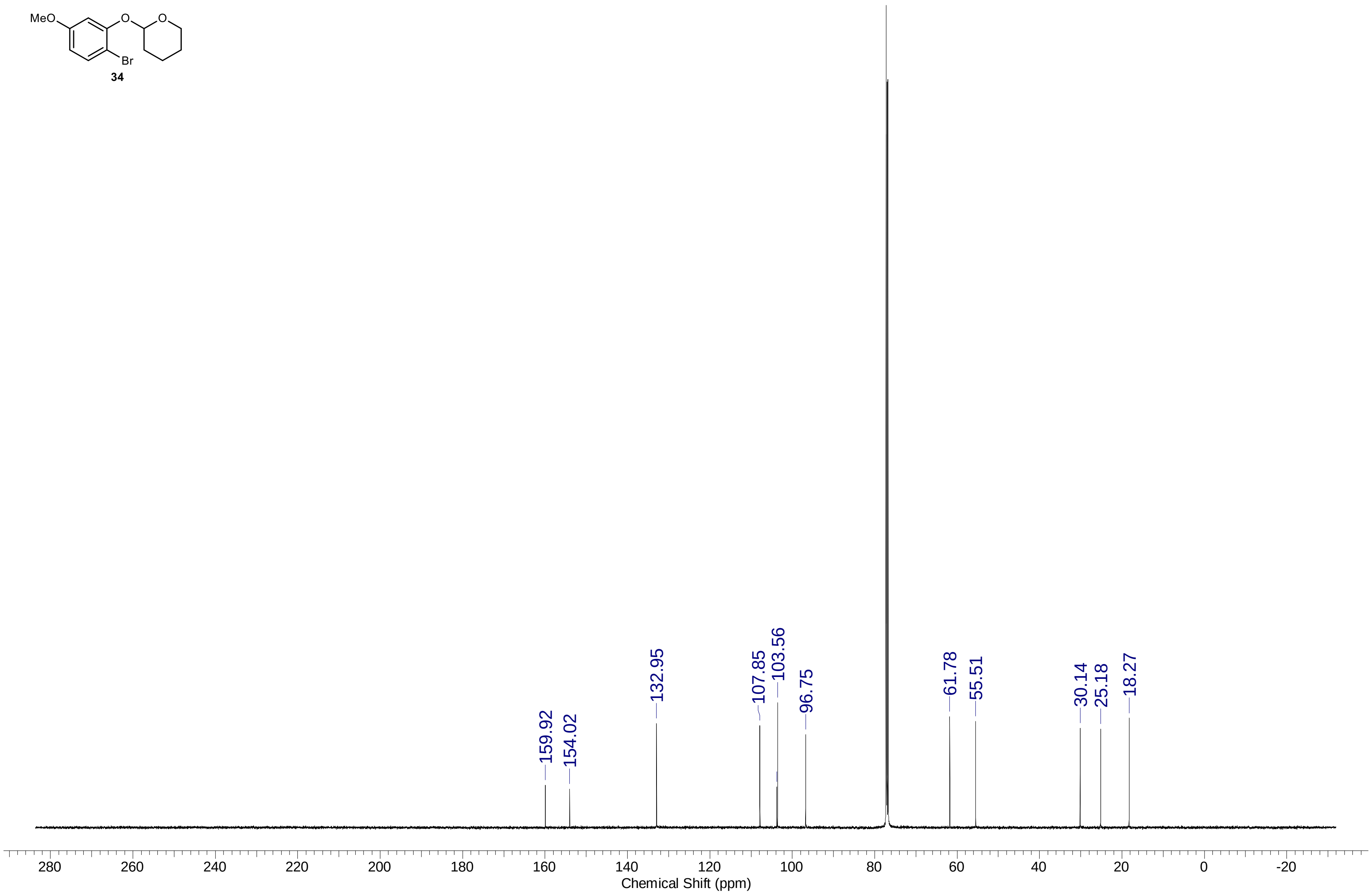

Supplementary Figure 15. ^{13}C NMR of **34**

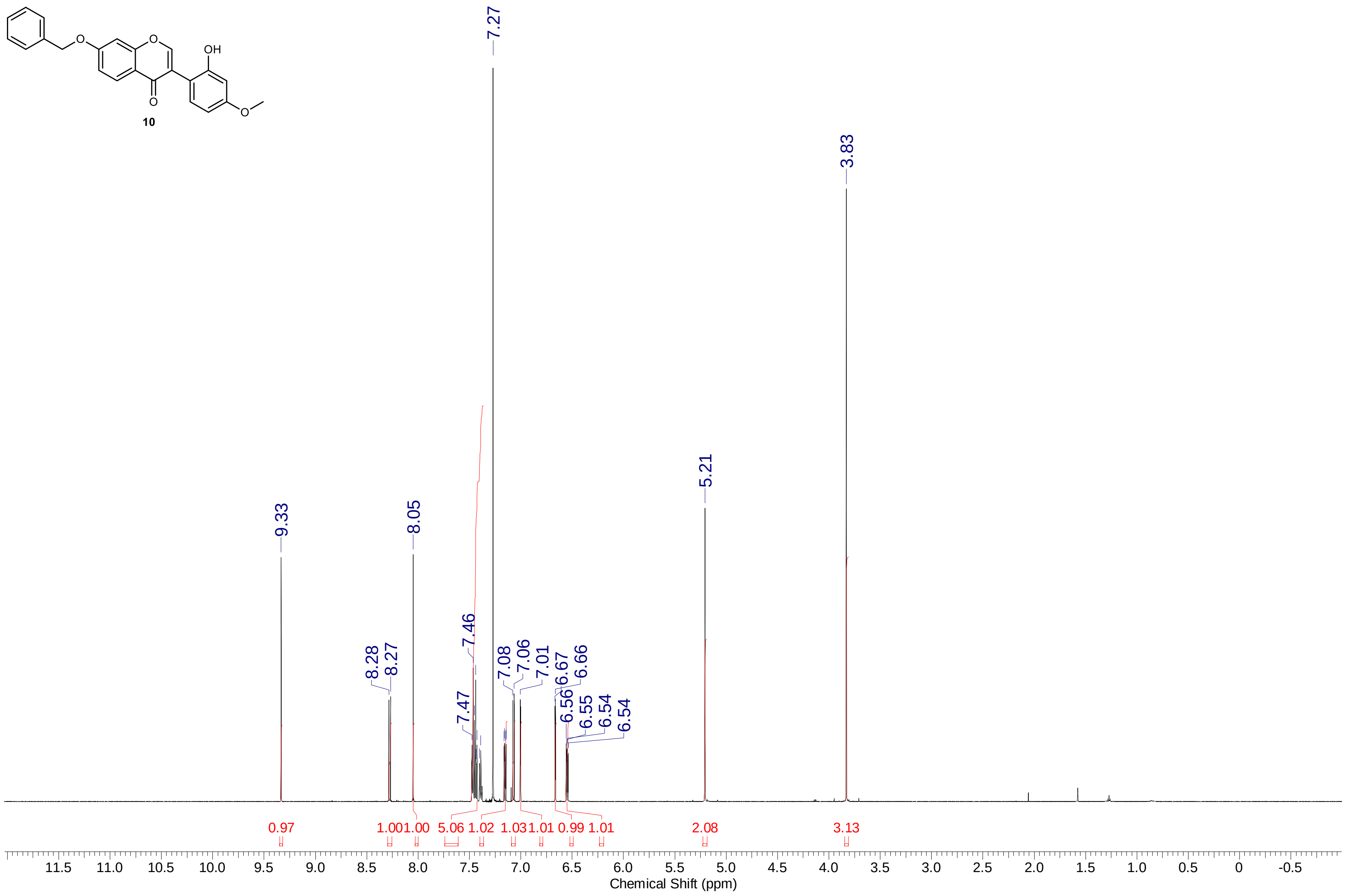

Supplementary Figure 16. ^{1}H NMR of **10**

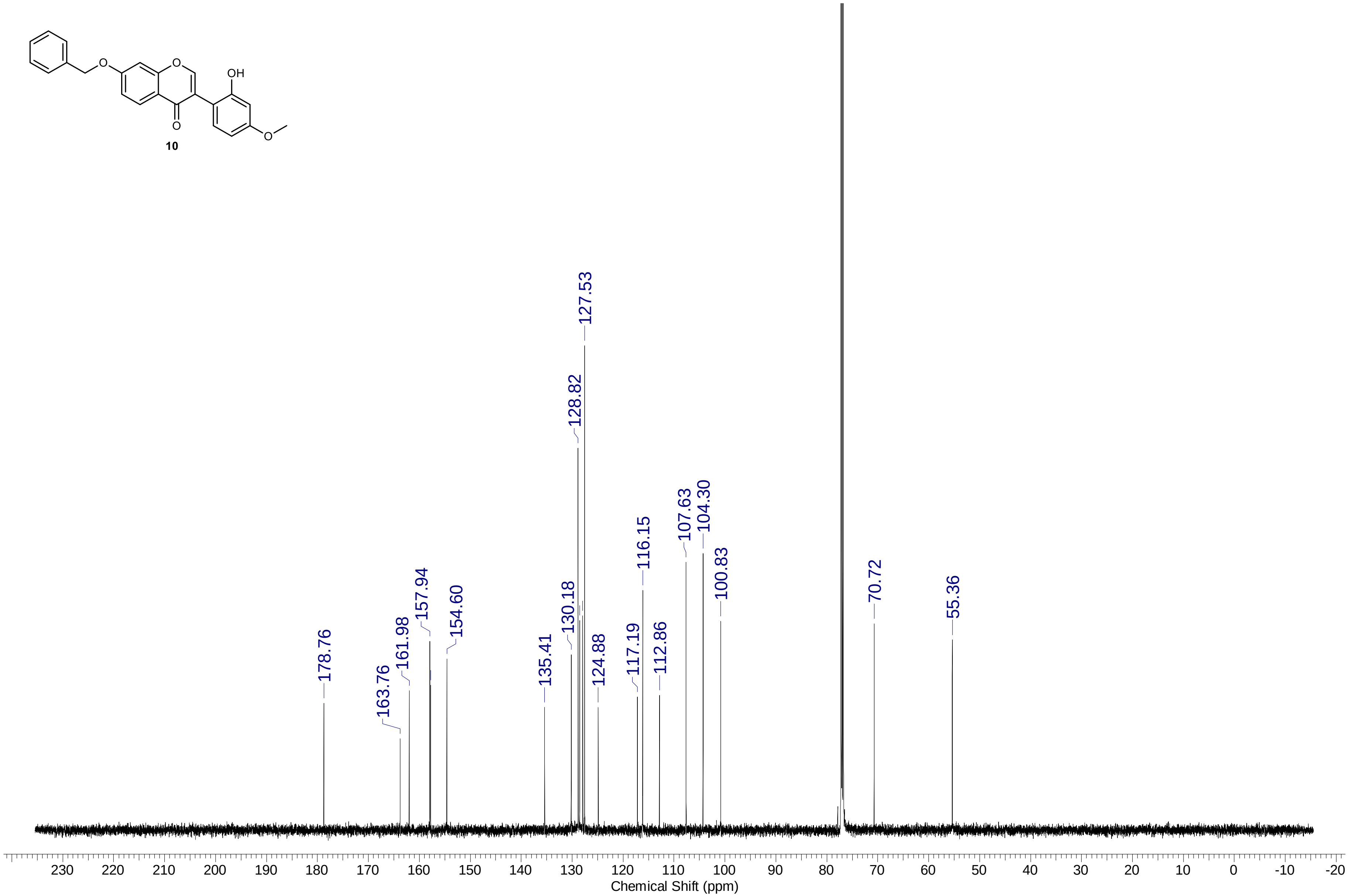

Supplementary Figure 17. ^{13}C NMR of **10**

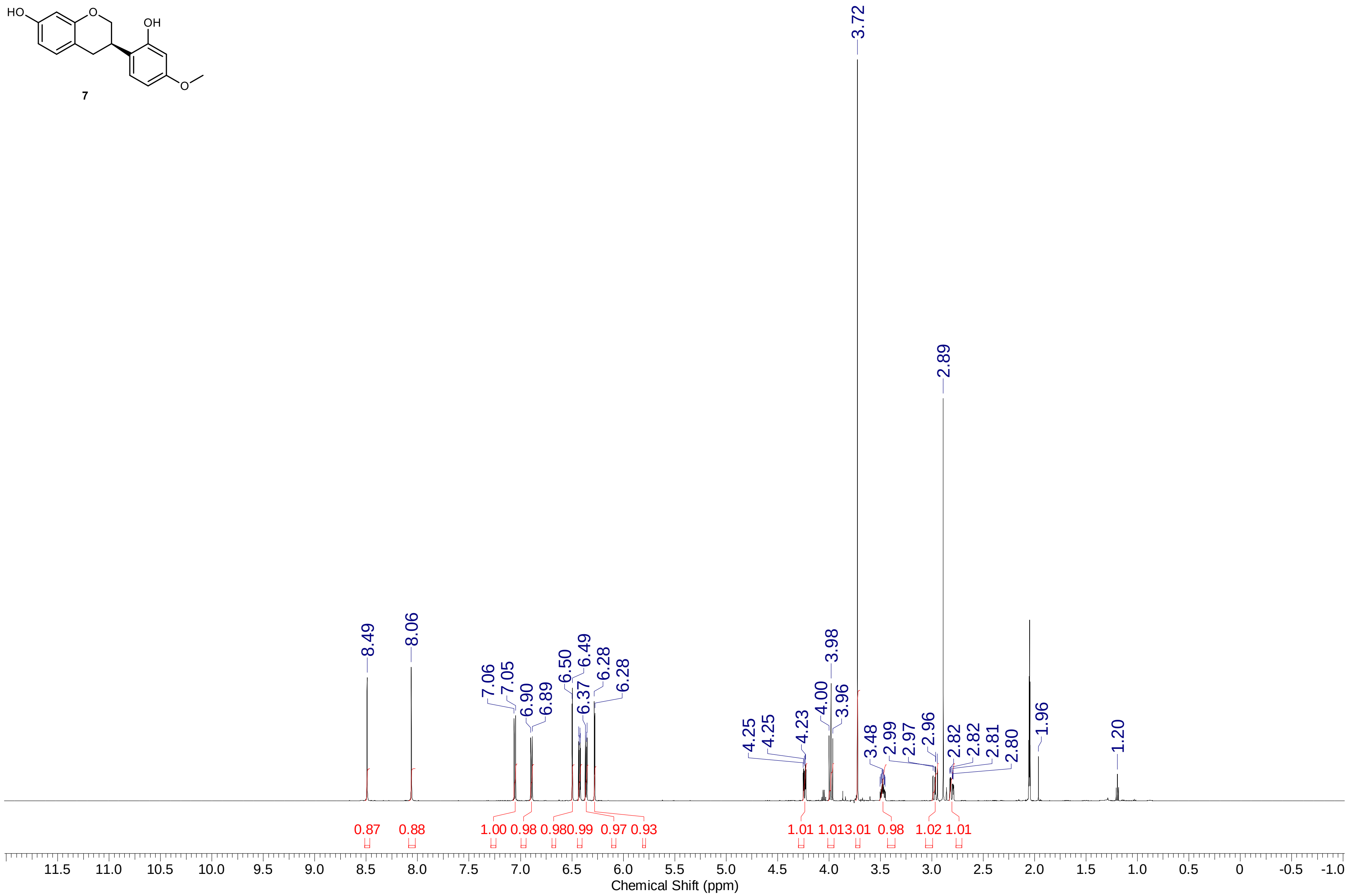

Supplementary Figure 18. ^{1}H NMR of **7**

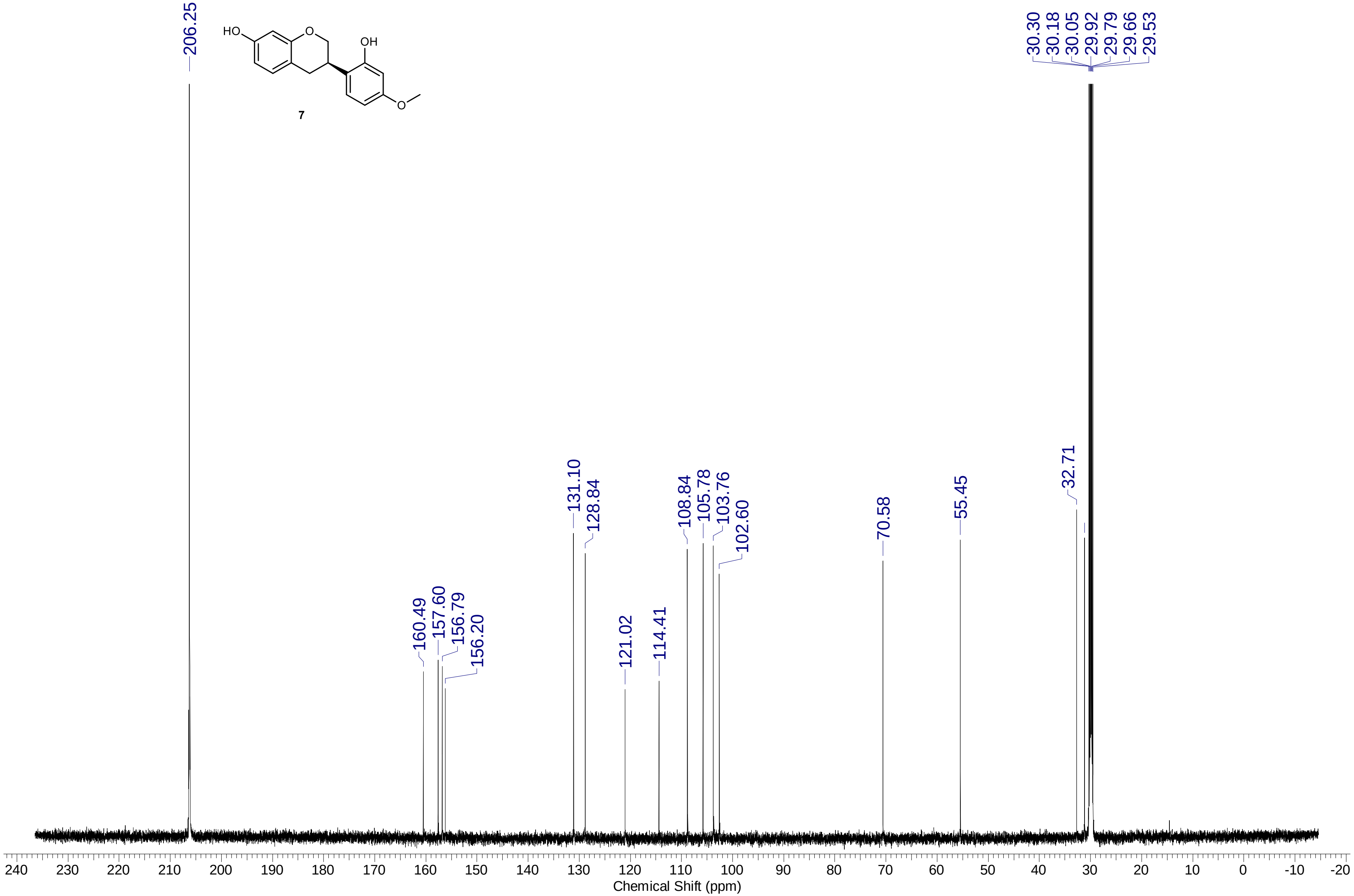

Supplementary Figure 19. ^{13}C NMR of **7**

Chiral HPLC trace of *rac*-**7** (bottom) and (*R*)-**7** (top, 97% *ee*); Method: Lux® Cellulose-1, hexane:isopropanol 80:20 *v/v*, 0.7 mL/min

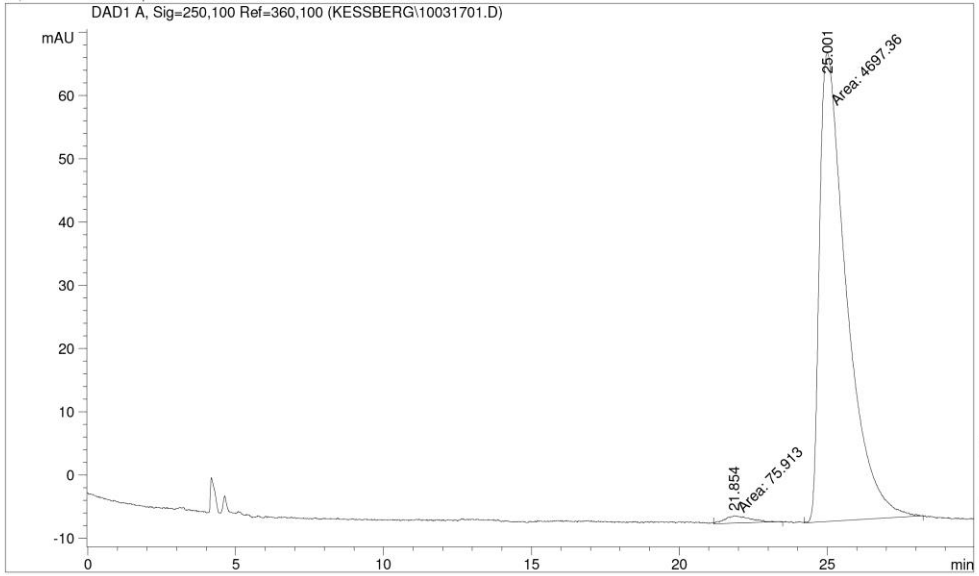

Peak #	RetTime [min]	Type	Width [min]	Area [mAU*s]	Height [mAU]	Area %
1	21.854	MM T	1.0131	75.91296	1.24888	1.5904
2	25.001	MM T	1.0557	4697.35547	74.16153	98.4096

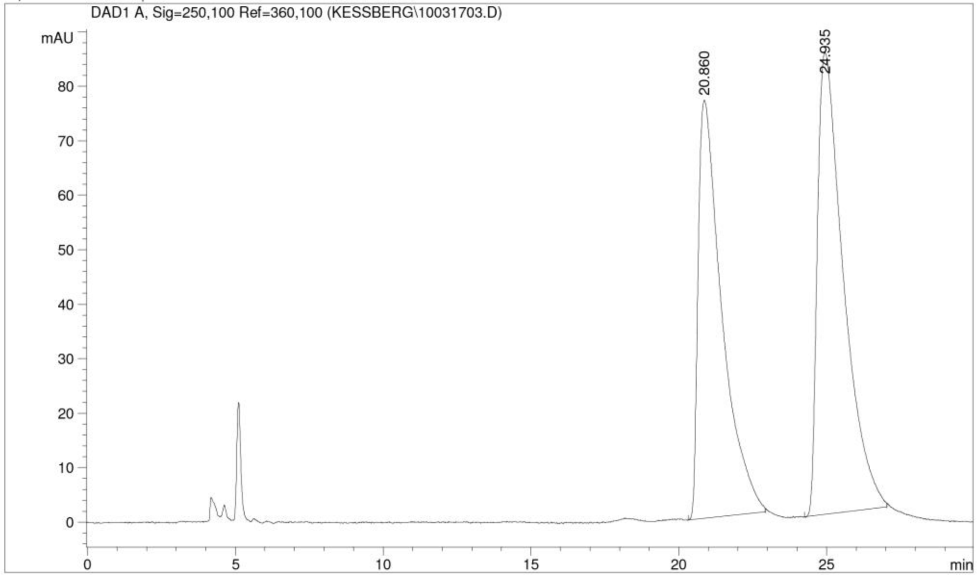

Supplementary Figure 20. Chiral HPLC trace of **7**

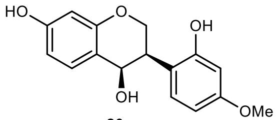

20

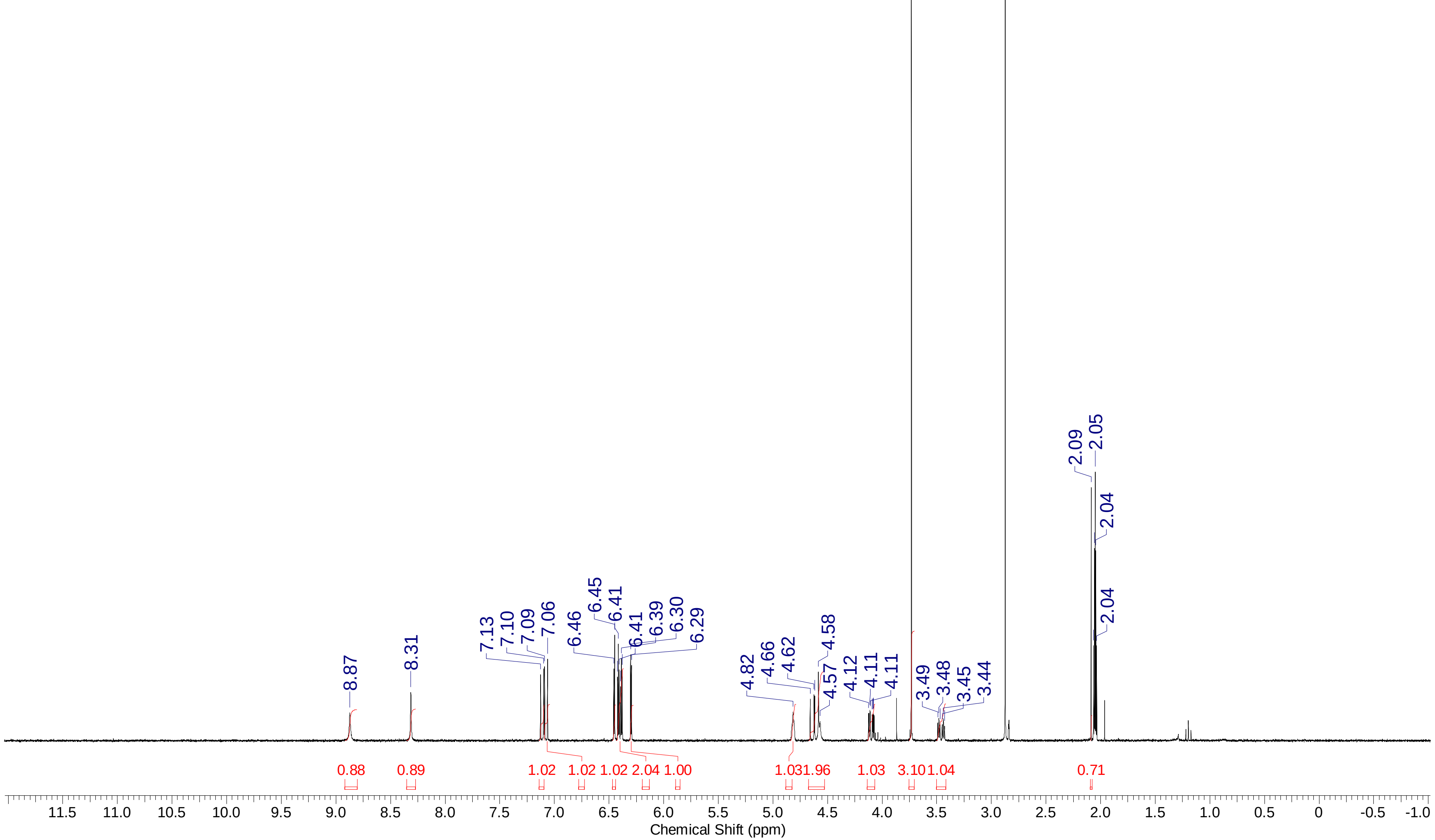

Supplementary Figure 21. ^{1}H NMR of **20**

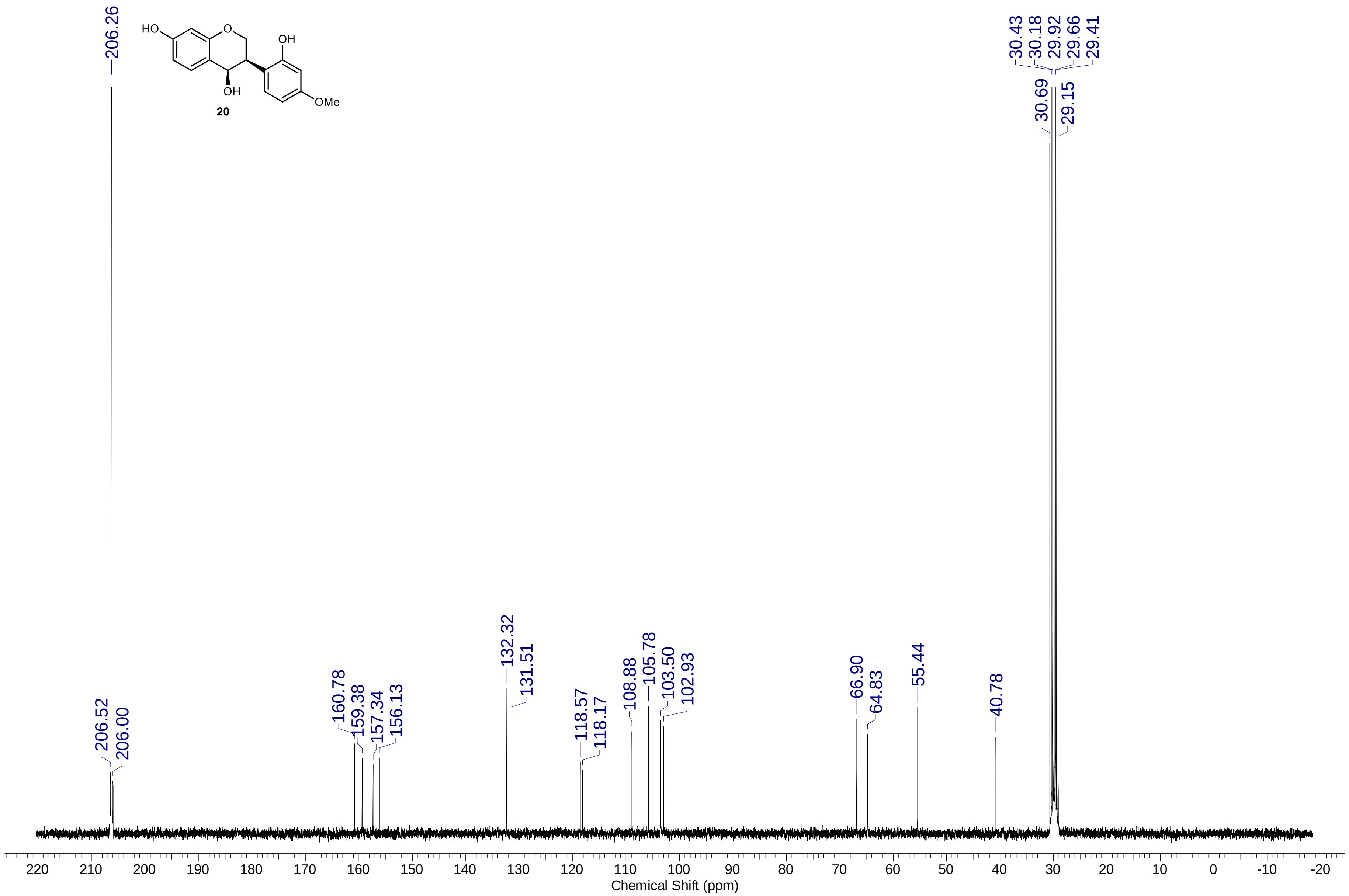

Supplementary Figure 22. ^{13}C NMR of **20**

Chiral HPLC trace of *rac*-**20** (bottom) and (*R*,*R*)-**20** (top, >99% *ee*); Method: Lux Amylose-1, hexane:isopropanol 70:30 *v/v*, 0.8 mL/min

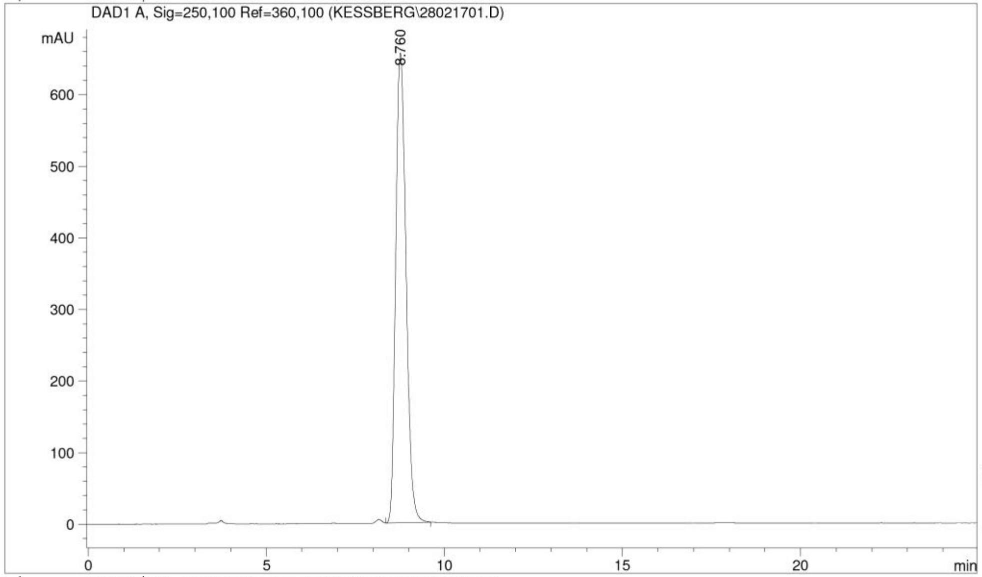

Peak #	RetTime [min]	Type	Width [min]	Area [mAU*s]	Height [mAU]	Area %
1	8.760	VB	0.3184	1.31932e4	656.58435	100.0000

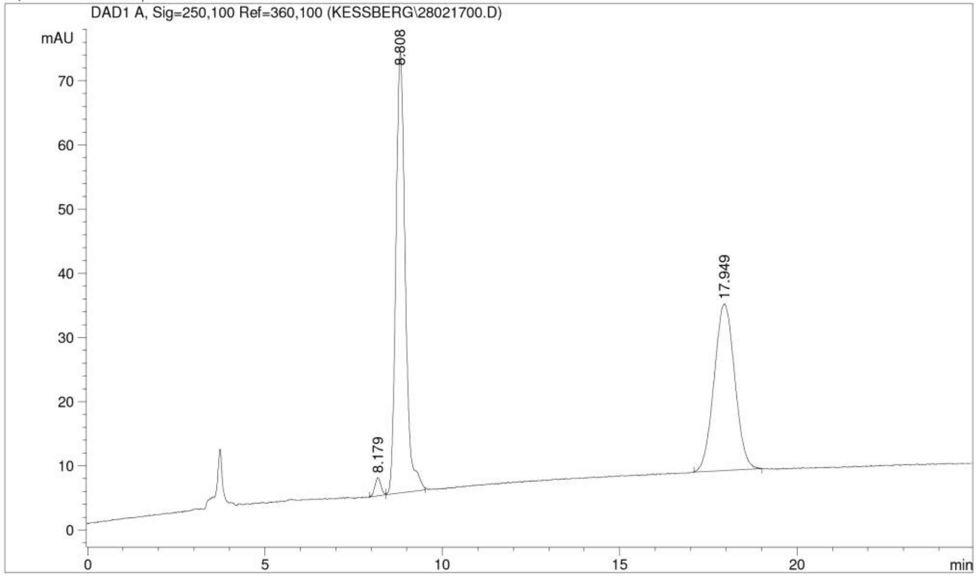

Supplementary Figure 23. Chiral HPLC trace of **20**

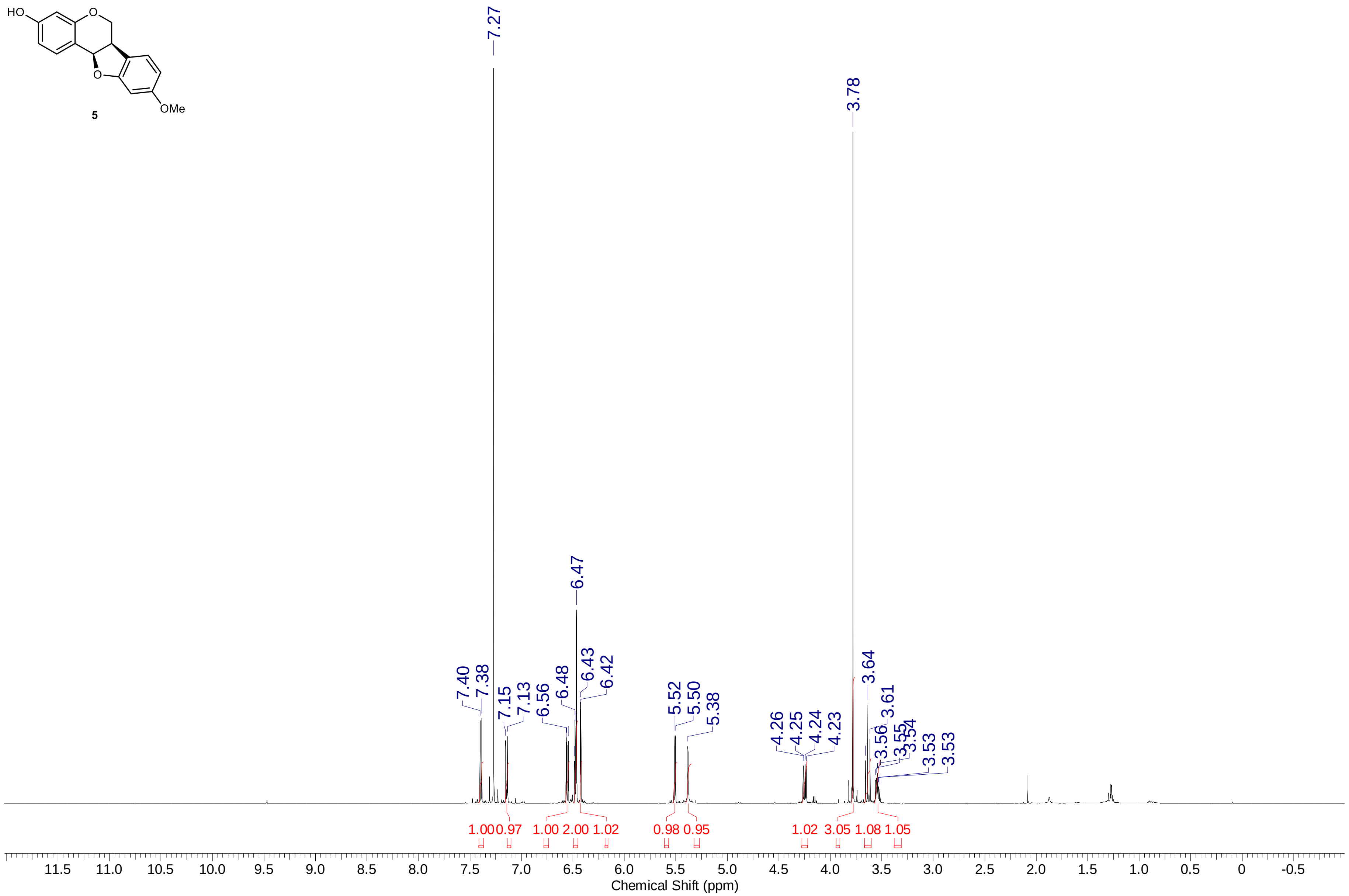

Supplementary Figure 24. ^{1}H NMR of **5**

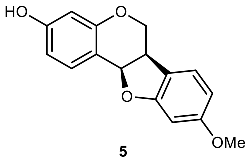

5

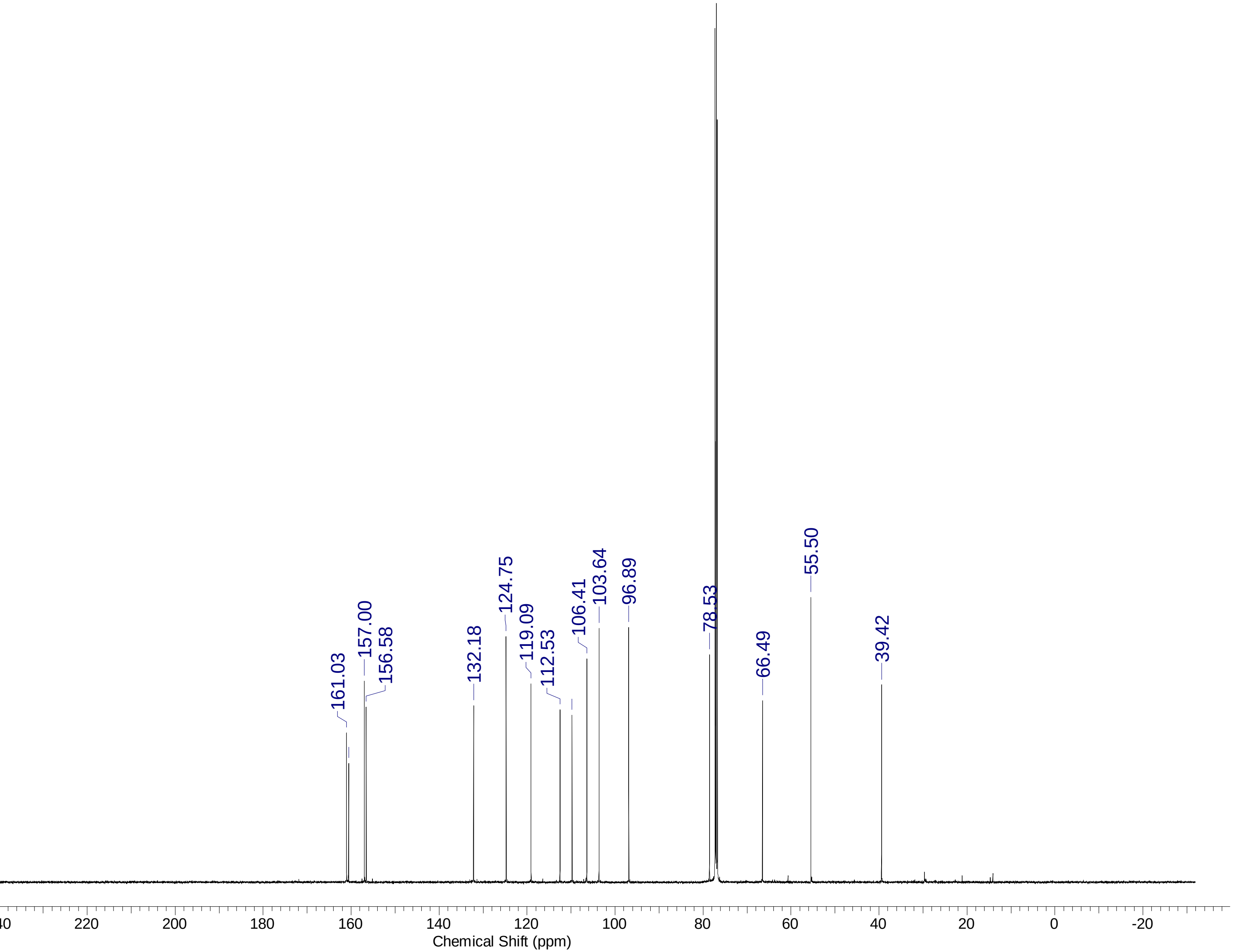

Supplementary Figure 25. ^{13}C NMR of **5**

Chiral HPLC trace of *rac*-**5** (bottom) and (*R*,*R*)-**5** (top, >99% *ee*); Method: Chiralpak® AD, hexane:isopropanol 80:20 *v/v*, 0.8 mL/min

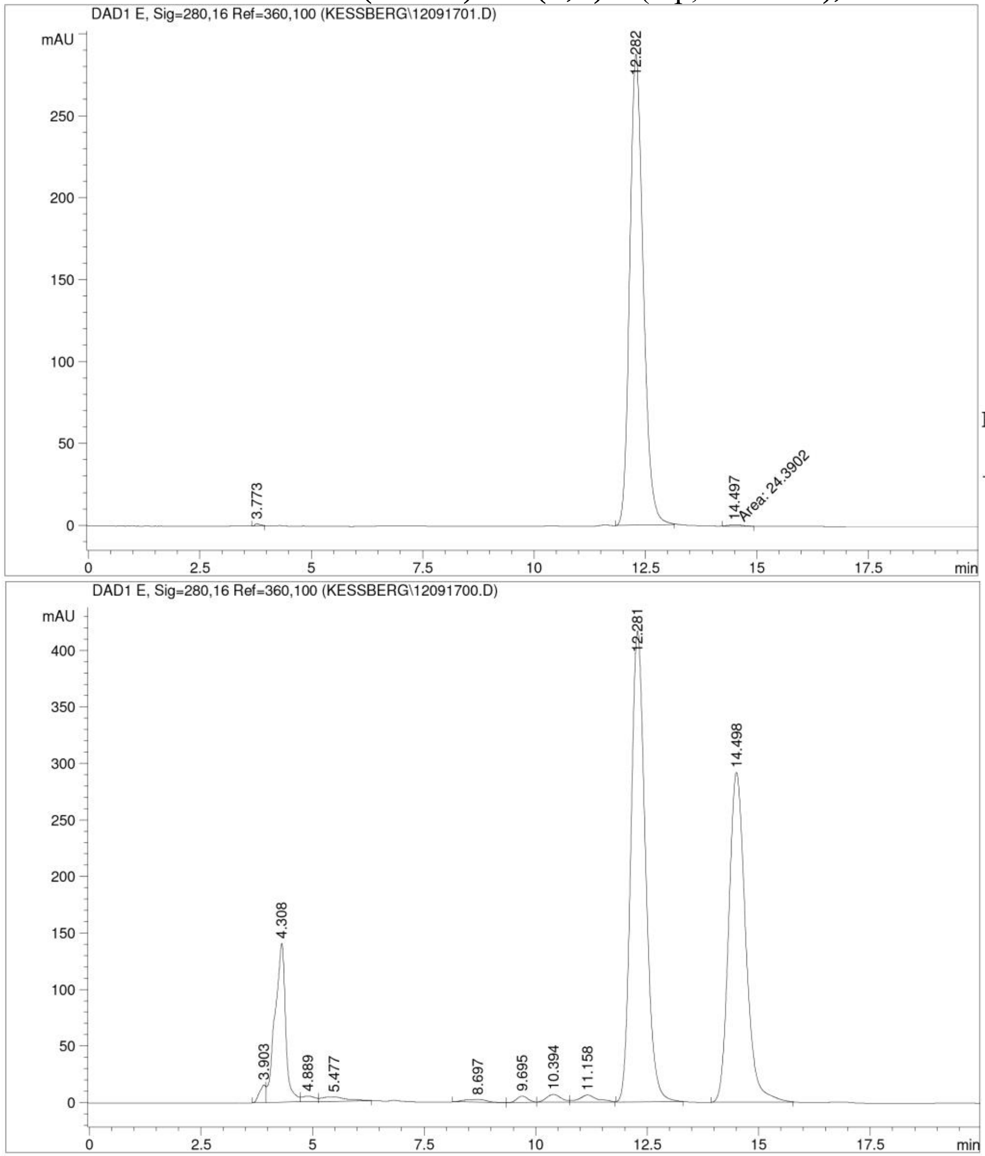

Peak #	RetTime [min]	Type	Width [min]	Area [mAU*s]	Height [mAU]	Area %
1	3.773	PB	0.1216	11.36142	1.38586	0.1868
2	12.282	PB	0.3233	6045.40479	287.47052	99.4121
3	14.497	MM T	0.4044	24.39021	1.00528	0.4011

Supplementary Figure 26. Chiral HPLC trace of **5**

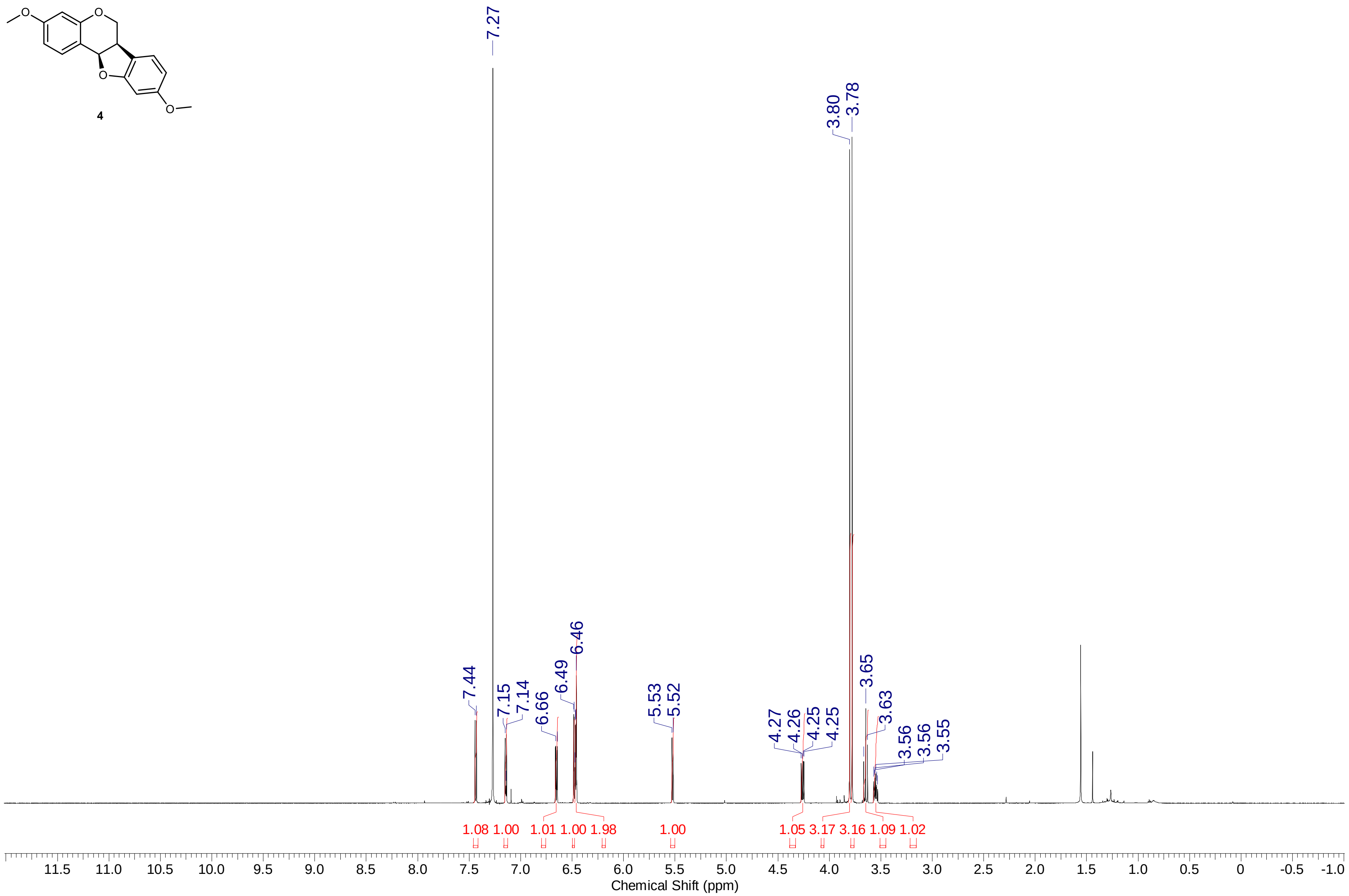

Supplementary Figure 27. ^{1}H NMR of **4**

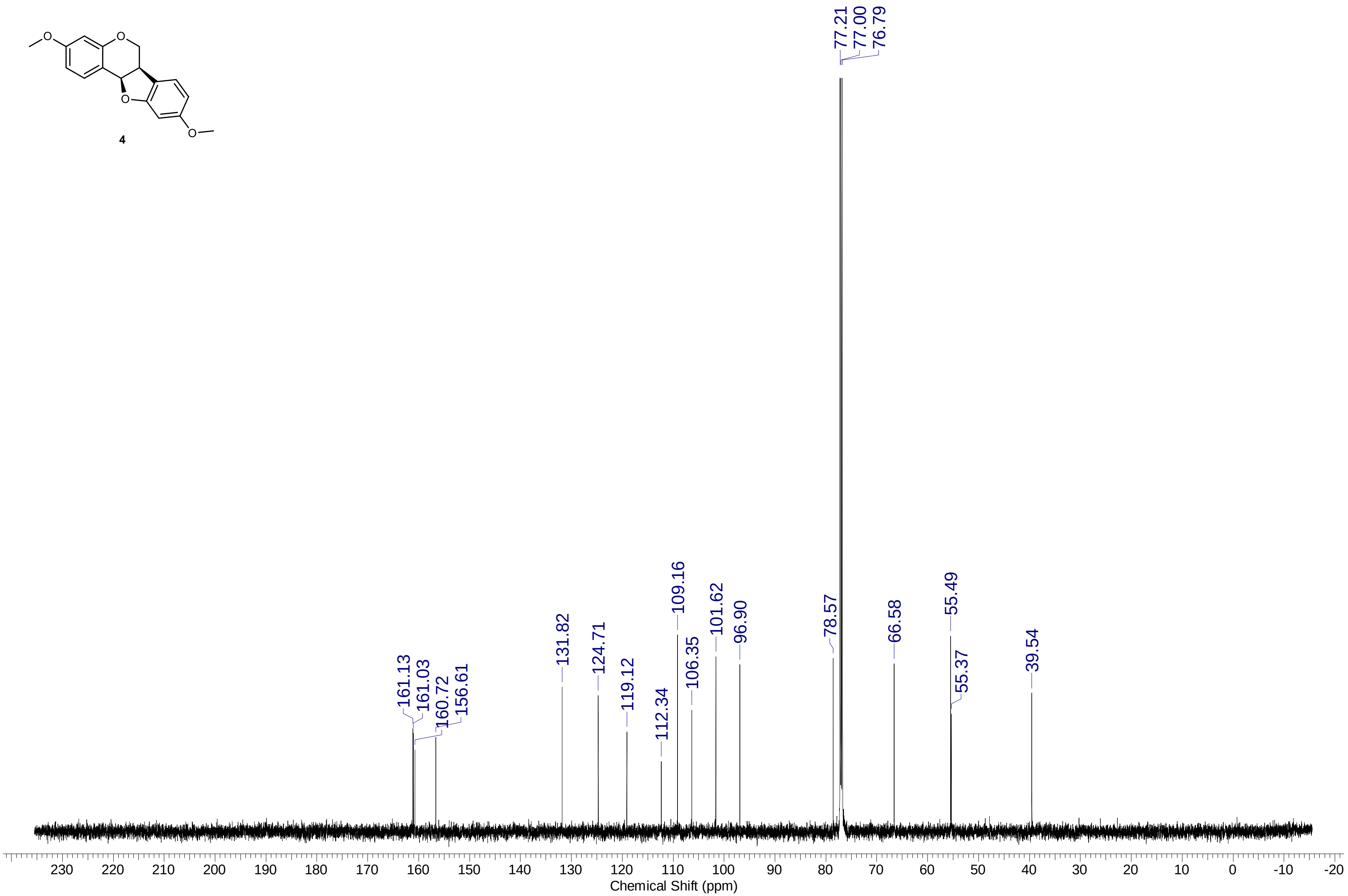

Supplementary Figure 28. ^{13}C NMR of **4**

Chiral HPLC trace of *rac*-**4** (bottom) and (*R*,*R*)-**4** (top, >99% *ee*); Method: Chiralpak® AD, hexane:isopropanol 90:10 *v/v*, 0.7 mL/min

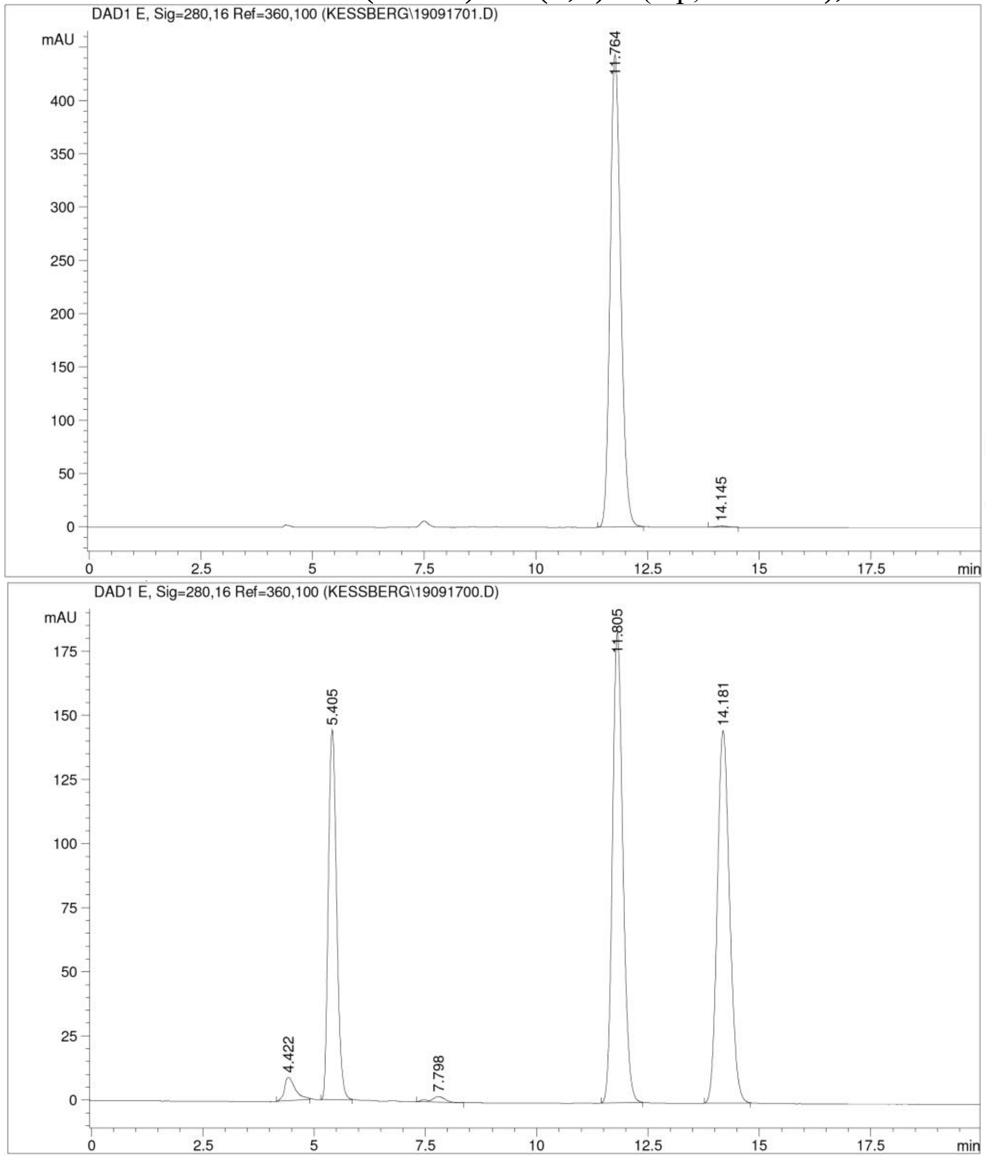

Peak #	RetTime [min]	Type	Width [min]	Area [mAU*s]	Height [mAU]	Area %
1	11.764	BB	0.2544	7314.91895	443.58646	99.6756
2	14.145	BB	0.2686	23.80468	1.29420	0.3244

Supplementary Figure 29. Chiral HPLC trace of **4**

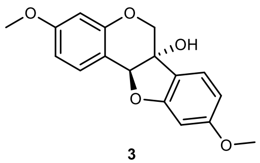

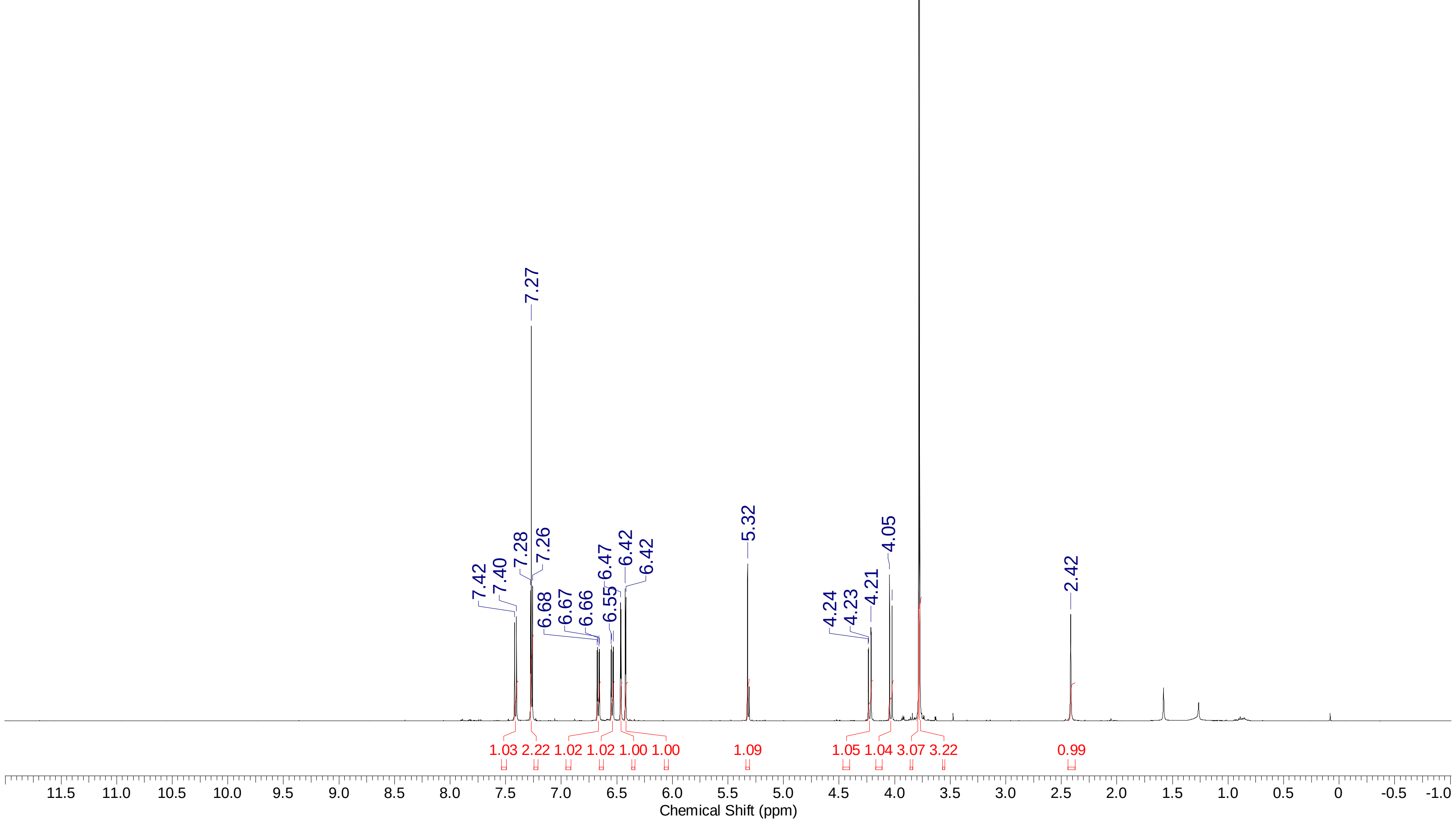

Supplementary Figure 30. ^{1}H NMR of **3**

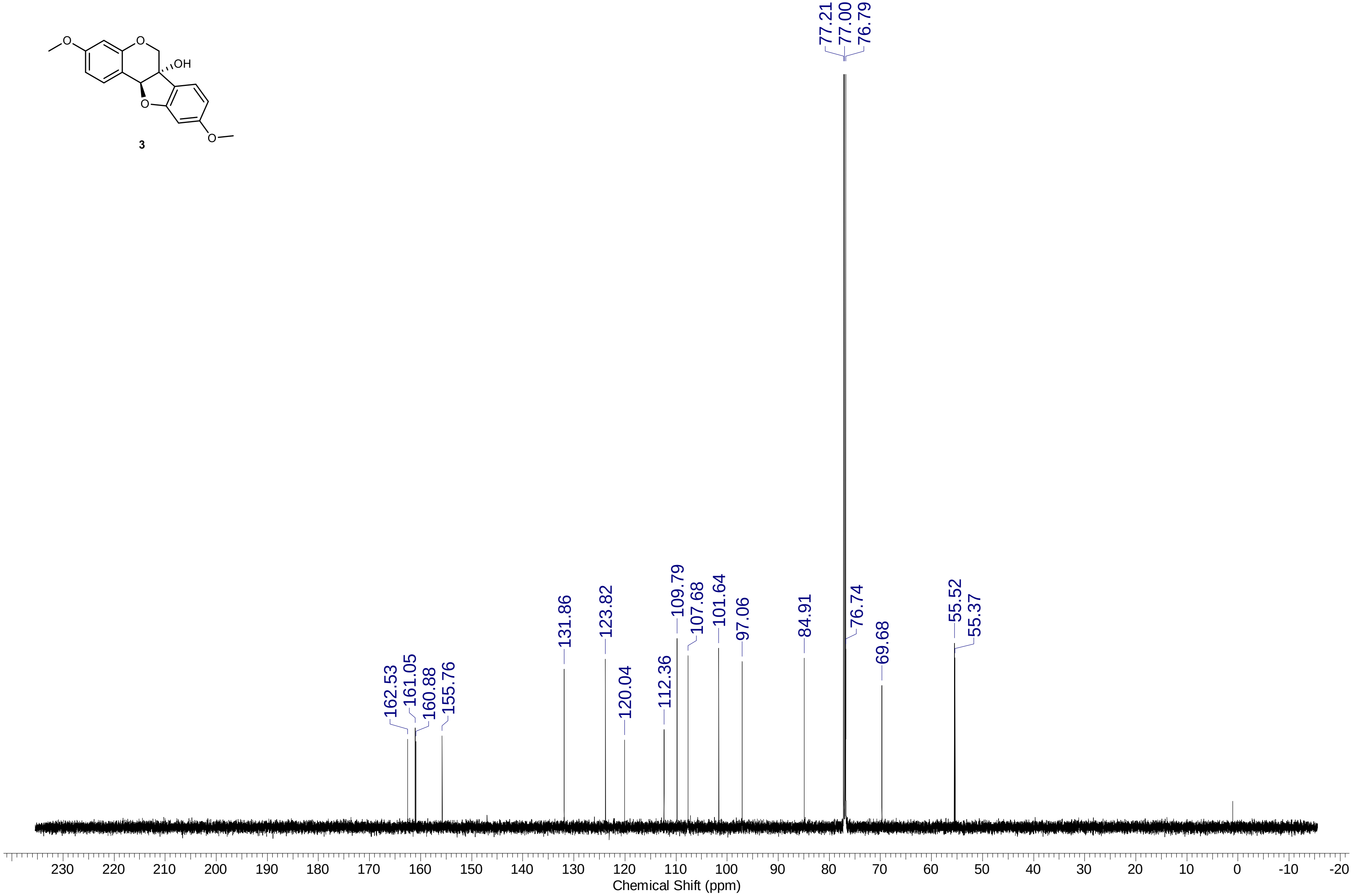

Supplementary Figure 31. ^{13}C NMR of **3**

Chiral HPLC trace of *rac*-**3** (bottom) and (*S*,*S*)-**3** (top, >99% *ee*); Method: Chiralpak® AD, hexane:isopropanol 80:20 *v/v*, 1.0 mL/min.

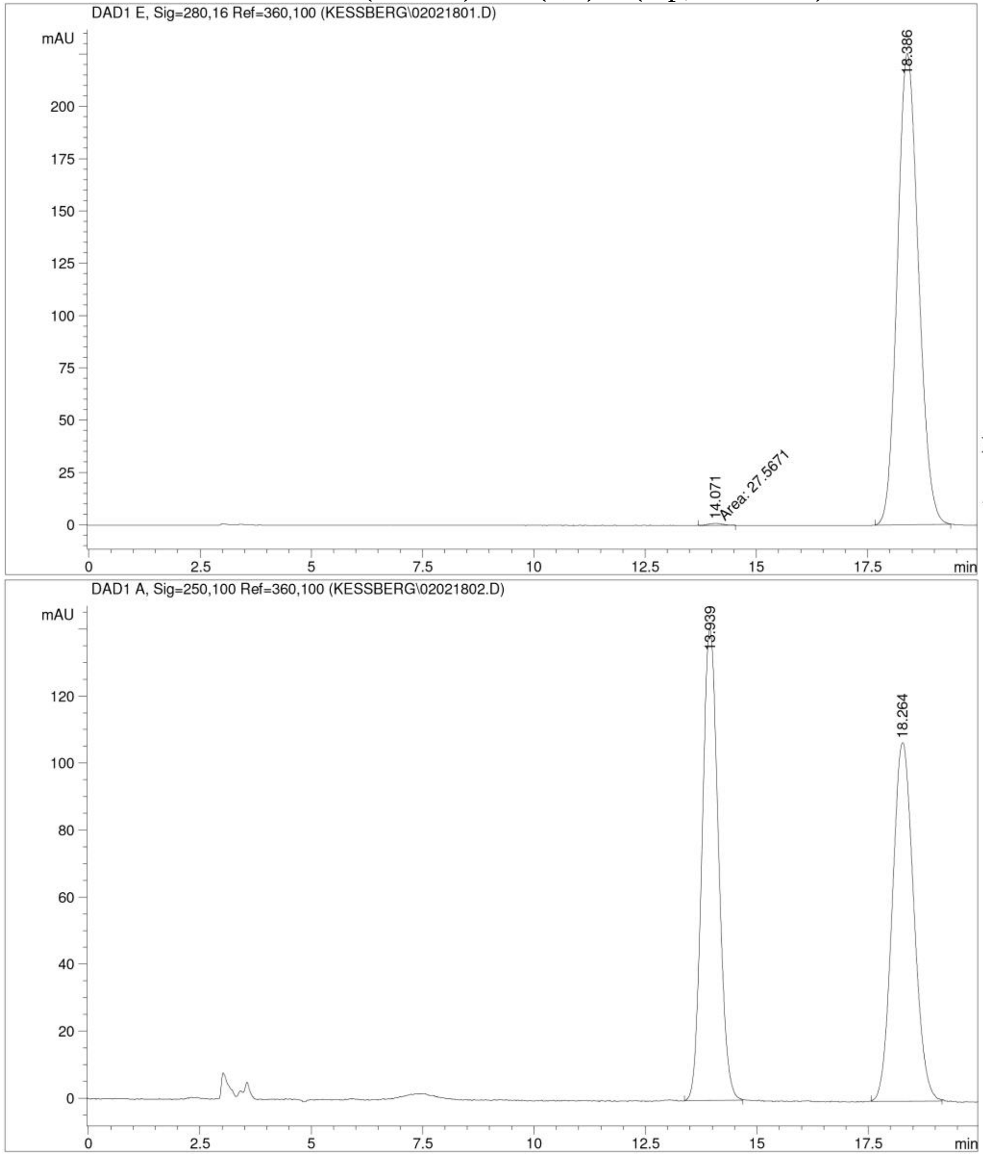

Peak #	RetTime [min]	Type	Width [min]	Area [mAU*s]	Height [mAU]	Area %
1	14.071	MM T	0.4328	27.56715	1.06162	0.3678
2	18.386	BB	0.5142	7467.69971	225.58810	99.6322

Supplementary Figure 32. Chiral HPLC trace of **3**

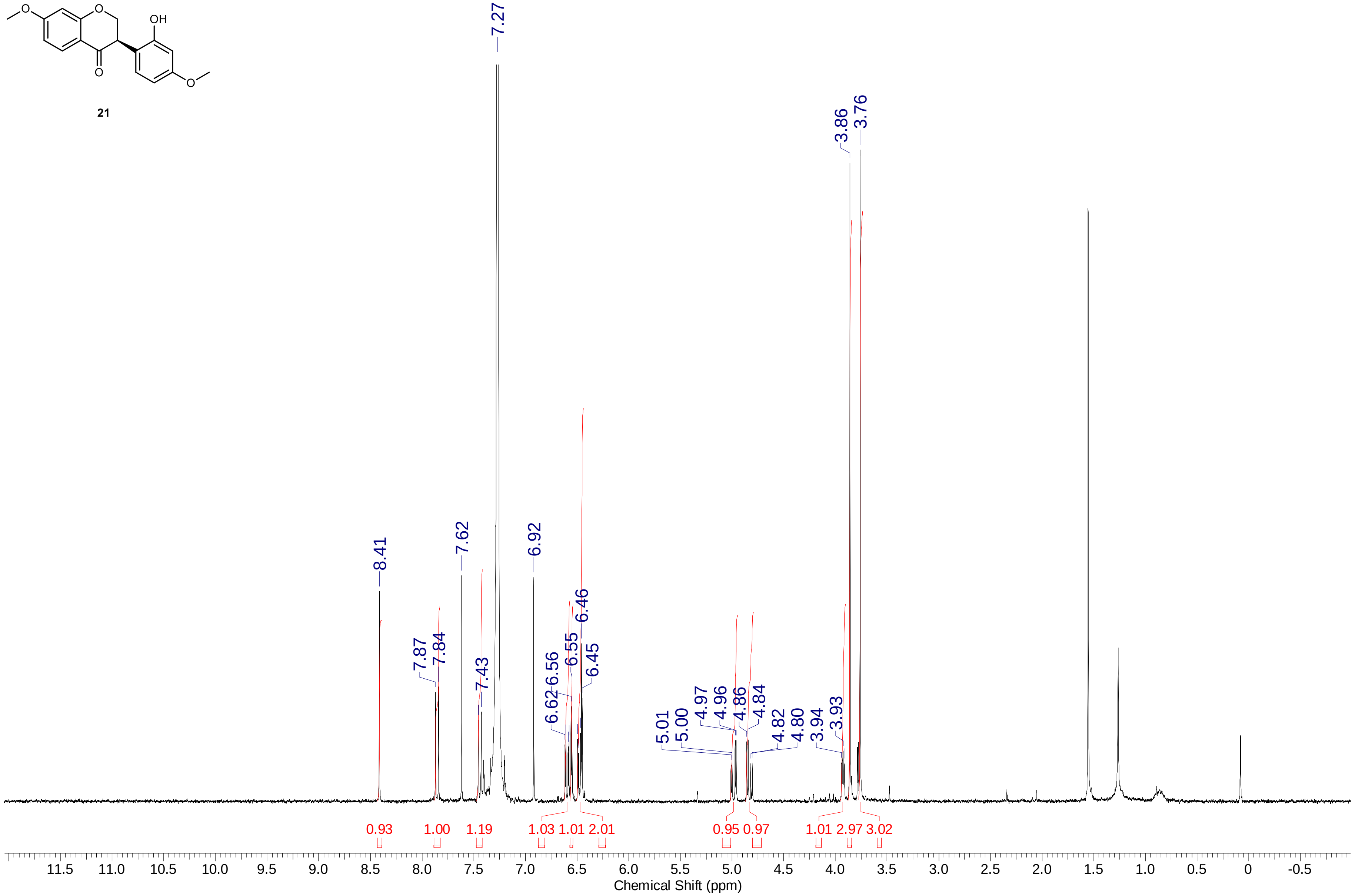

Supplementary Figure 33. ^{1}H NMR of **21**

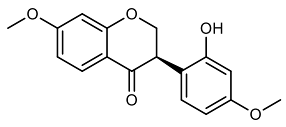

21

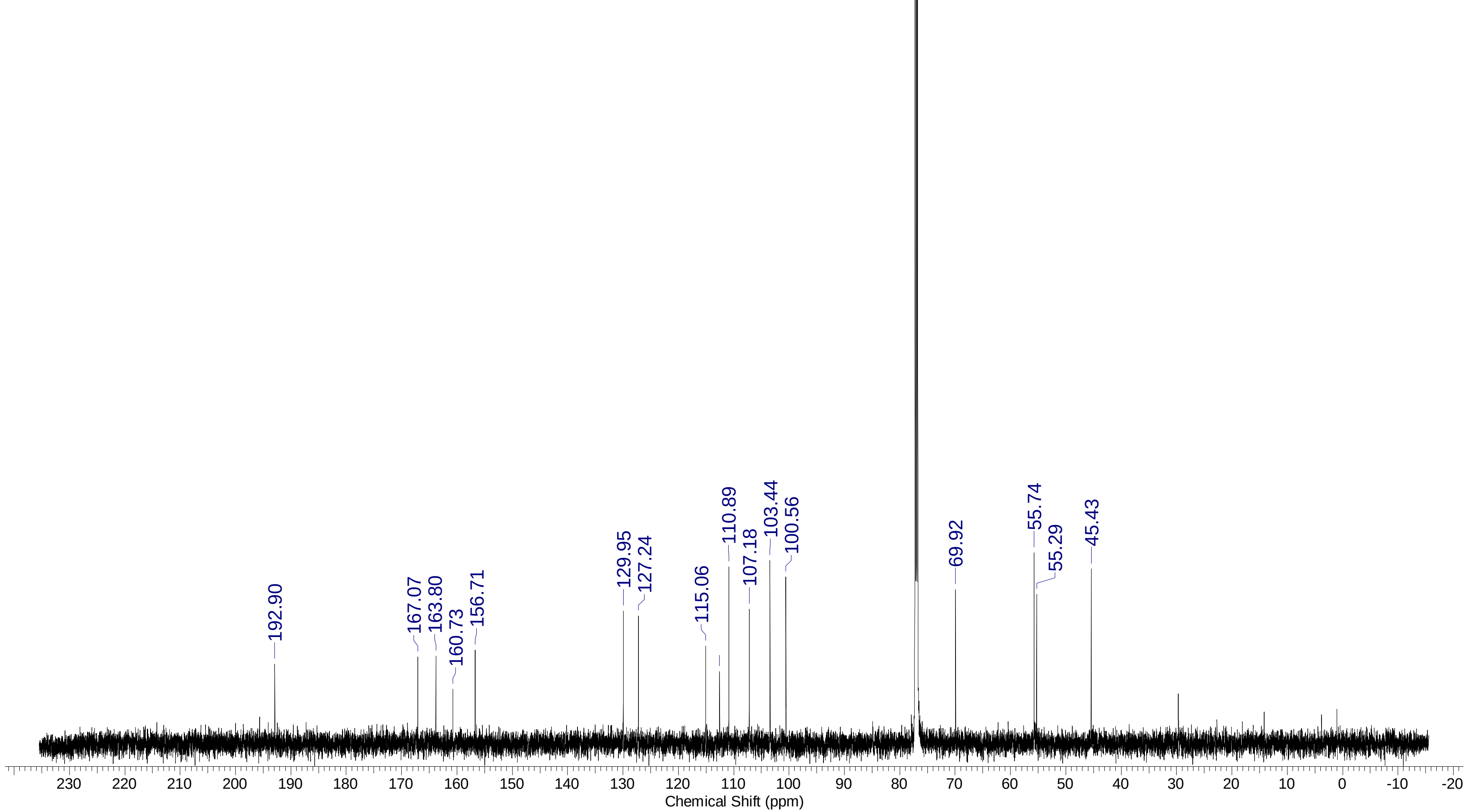

Supplementary Figure 34. ^{13}C NMR of **21**

Chiral HPLC trace of *rac*-**21** (bottom) and (*R*)-**21** (top, 91% *ee*); Method: Chiralpak® AD, hexane:isopropanol 80:20 *v/v*, 1.0 mL/min

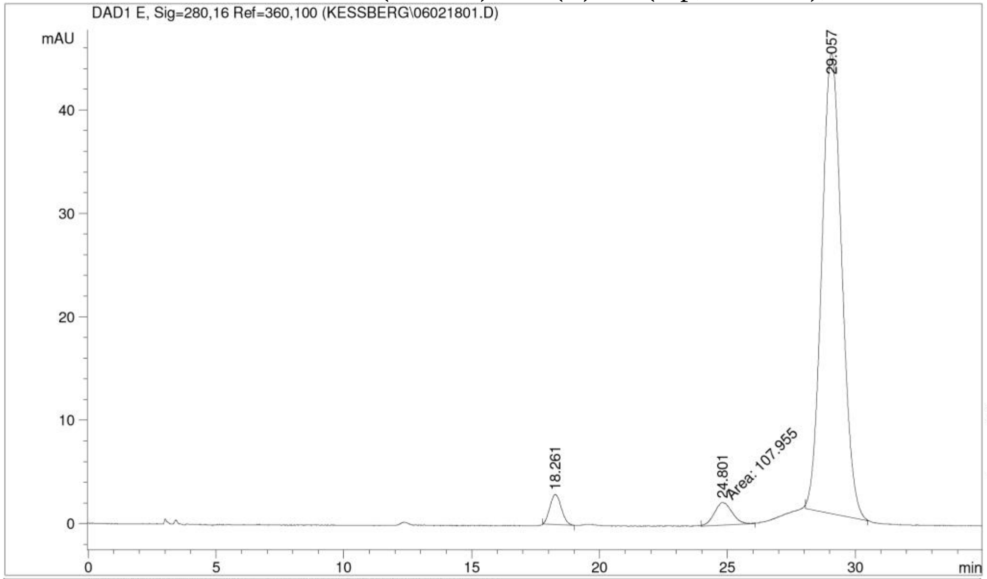

Peak #	RetTime [min]	Type	Width [min]	Area [mAU*s]	Height [mAU]	Area %
1	18.261	BP	0.3763	90.29533	2.90750	3.4375
2	24.801	MM T	0.8223	107.95507	2.18807	4.1098
3	29.057	BB	0.8325	2428.50171	44.51419	92.4526

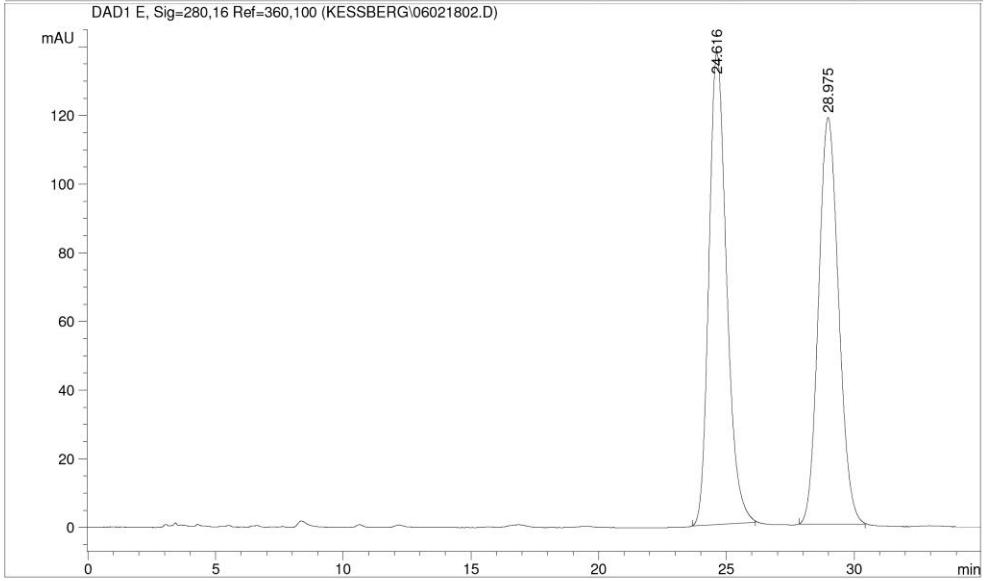

Supplementary Figure 35. Chiral HPLC trace of **21**

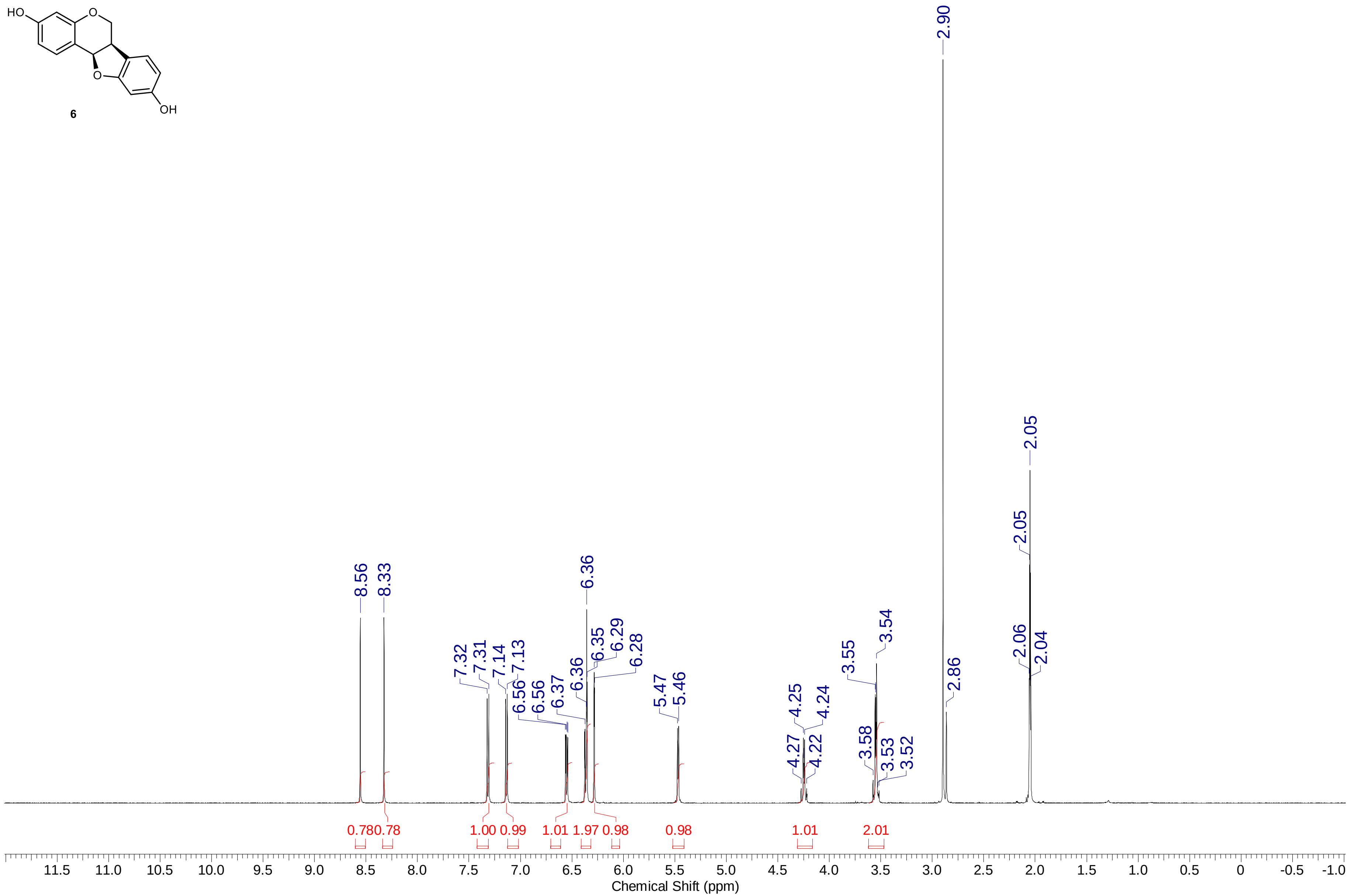

Supplementary Figure 36. ^{1}H NMR of **6**

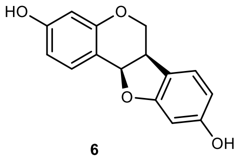

6

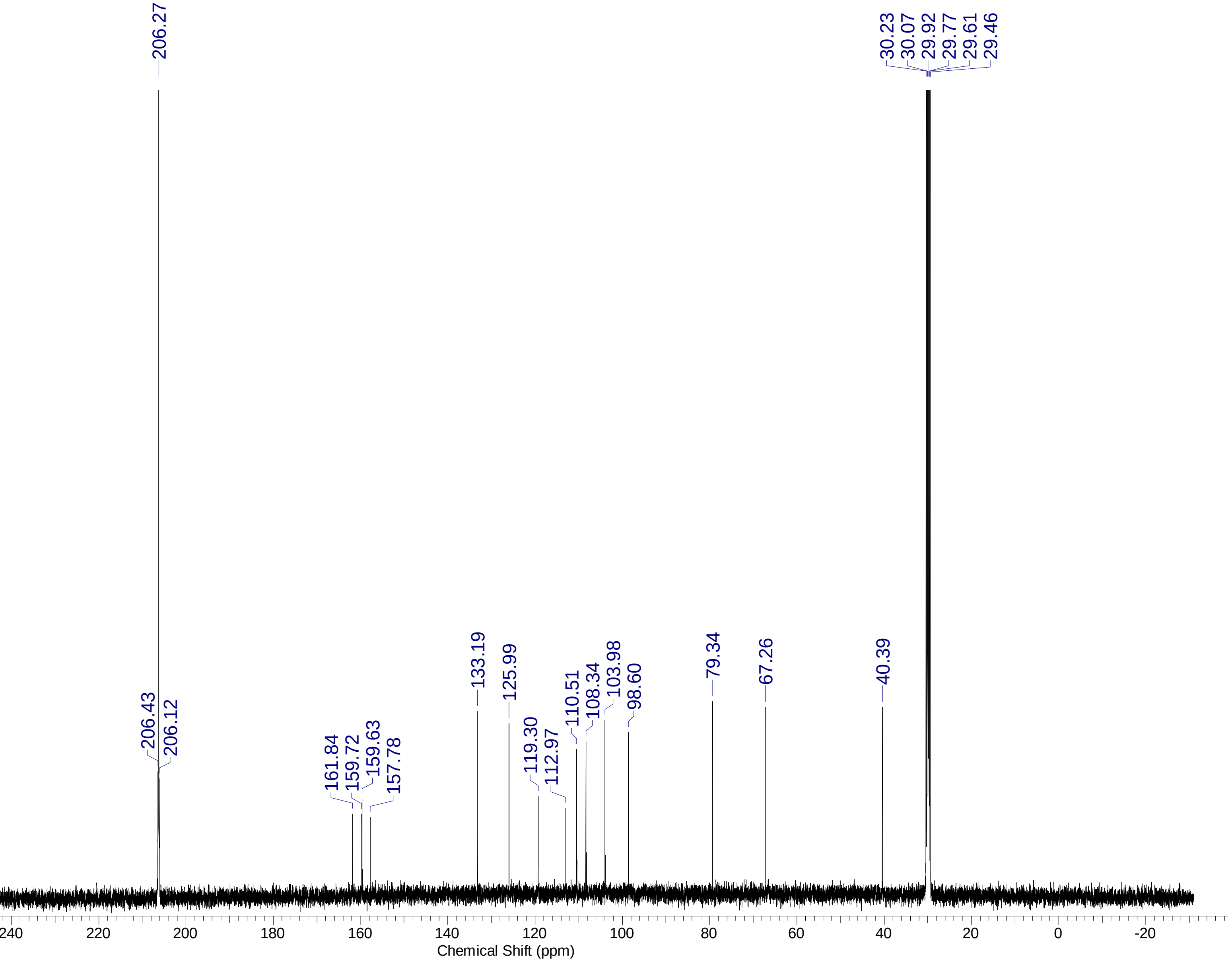

Supplementary Figure 37. ^{13}C NMR of **6**

Chiral HPLC trace of *rac*-**6** (bottom) and (*R*,*R*)-**6** (top, 99% *ee*); Method: Lux® Cellulose-1, hexane:isopropanol 75:25 *v/v*, 0.8 mL/min

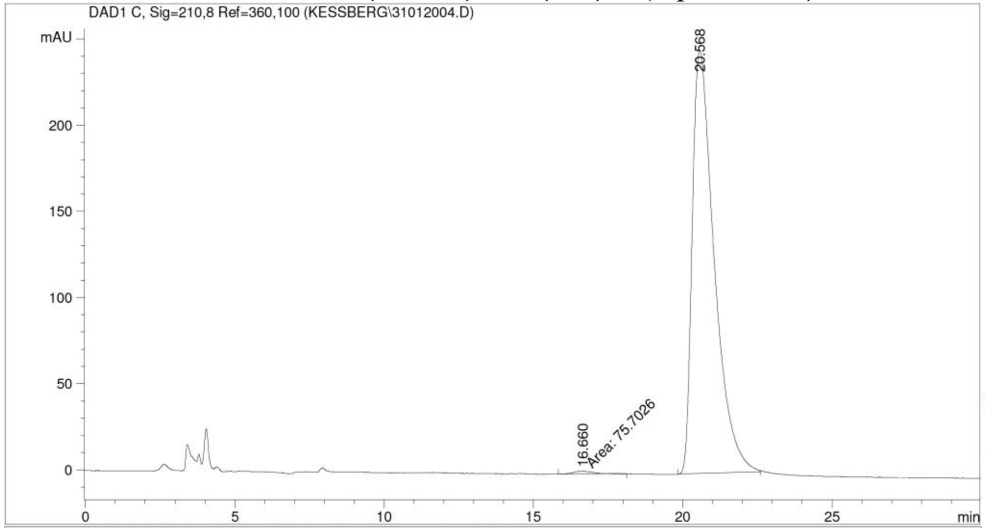

Peak #	RetTime [min]	Type	Width [min]	Area [mAU*s]	Height [mAU]	Area %
1	16.660	MM T	0.7617	75.70261	1.65652	0.5988
2	20.568	PB	0.7544	1.25674e4	245.83195	99.4012

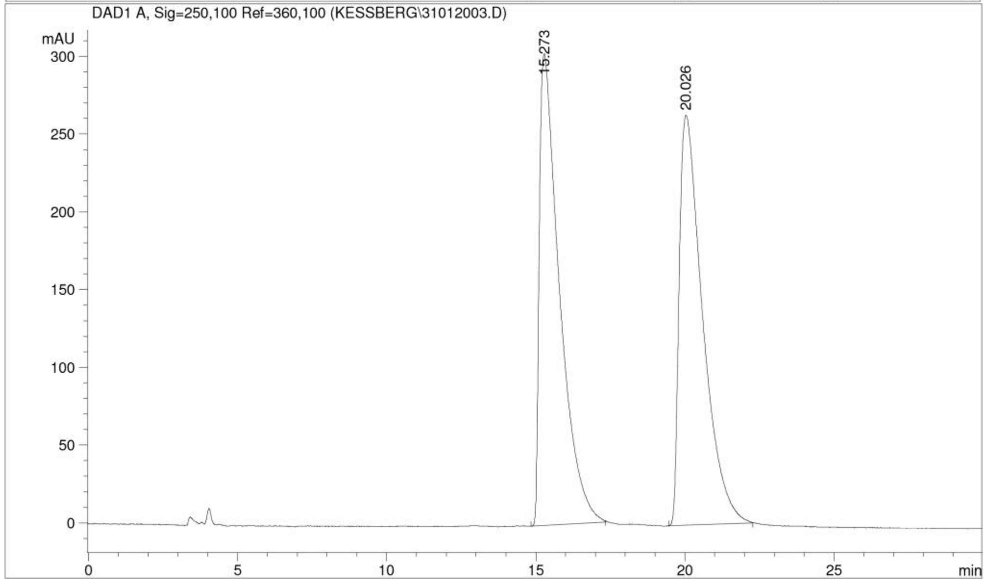

Supplementary Figure 38. Chiral HPLC trace of **6**

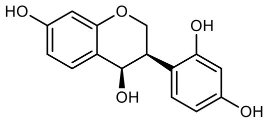

29

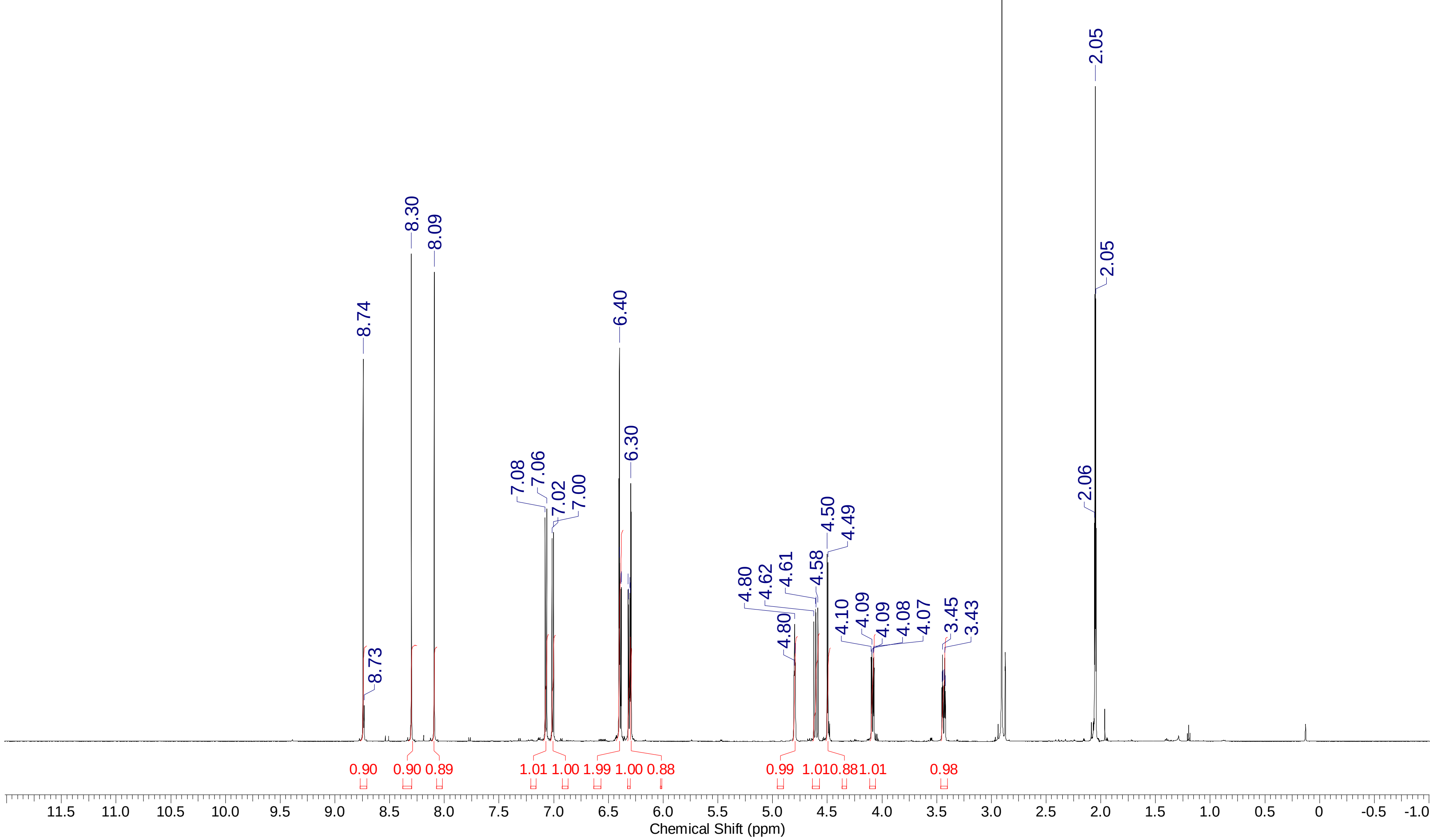

Supplementary Figure 39. ^{1}H NMR of **29**

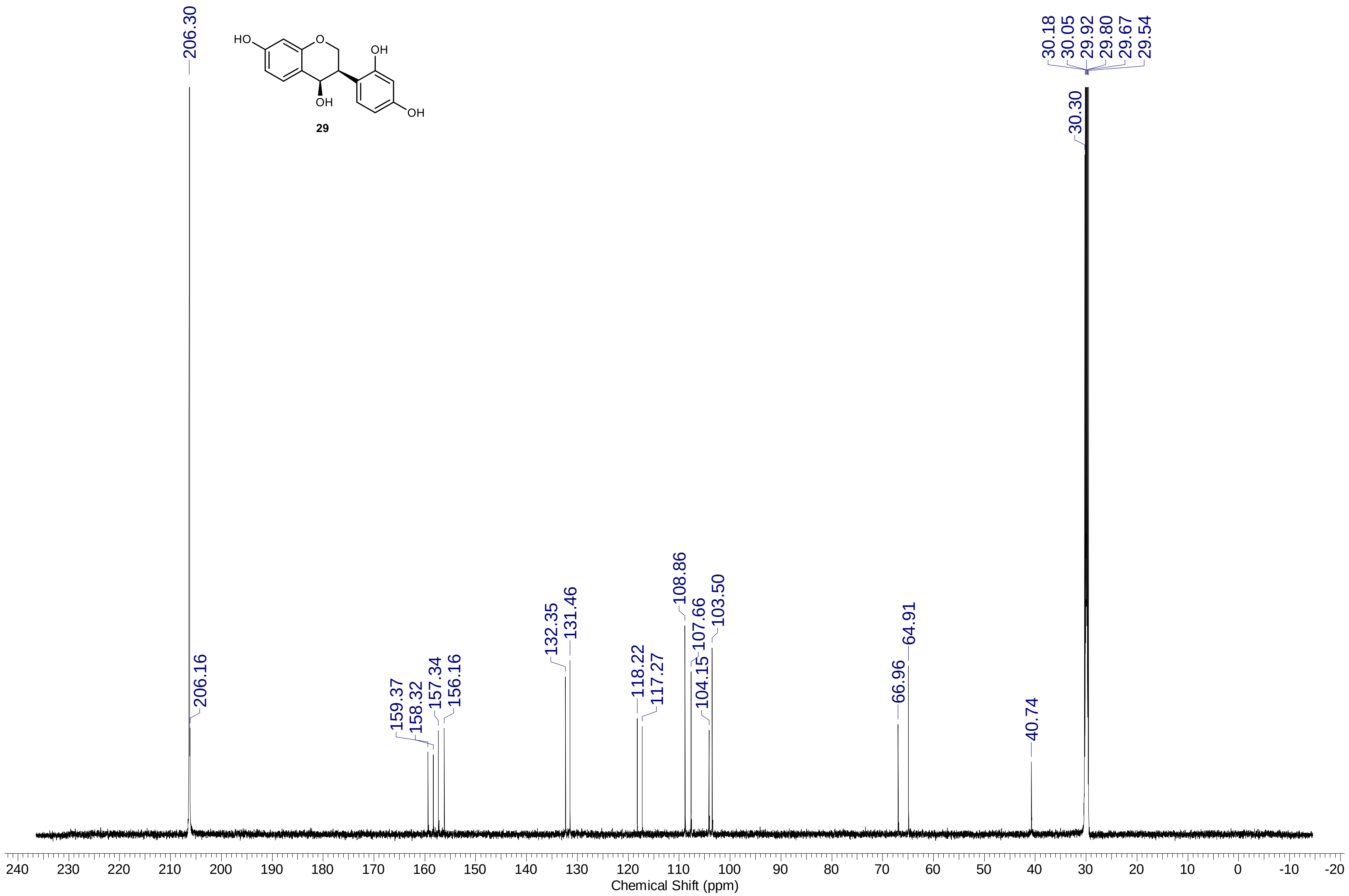

Supplementary Figure 40. ^{13}C NMR of **29**

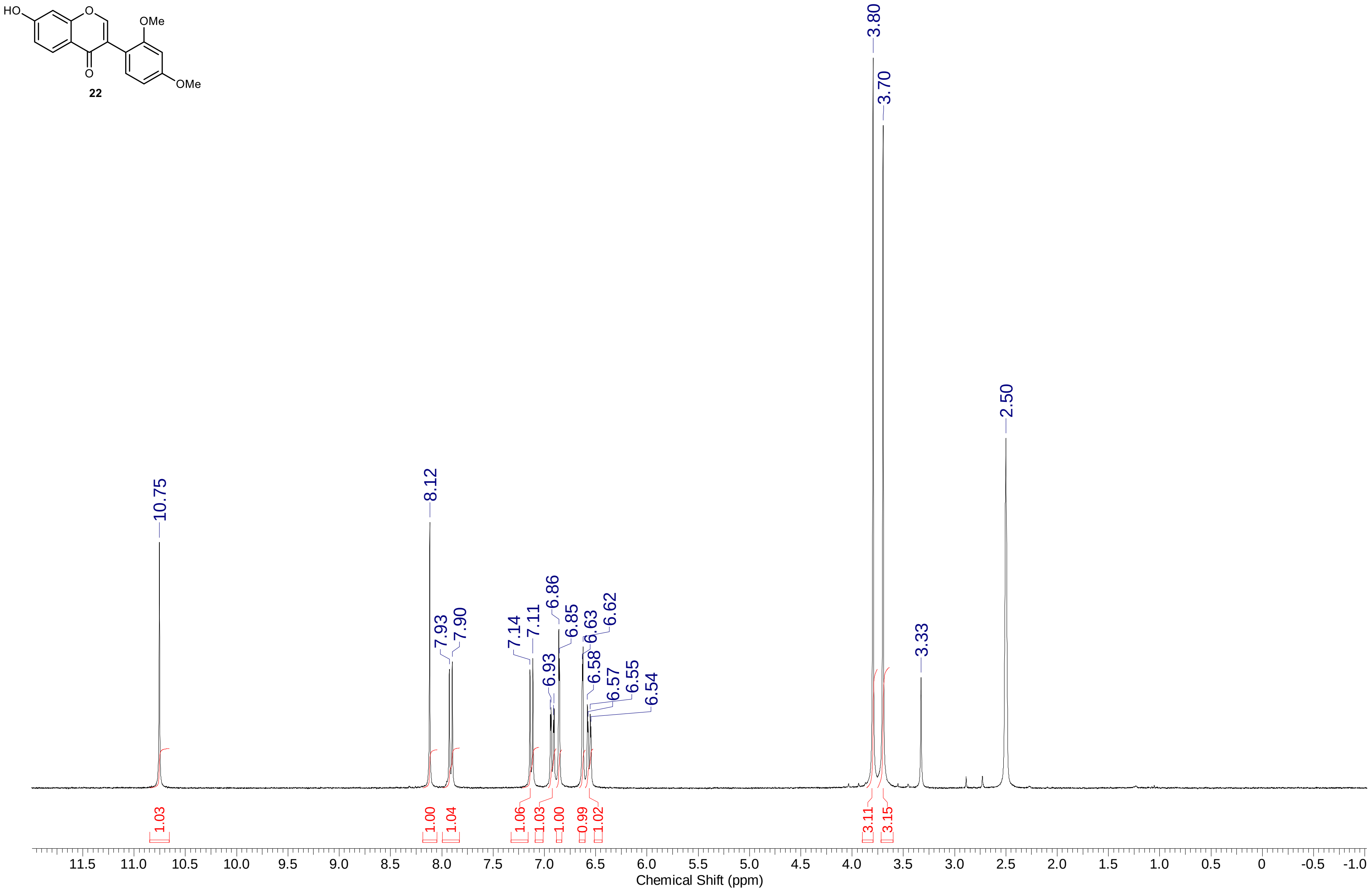

Supplementary Figure 41. ^{1}H NMR of **22**

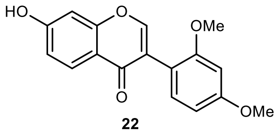

22

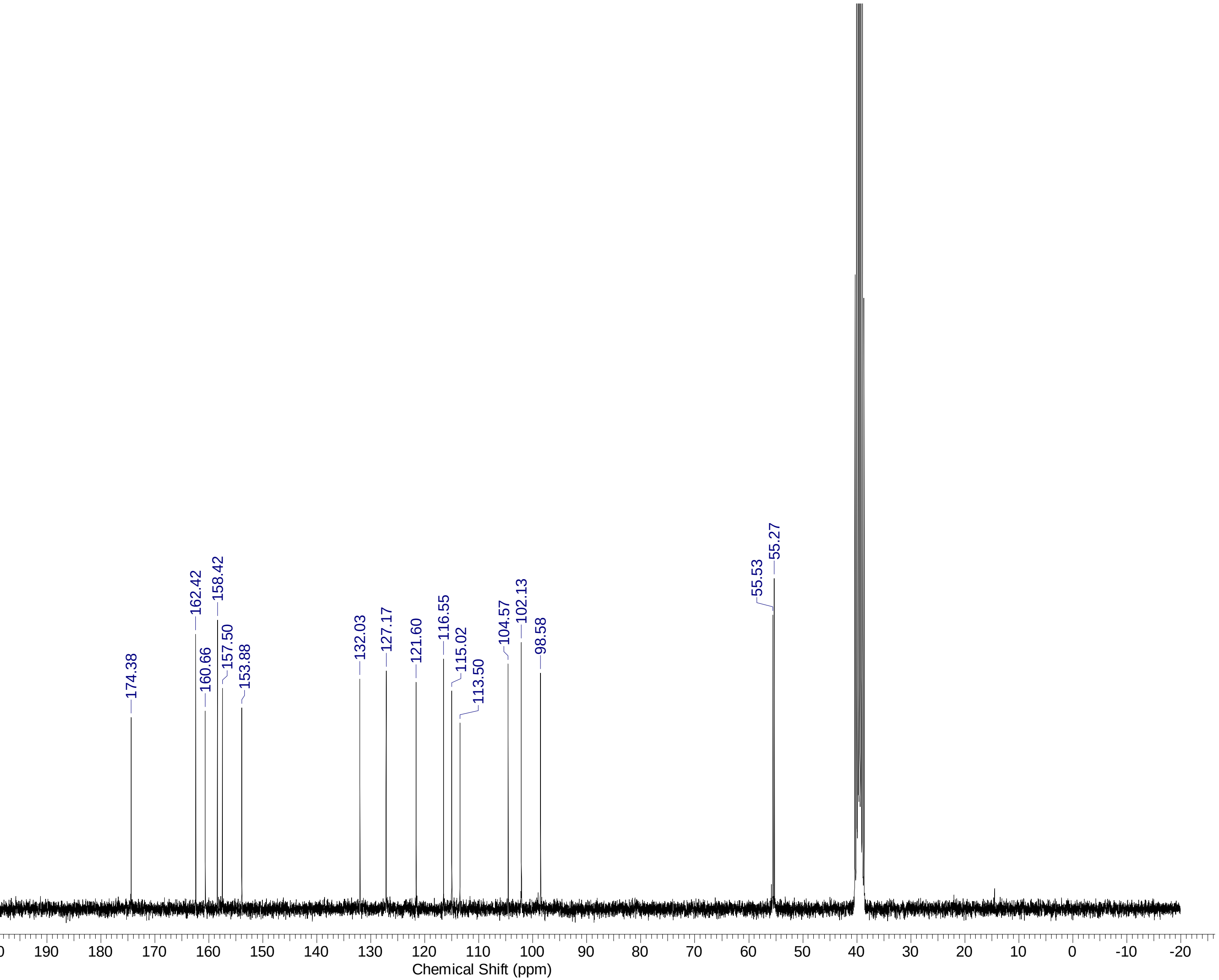

Supplementary Figure 42. ^{13}C NMR of **22**

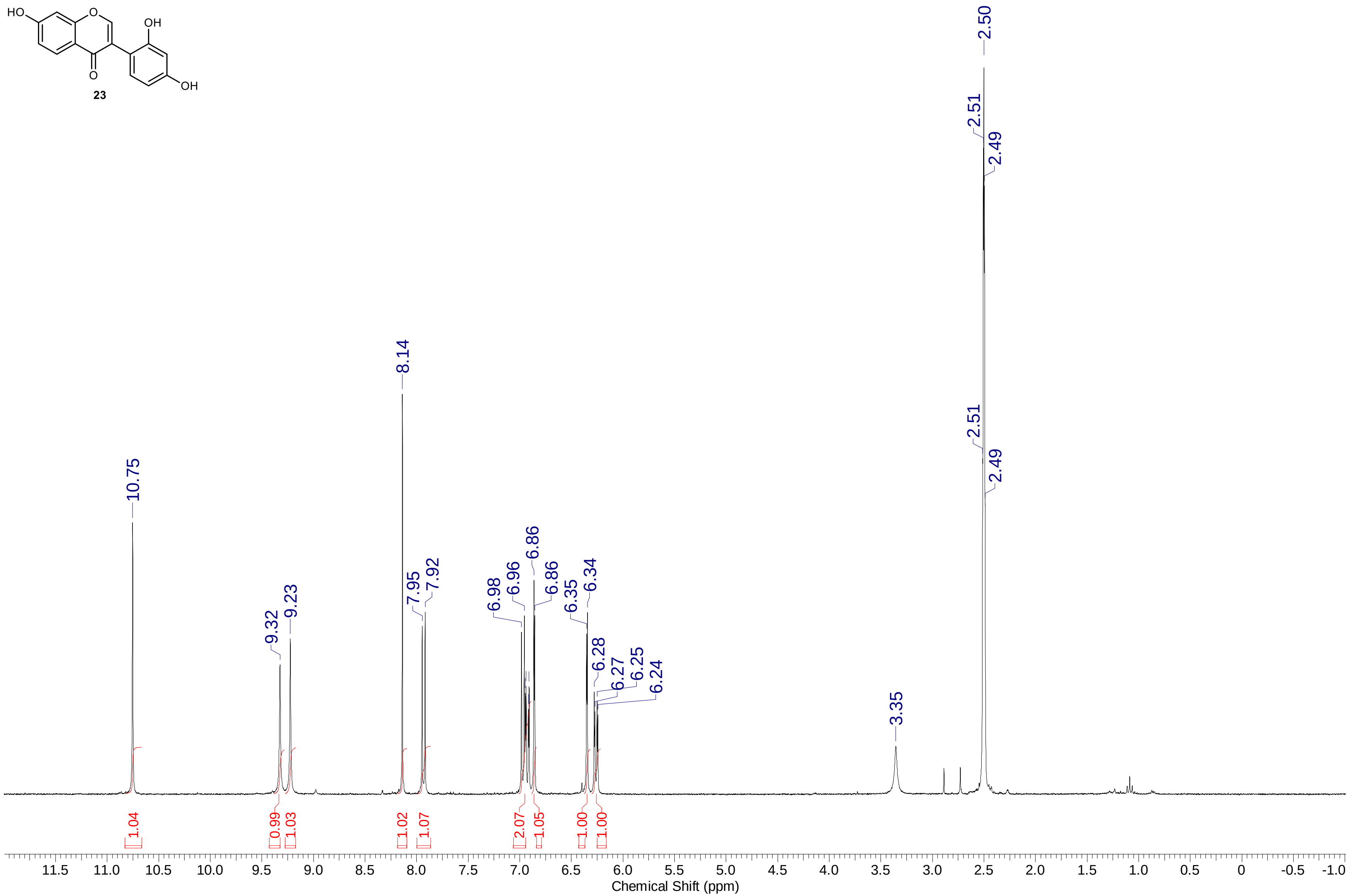

Supplementary Figure 43. ^{1}H NMR of **23**

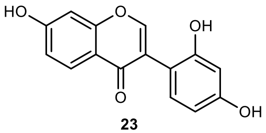

23

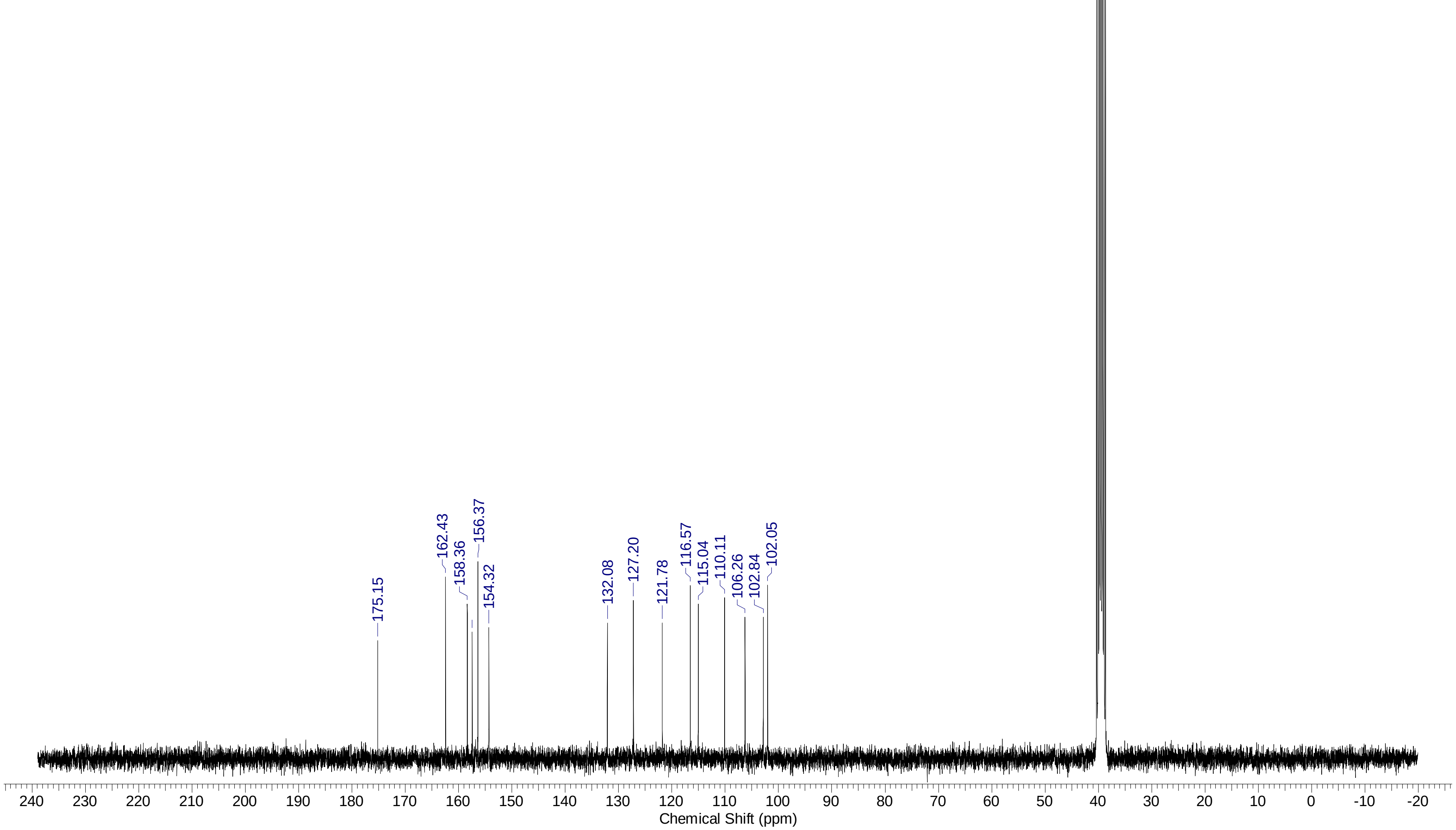

Supplementary Figure 44. ^{13}C NMR of **23**

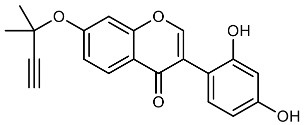

13

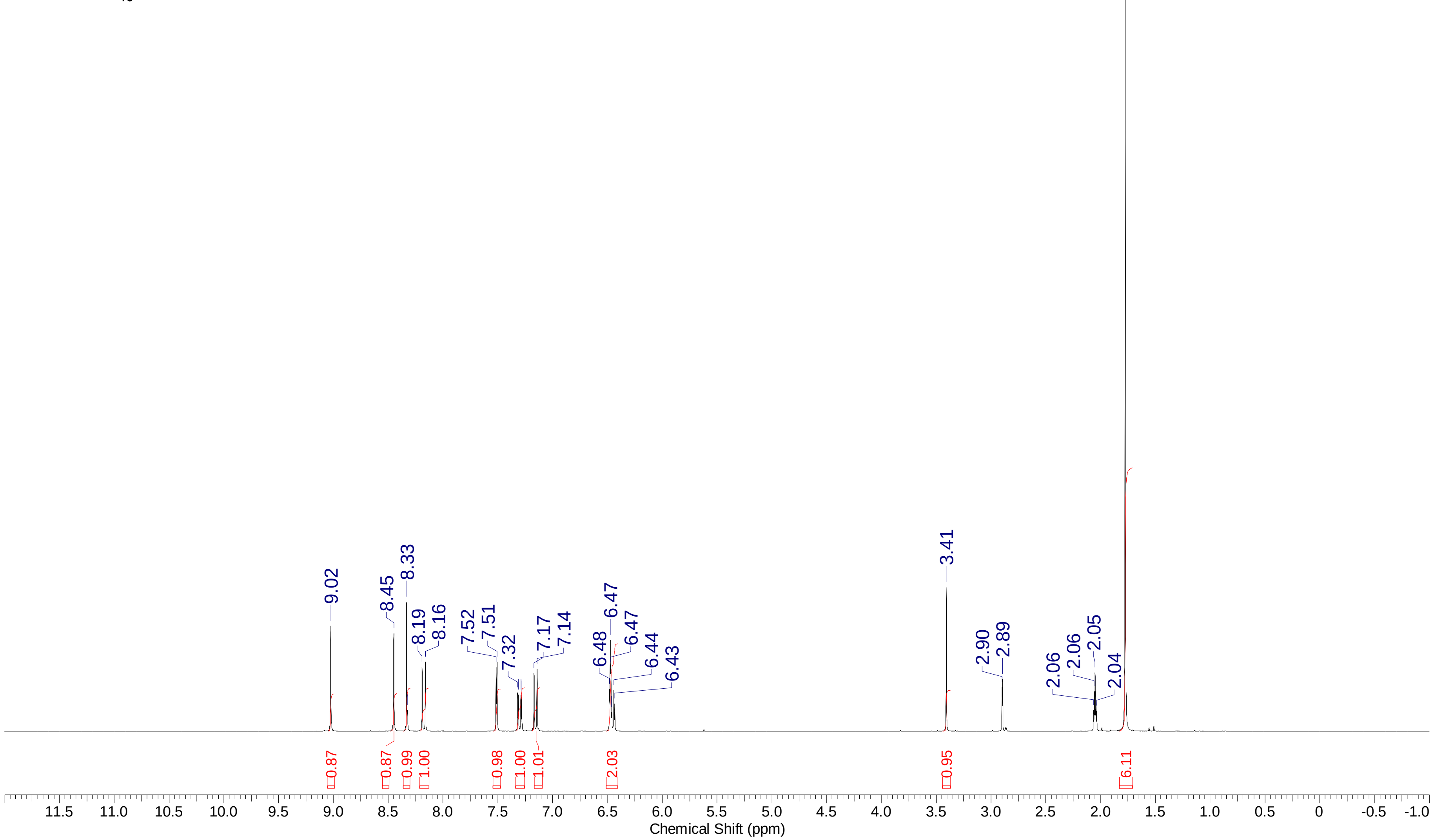

Supplementary Figure 45. ^{1}H NMR of **13**

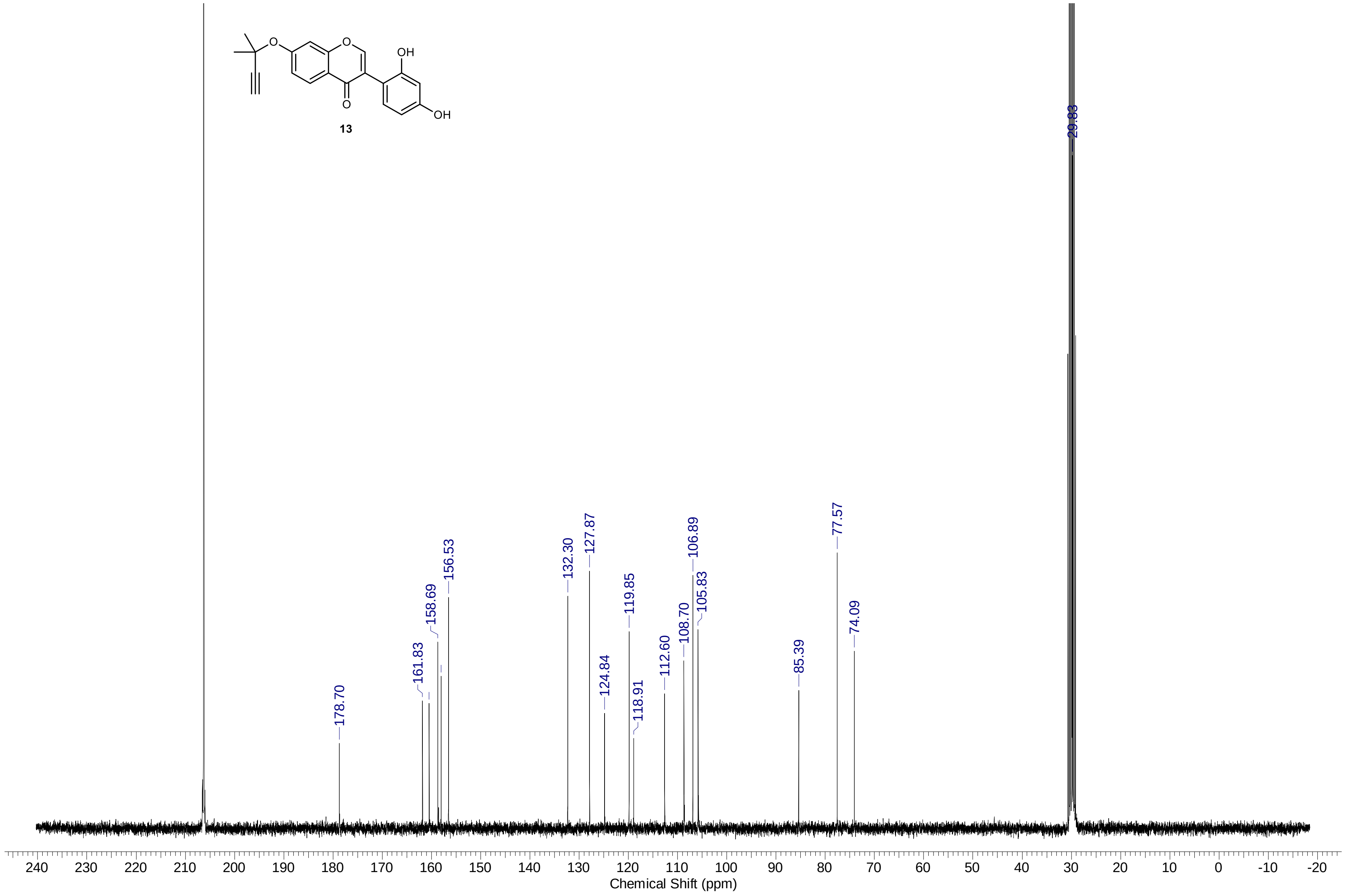

Supplementary Figure 46. ^{13}C NMR of **13**

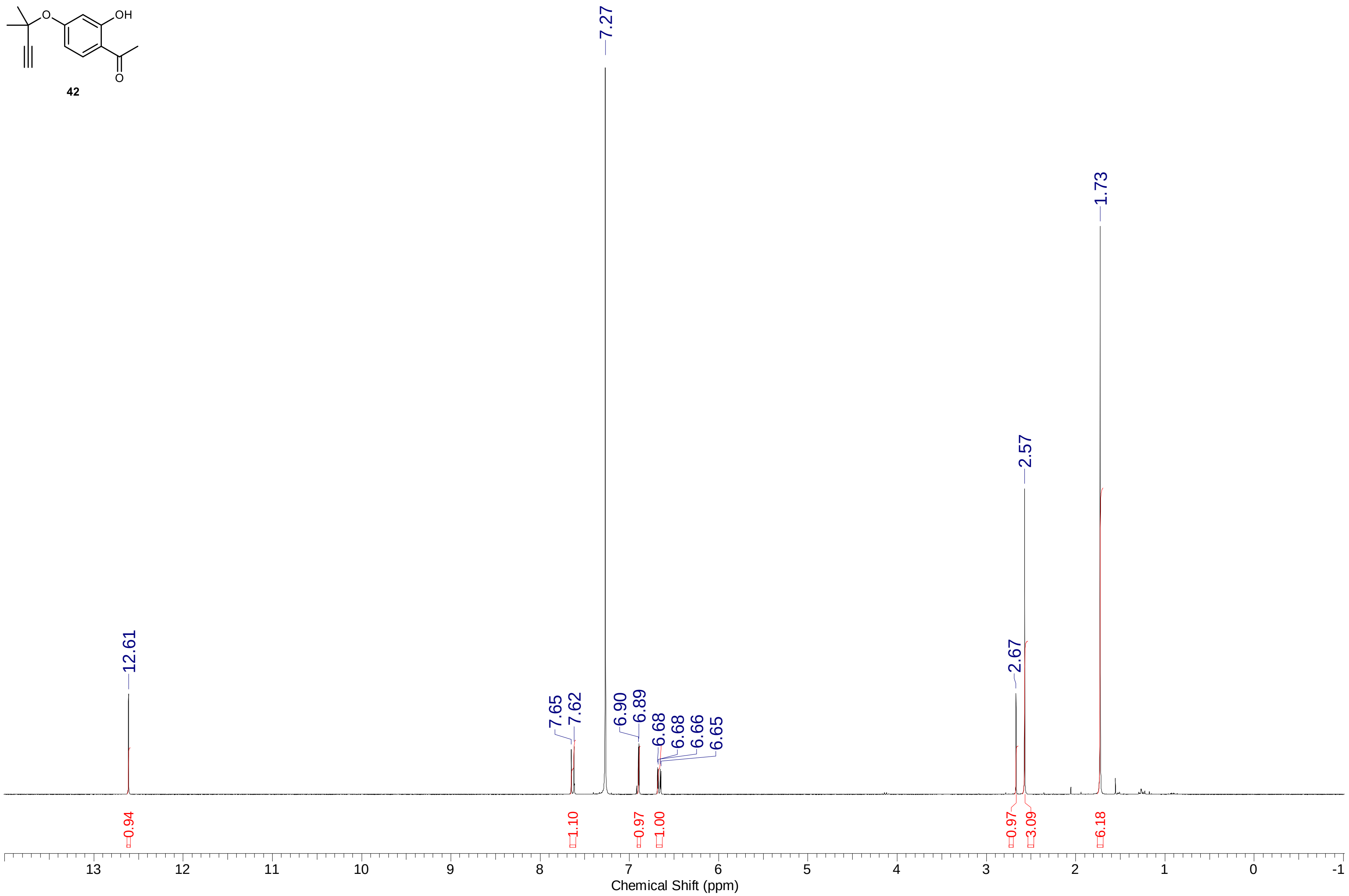

Supplementary Figure 47. ^{1}H NMR of **42**

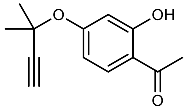

42

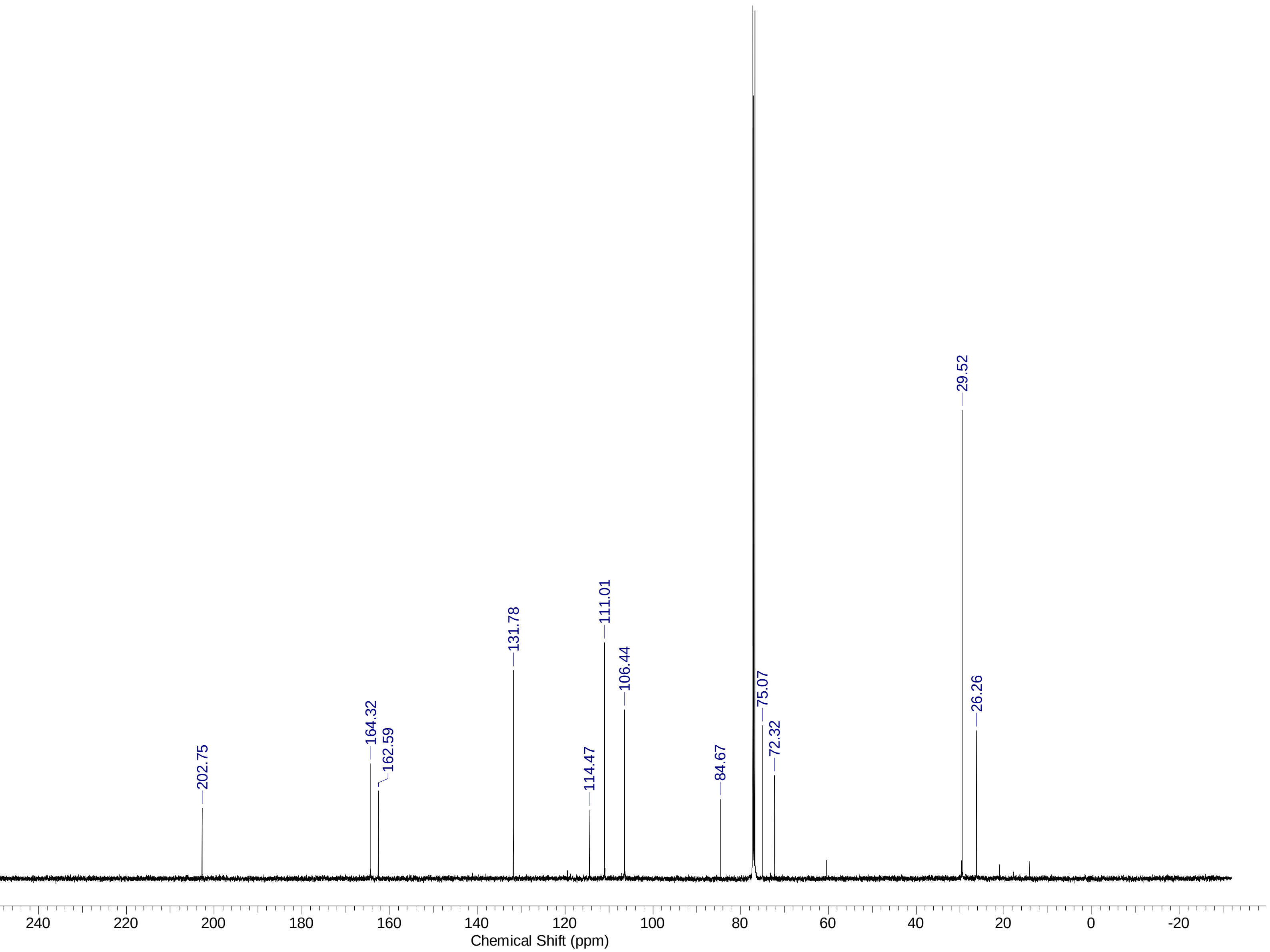

Supplementary Figure 48. ^{13}C NMR of **42**

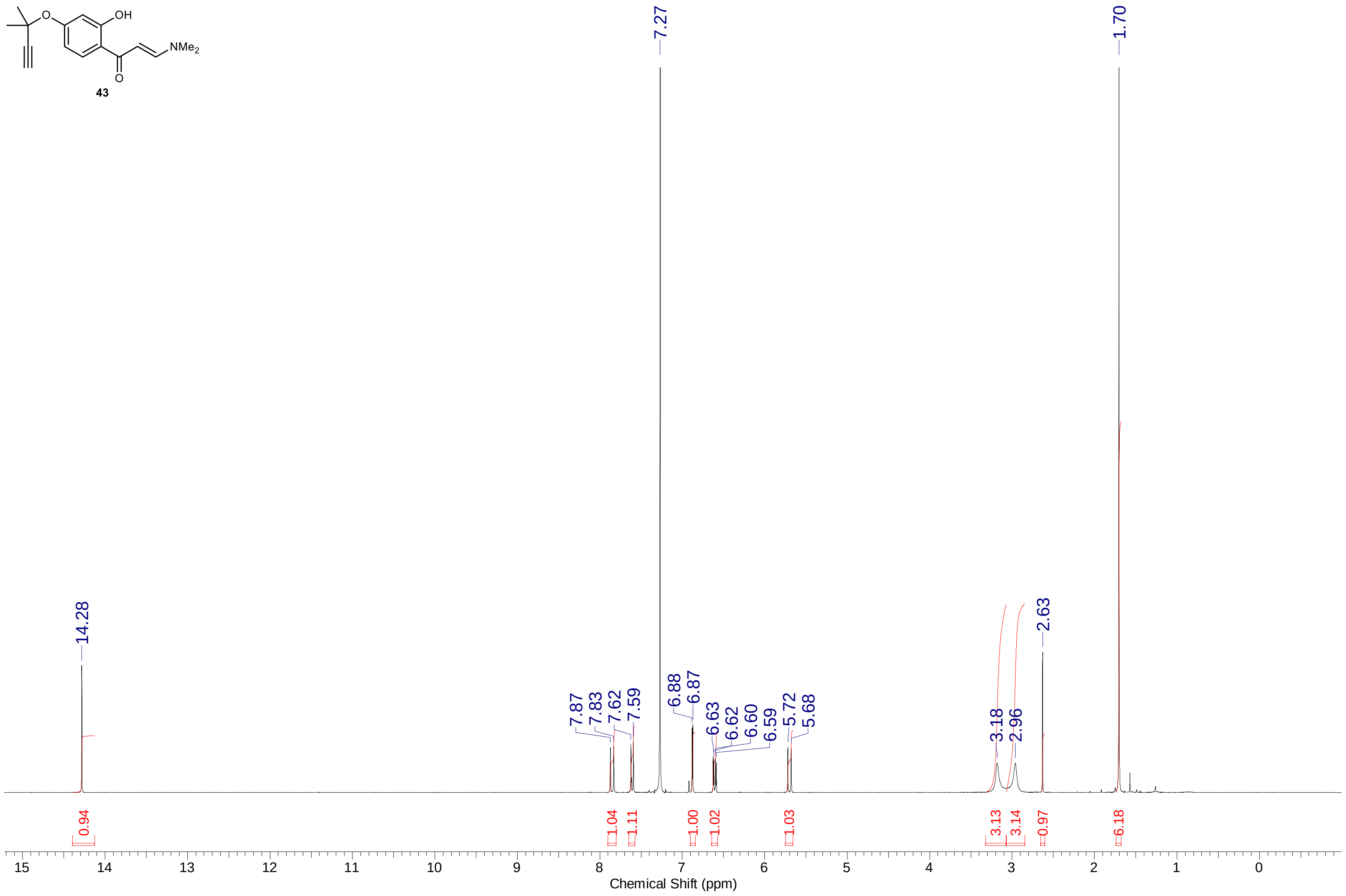

Supplementary Figure 49. ¹H NMR of **43**

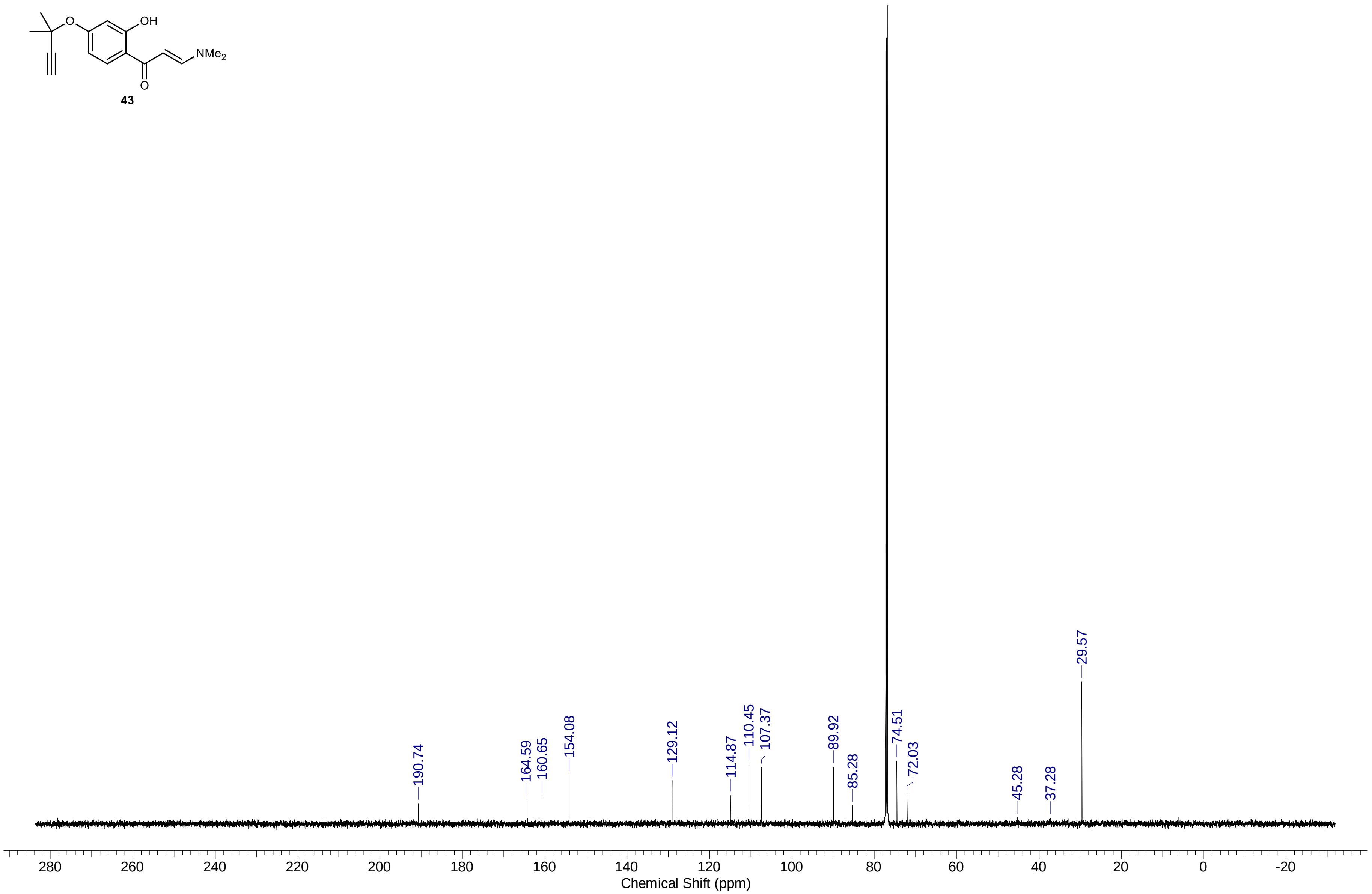

Supplementary Figure 50. ^{13}C NMR of **43**

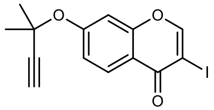

36

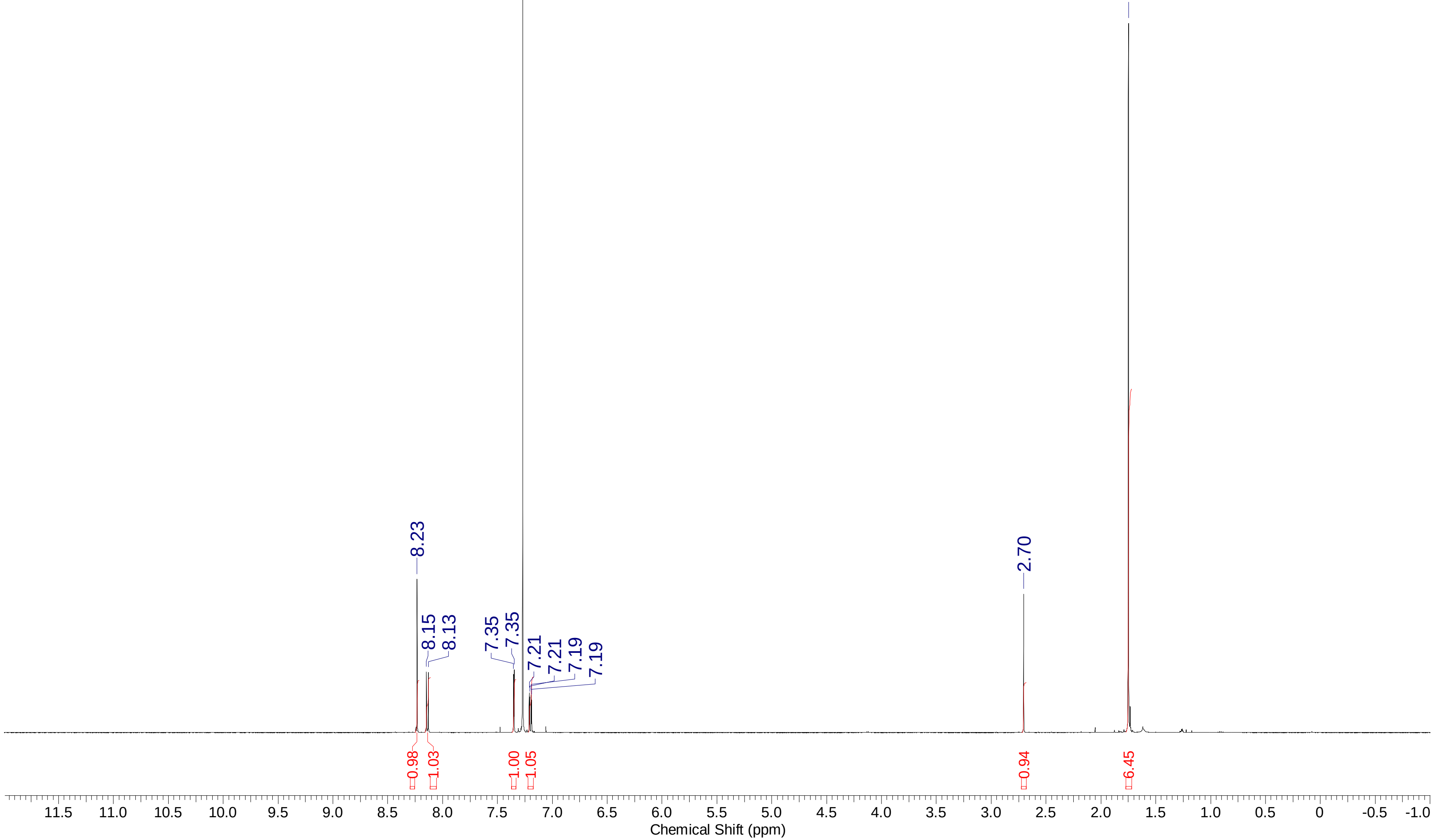

Supplementary Figure 51. ^{1}H NMR of **36**

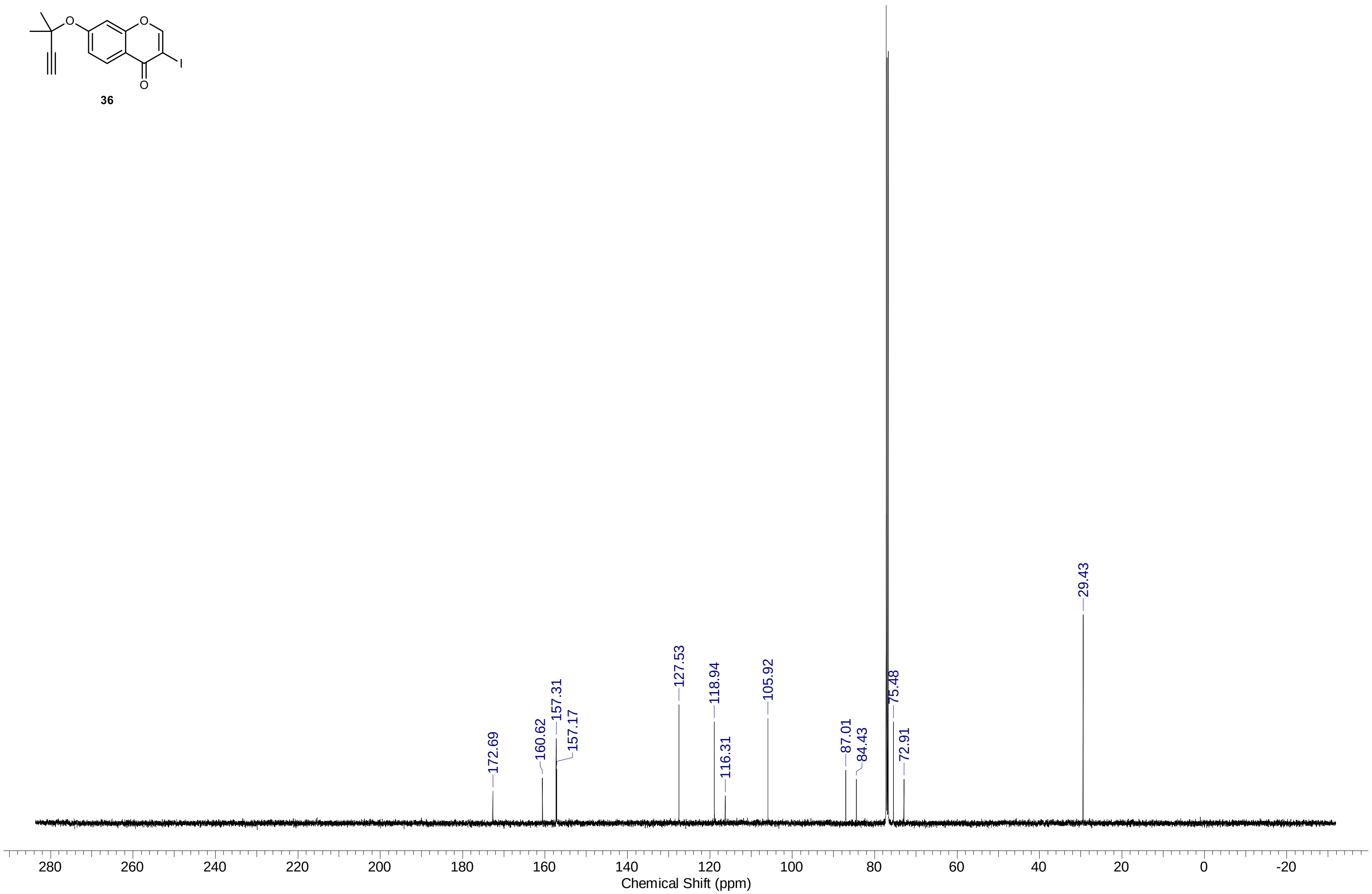

Supplementary Figure 52. ^{13}C NMR of **36**

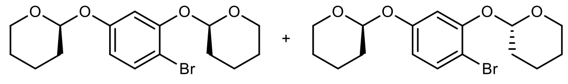

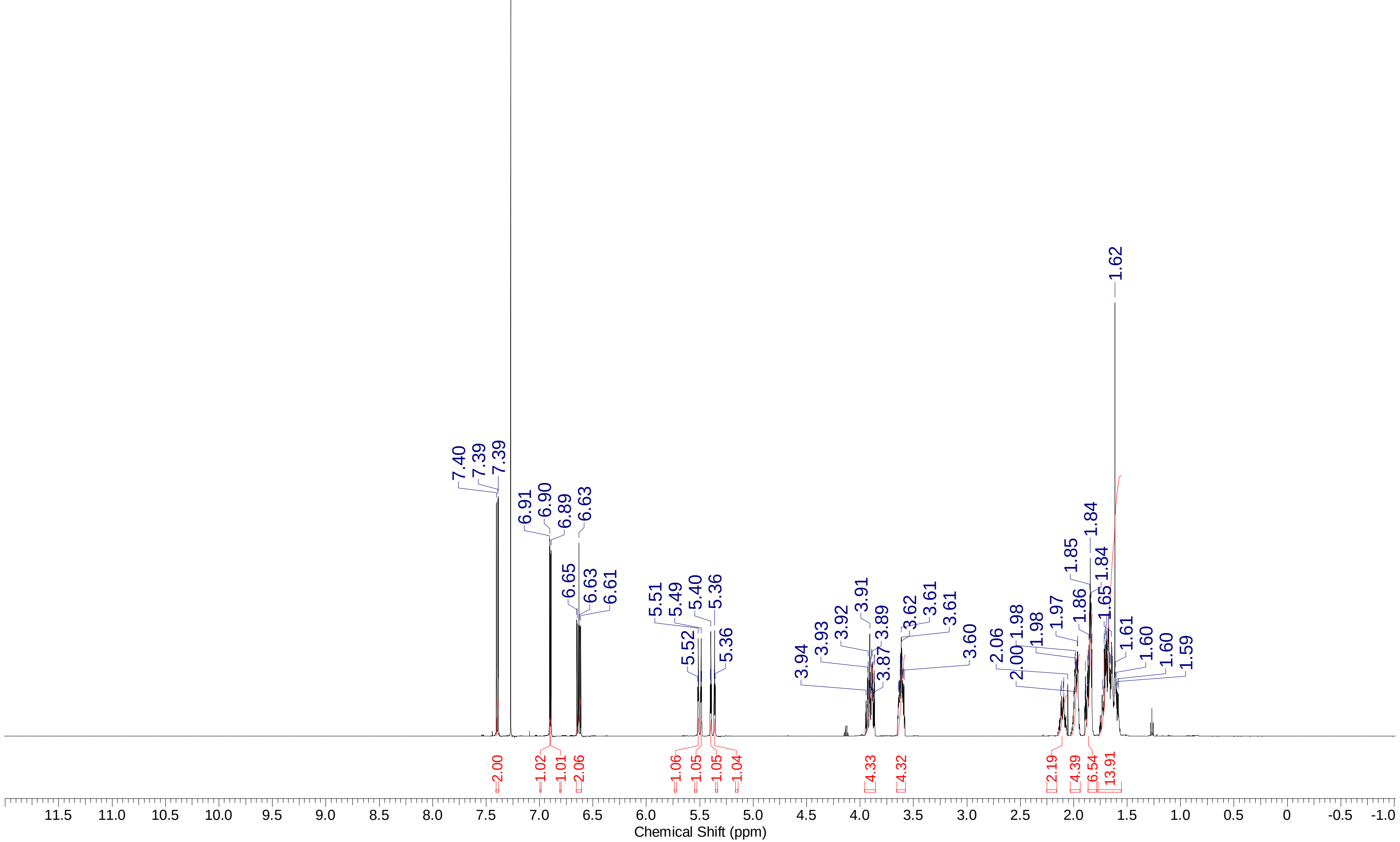

Supplementary Figure 53. ^{1}H NMR of **37**

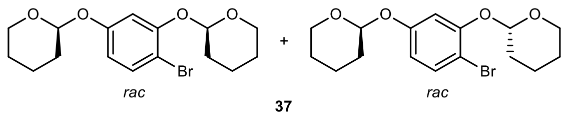

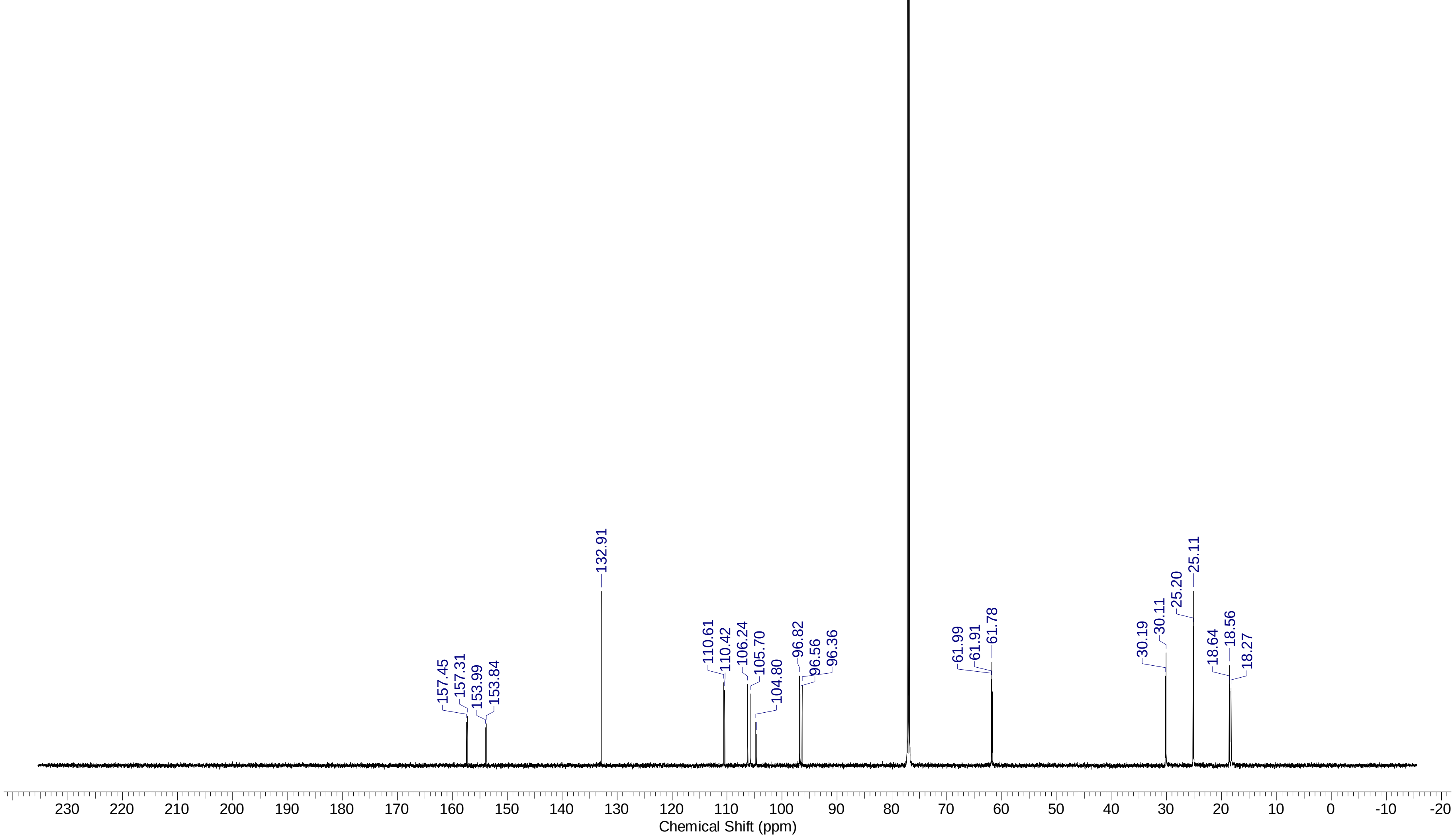

Supplementary Figure 54. ^{13}C NMR of **37**

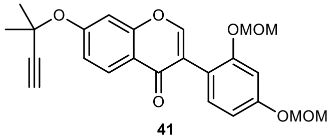

41

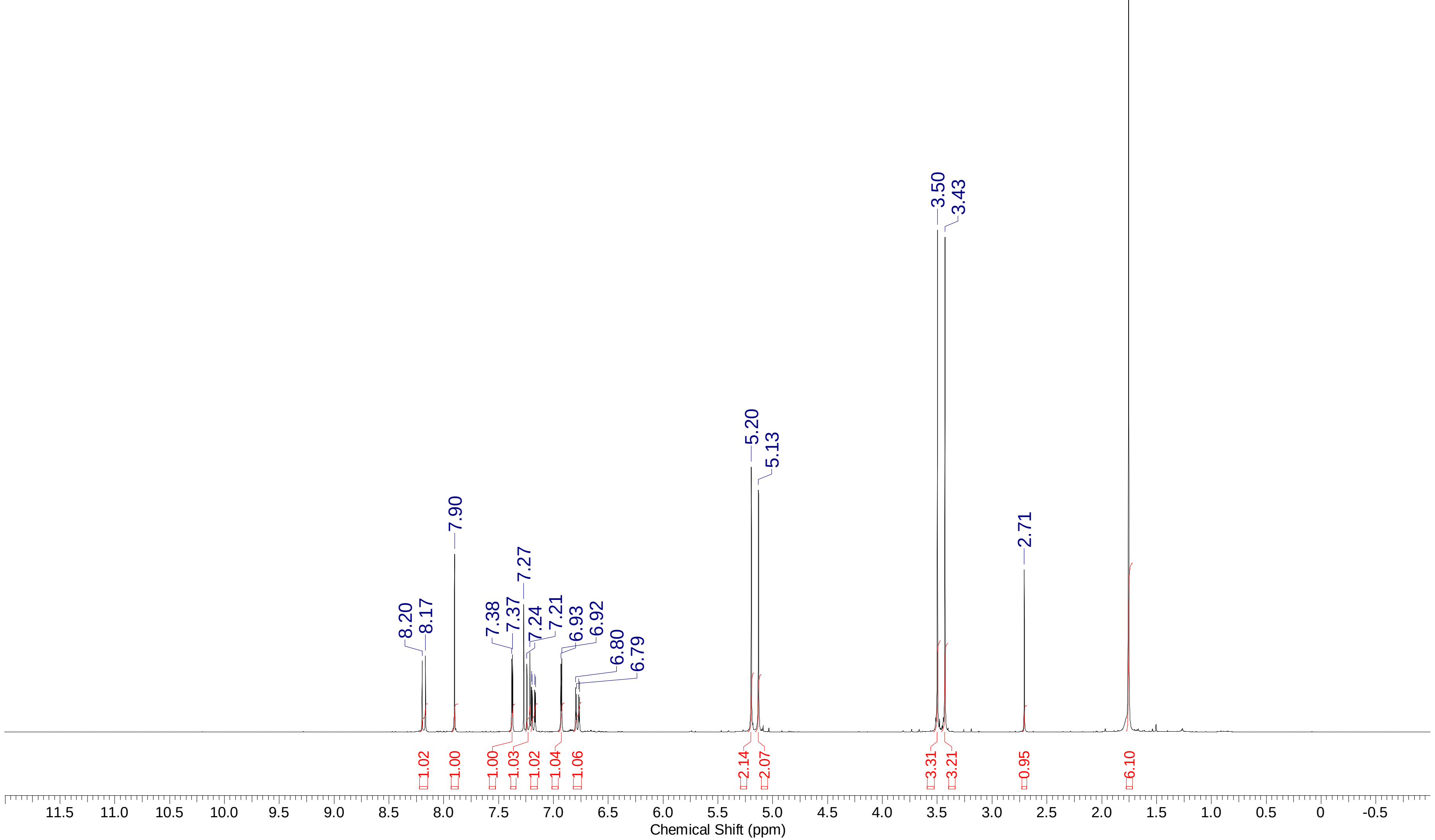

Supplementary Figure 55. ¹H NMR of **41**

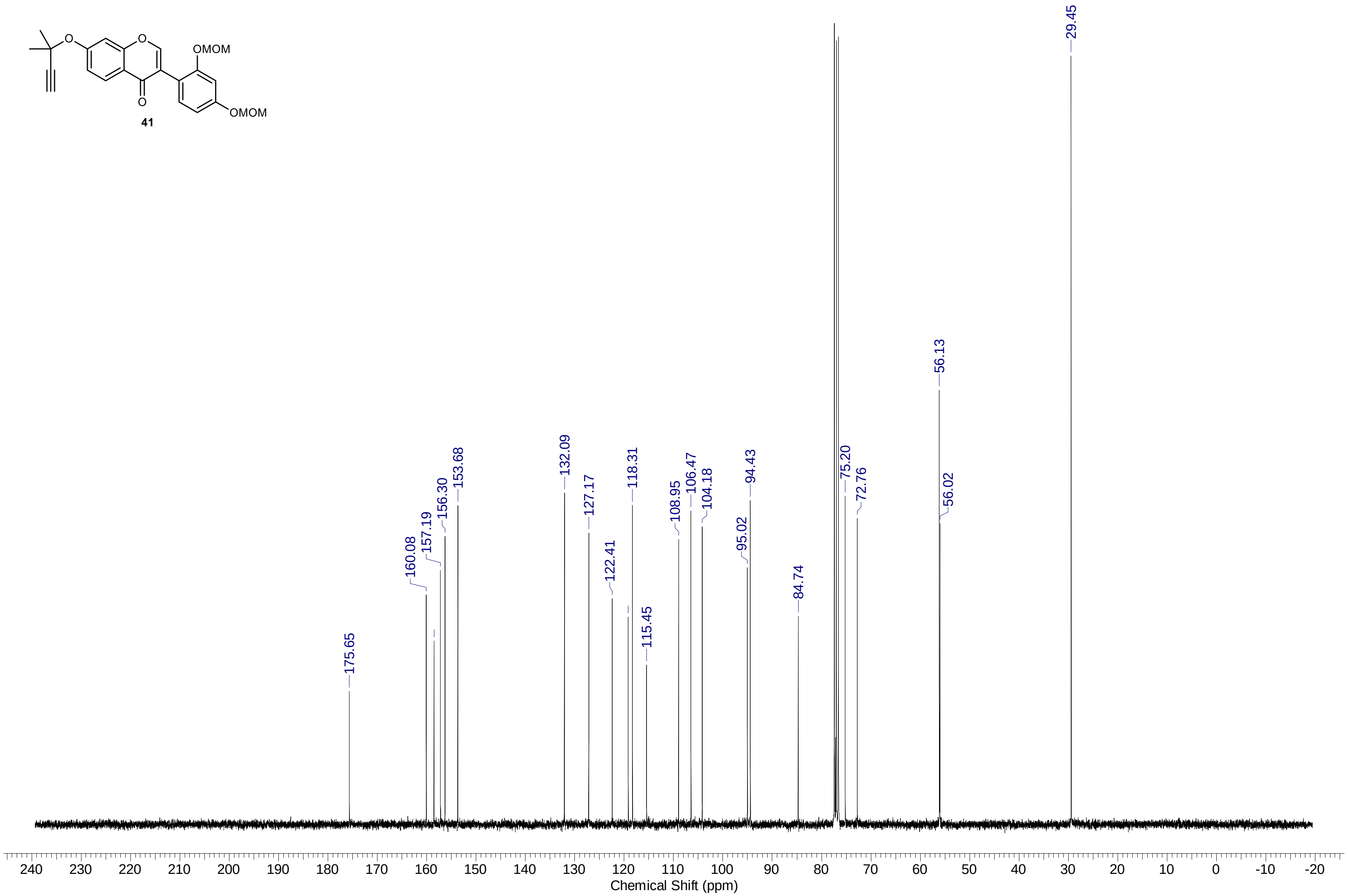

Supplementary Figure 56. ^{13}C NMR of **41**

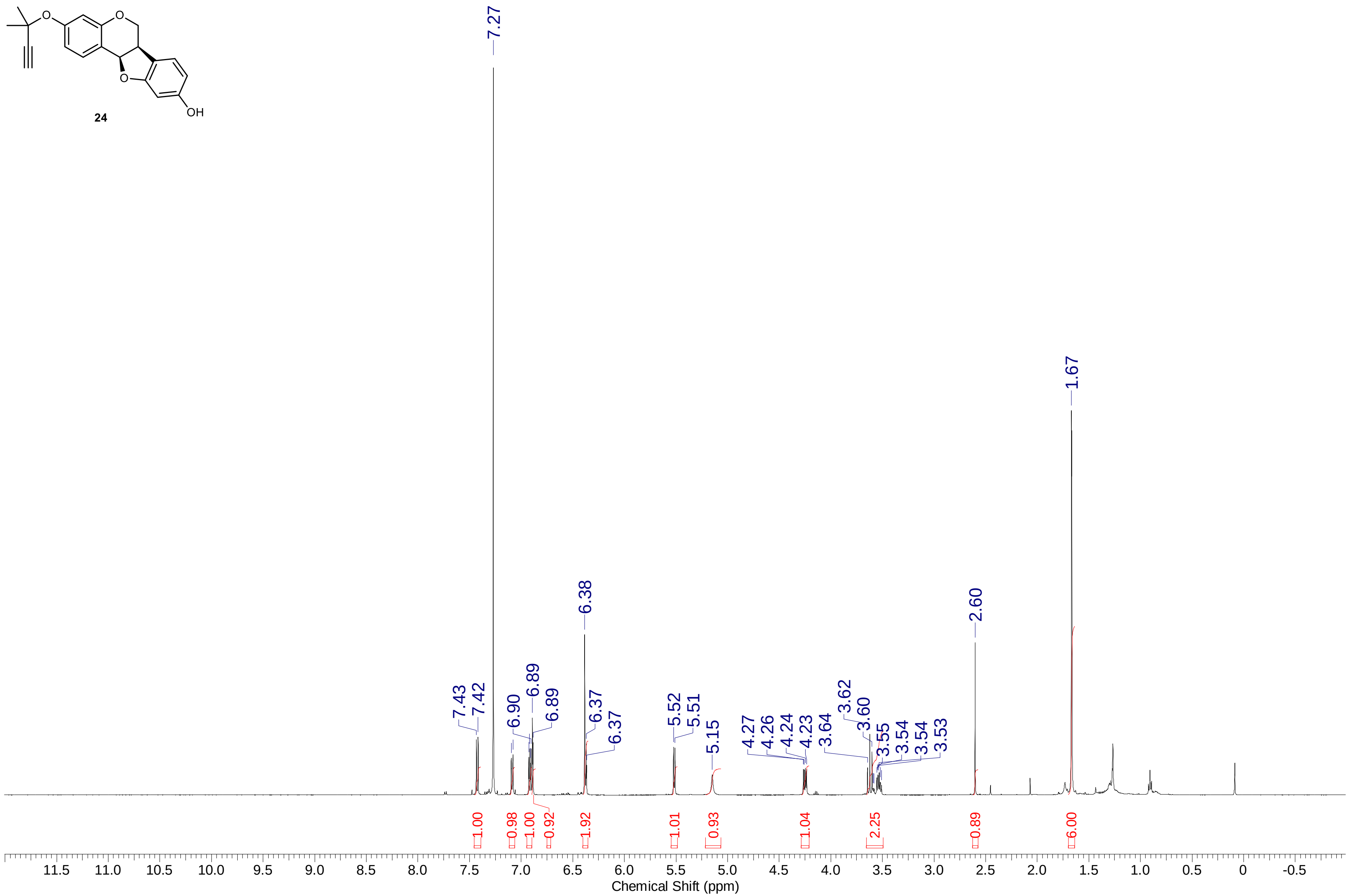

Supplementary Figure 57. ¹H NMR of **24**

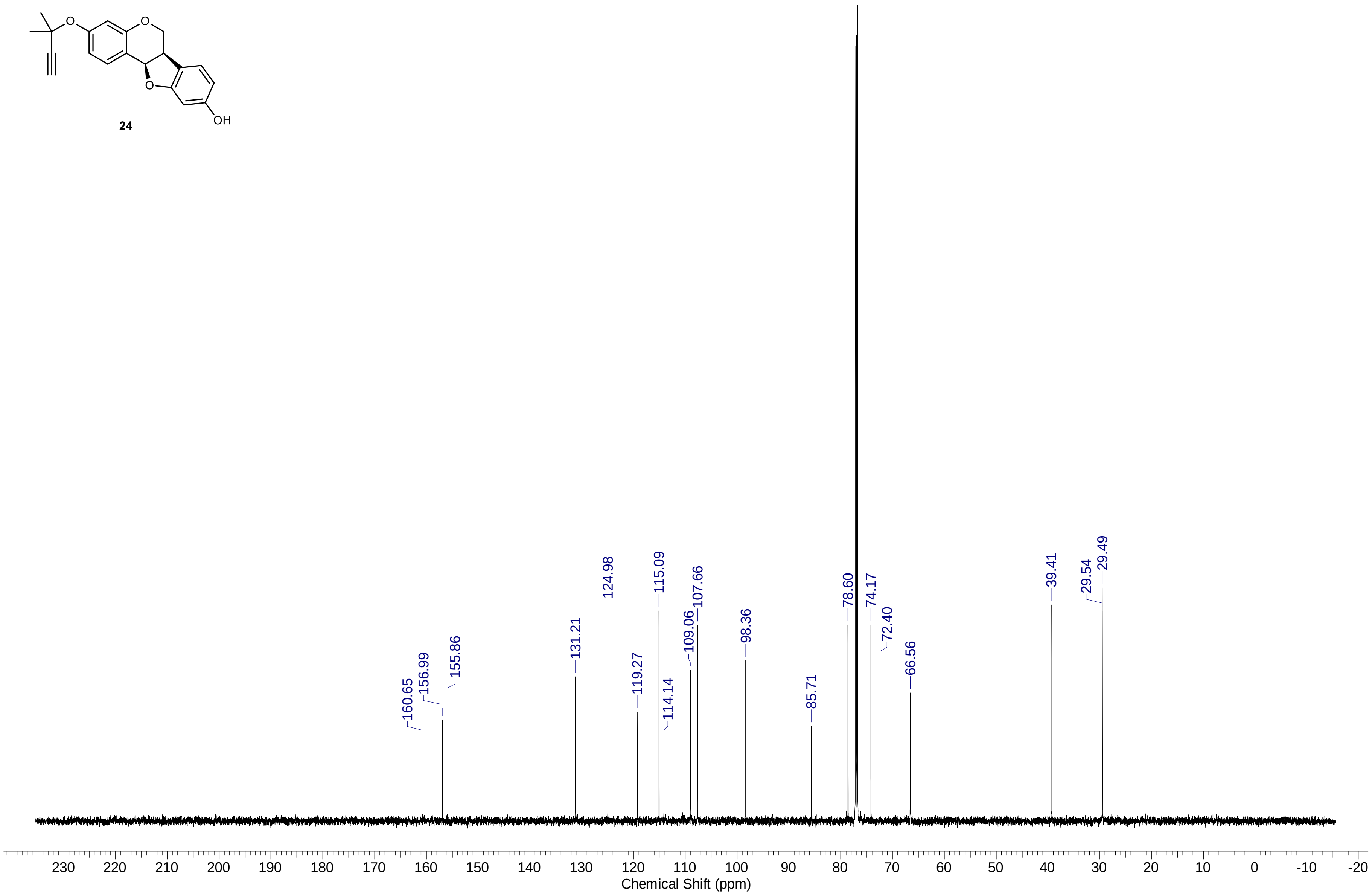

Supplementary Figure 58. ^{13}C NMR of **24**

Chiral HPLC trace of *rac*-**24** (bottom) and (*R*,*R*)-**24** (top, >99% *ee*); Method: Chiralpak® AD, hexane:isopropanol 80:20 *v/v*, 0.8 mL/min

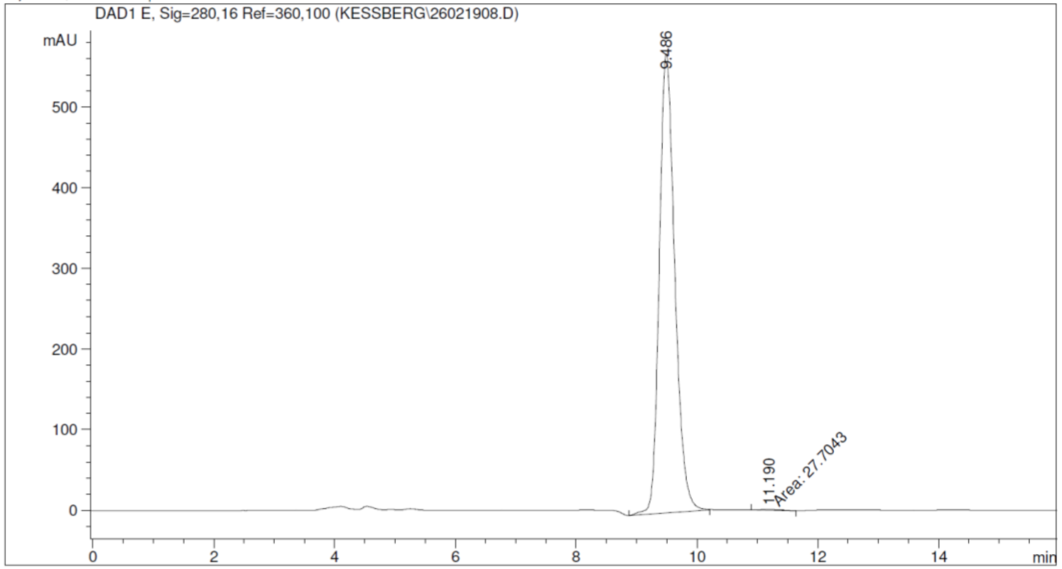

Peak #	RetTime [min]	Type	Width [min]	Area [mAU*s]	Height [mAU]	Area %
1	9.486	PB	0.2727	1.01896e4	569.52307	99.7289
2	11.190	MM T	0.4273	27.70429	1.08072	0.2711
Totals :				1.02173e4	570.60379	

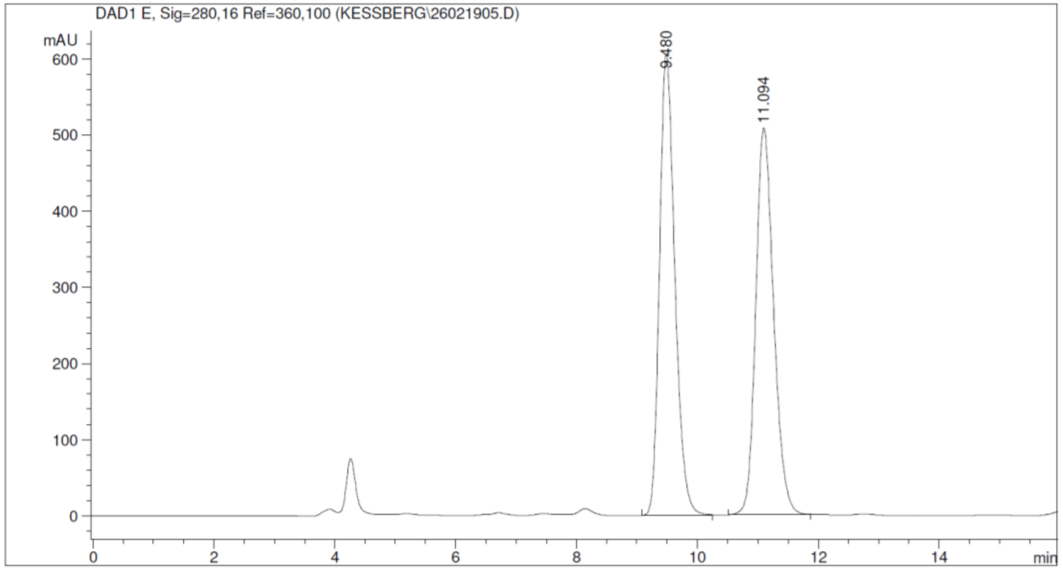

Supplementary Figure 59. Chiral HPLC trace of **24**

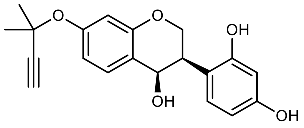

45

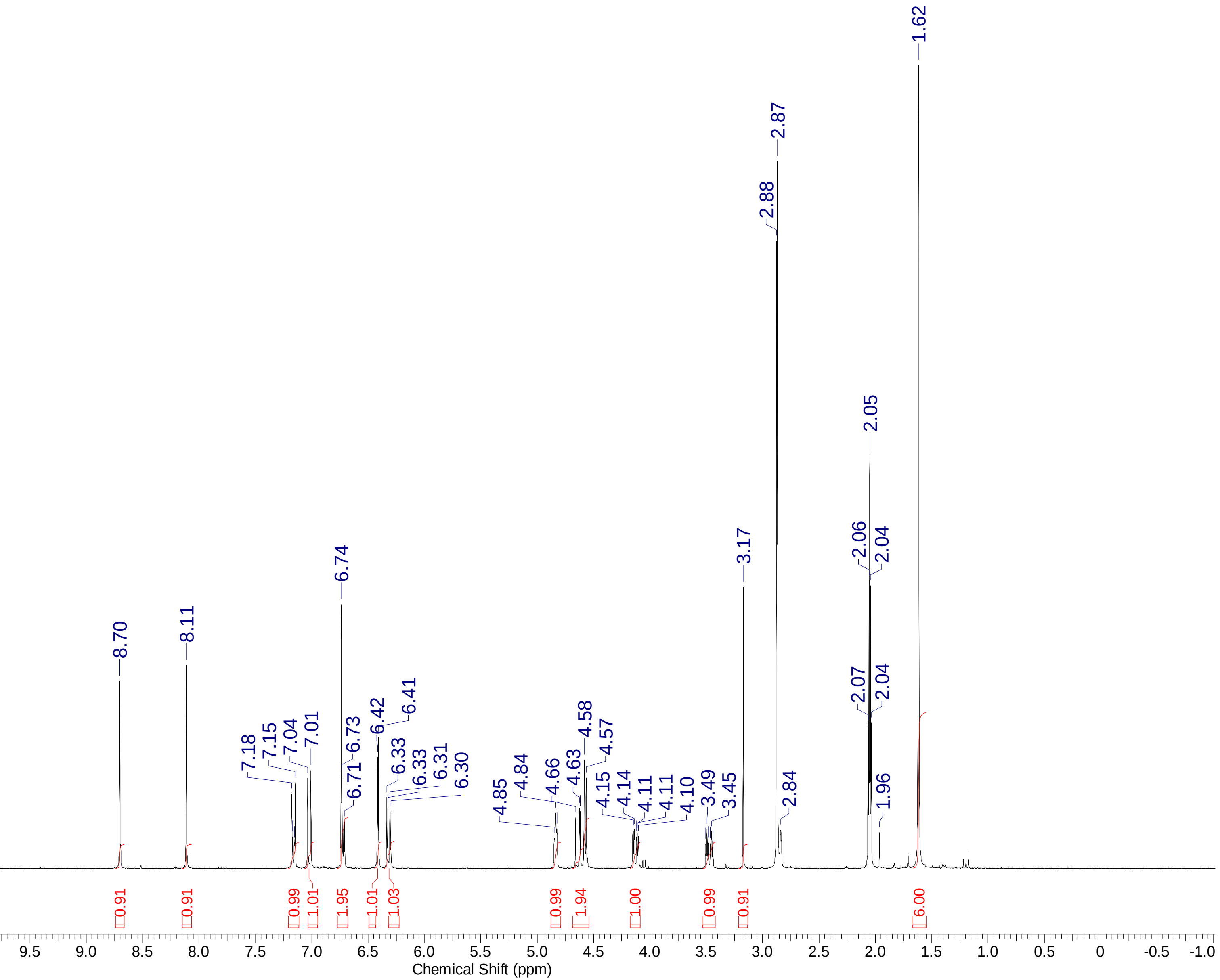

Supplementary Figure 60. ^{1}H NMR of **45**

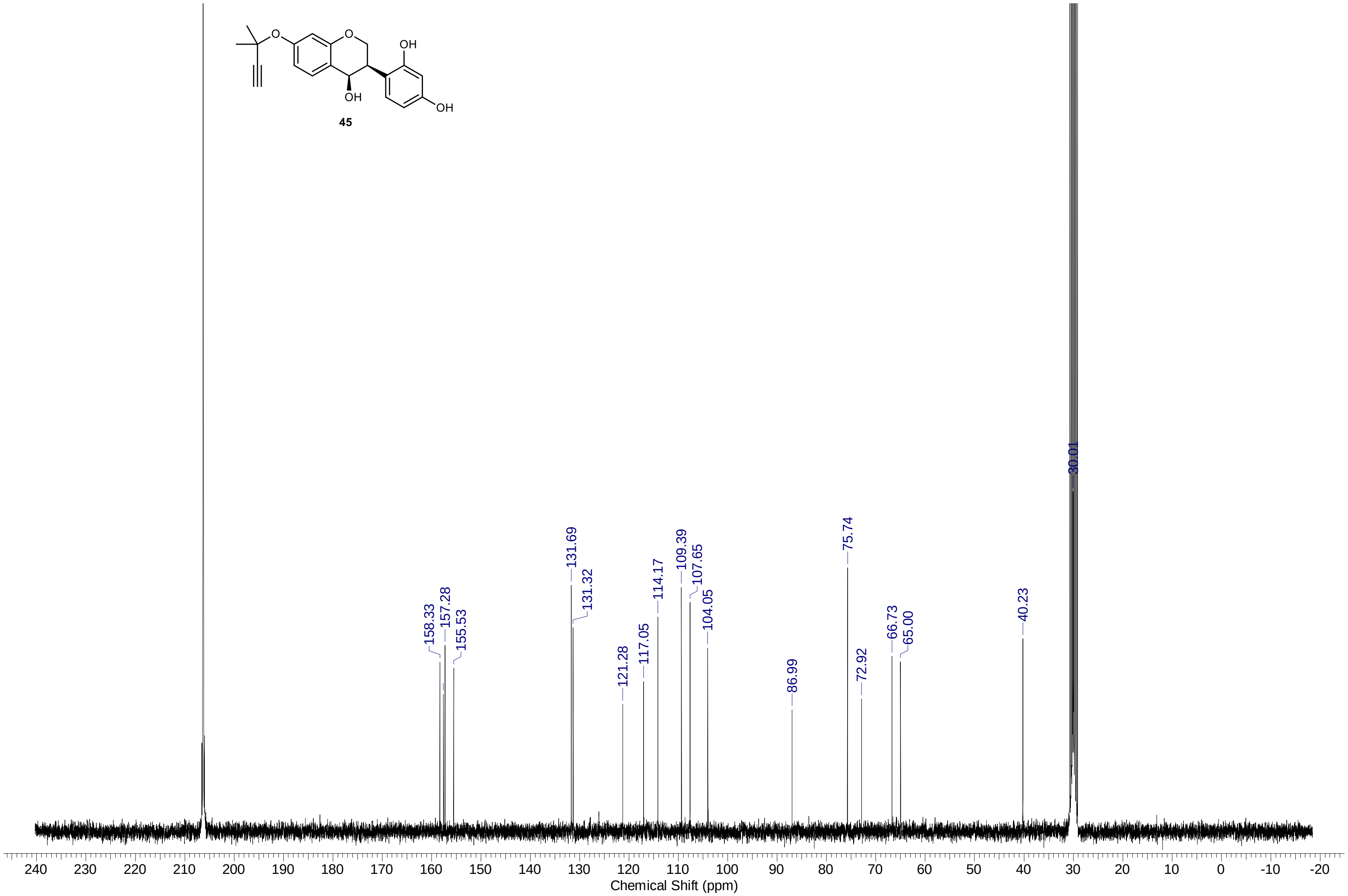

Supplementary Figure 61. ^{13}C NMR of **45**

Chiral HPLC trace of *rac*-**45** (bottom) and (*R*,*R*)-**45** (top, >99% *ee* with trace of impurity at 9.68 min); Method: Chiralpak® AD, hexane:isopropanol 70:30 *v/v*, 0.8 mL/min

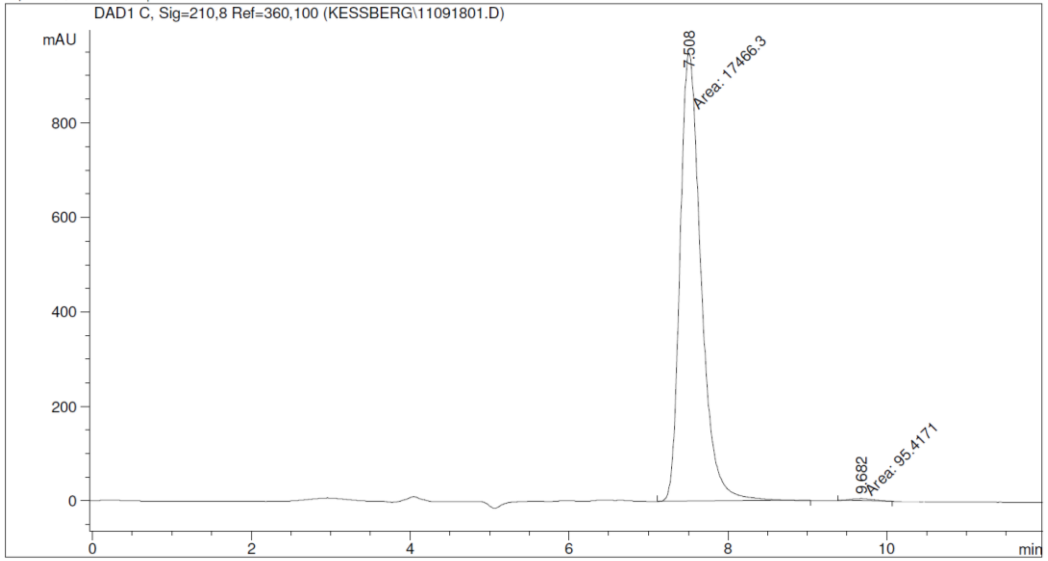

Peak #	RetTime [min]	Type	Width [min]	Area [mAU*s]	Height [mAU]	Area %
1	7.508	MM T	0.3069	1.74663e4	948.65497	99.4567
2	9.682	MM T	0.3249	95.41713	4.89483	0.5433
Totals :				1.75617e4	953.54980	

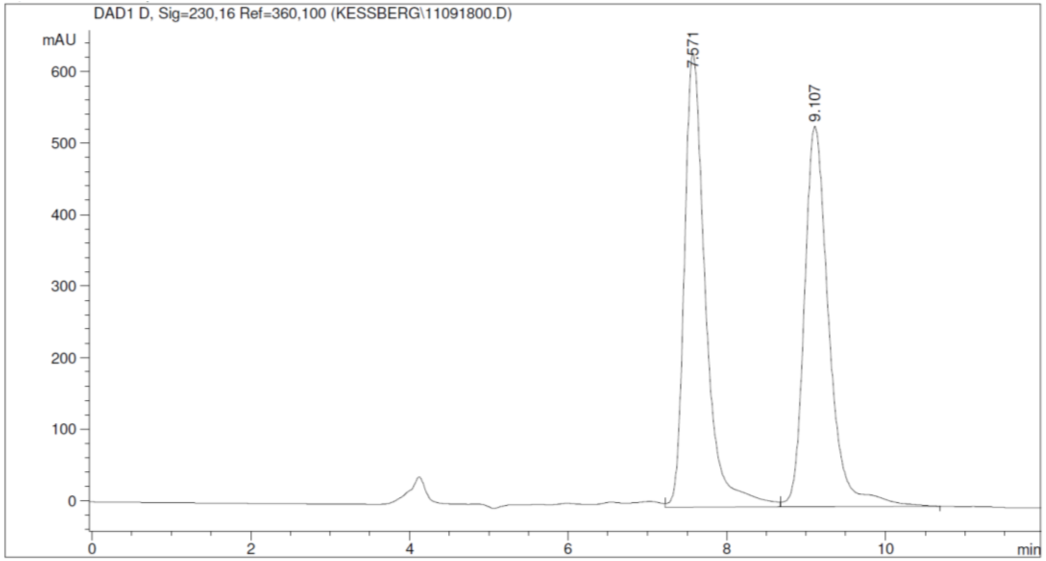

Supplementary Figure 62. Chiral HPLC trace of **45**

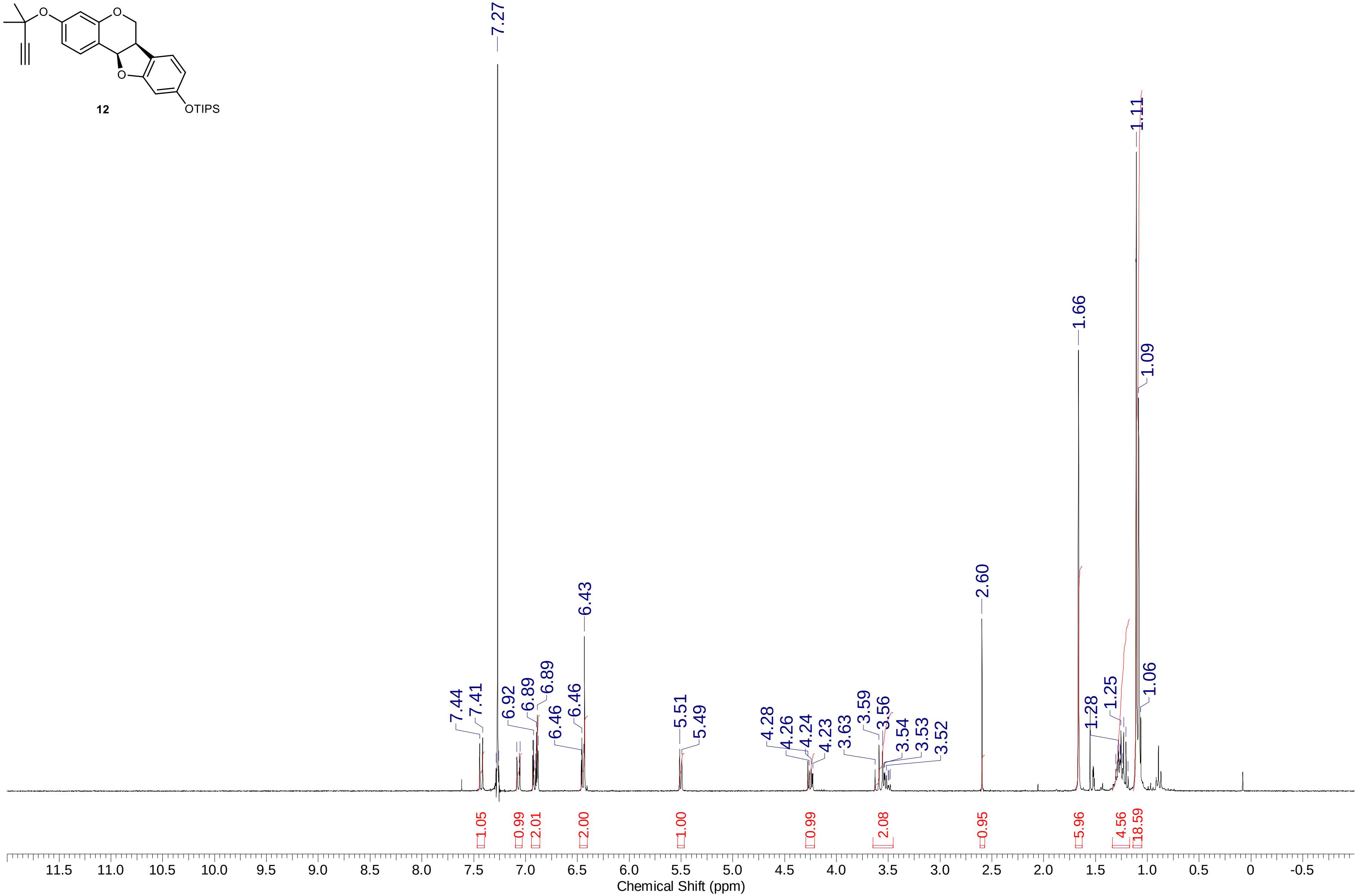

Supplementary Figure 63. ^{1}H NMR of **12**

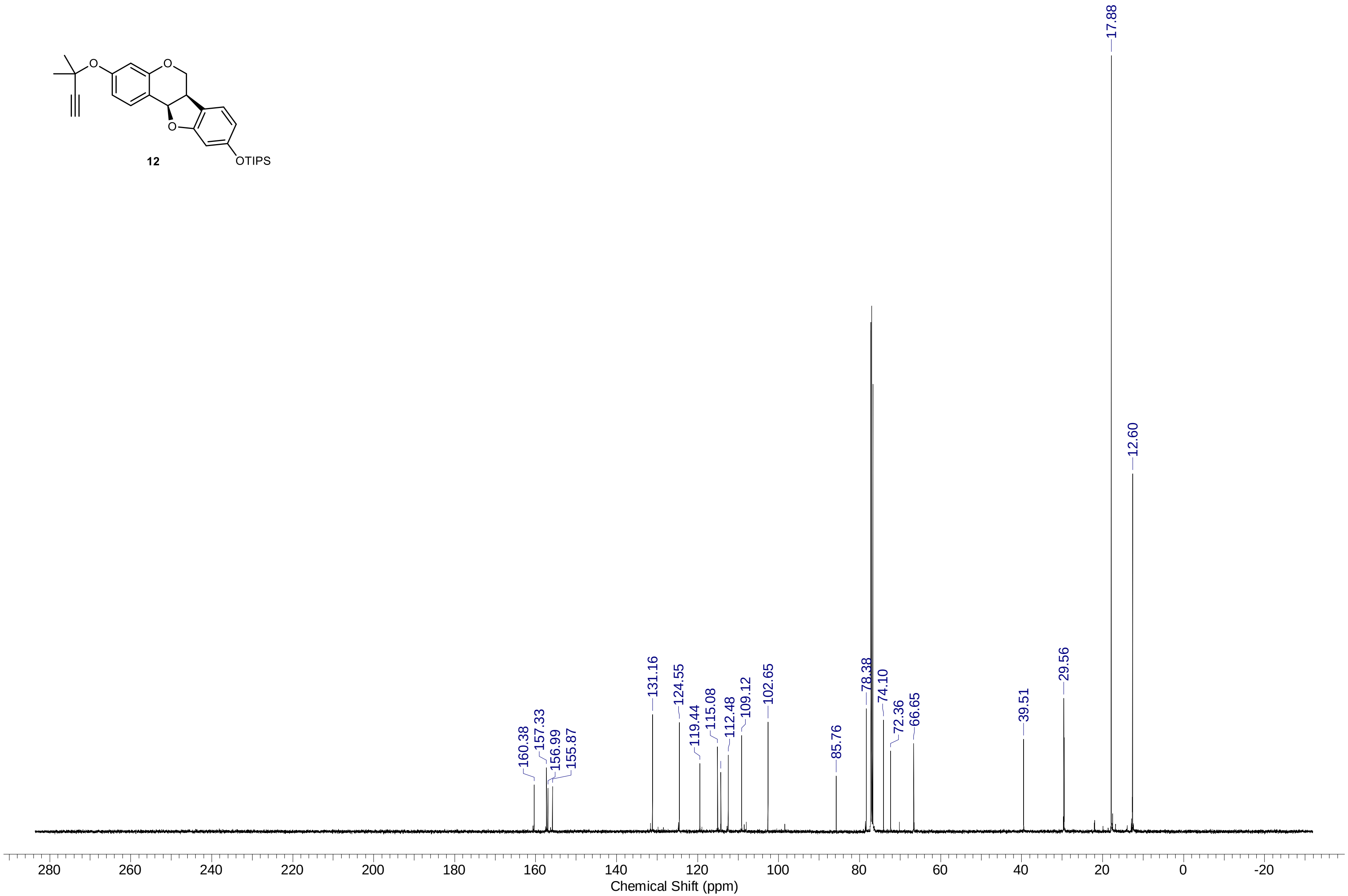

Supplementary Figure 64. ^{13}C NMR of **12**

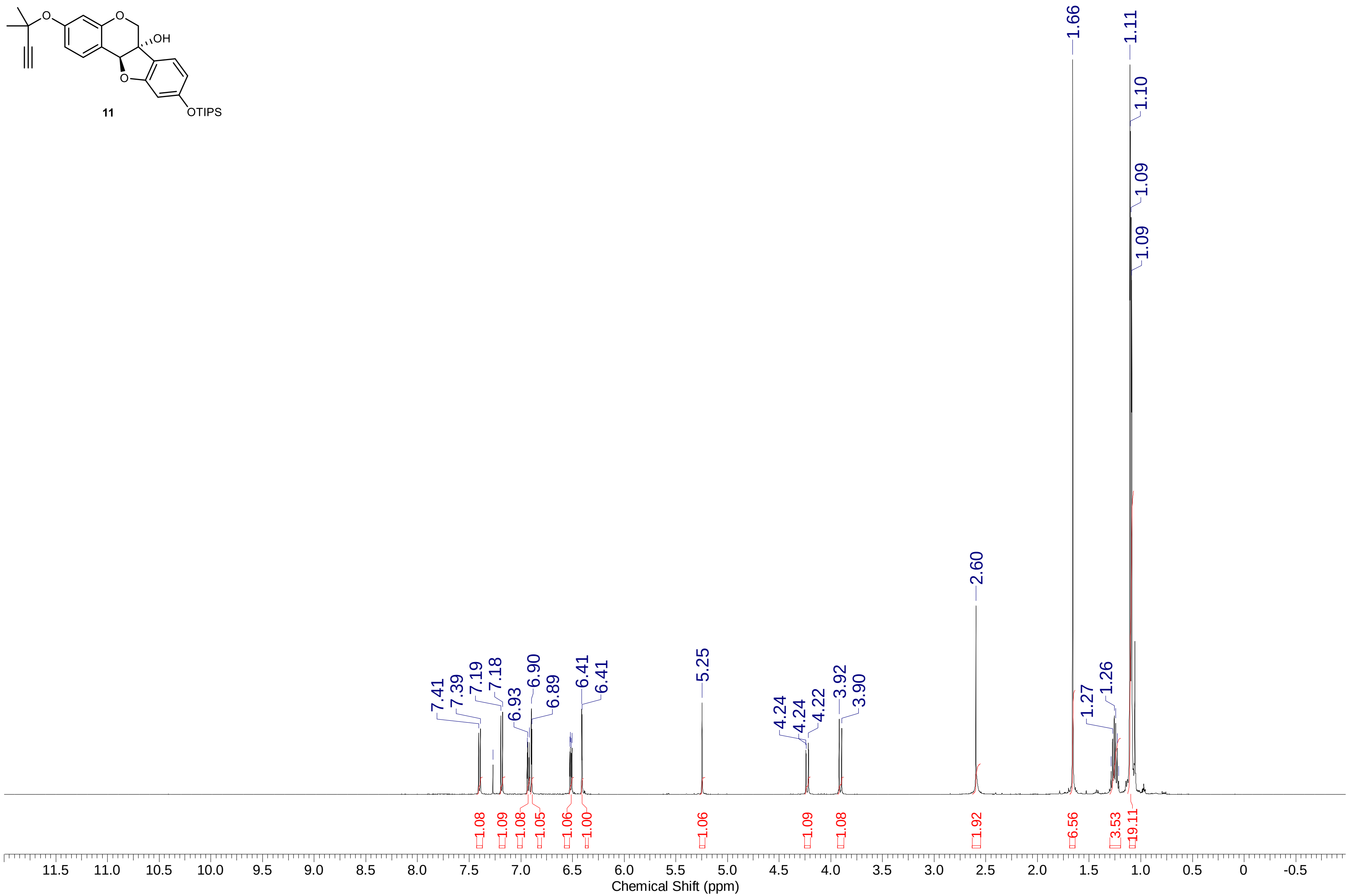

Supplementary Figure 65. ^{1}H NMR of **11**

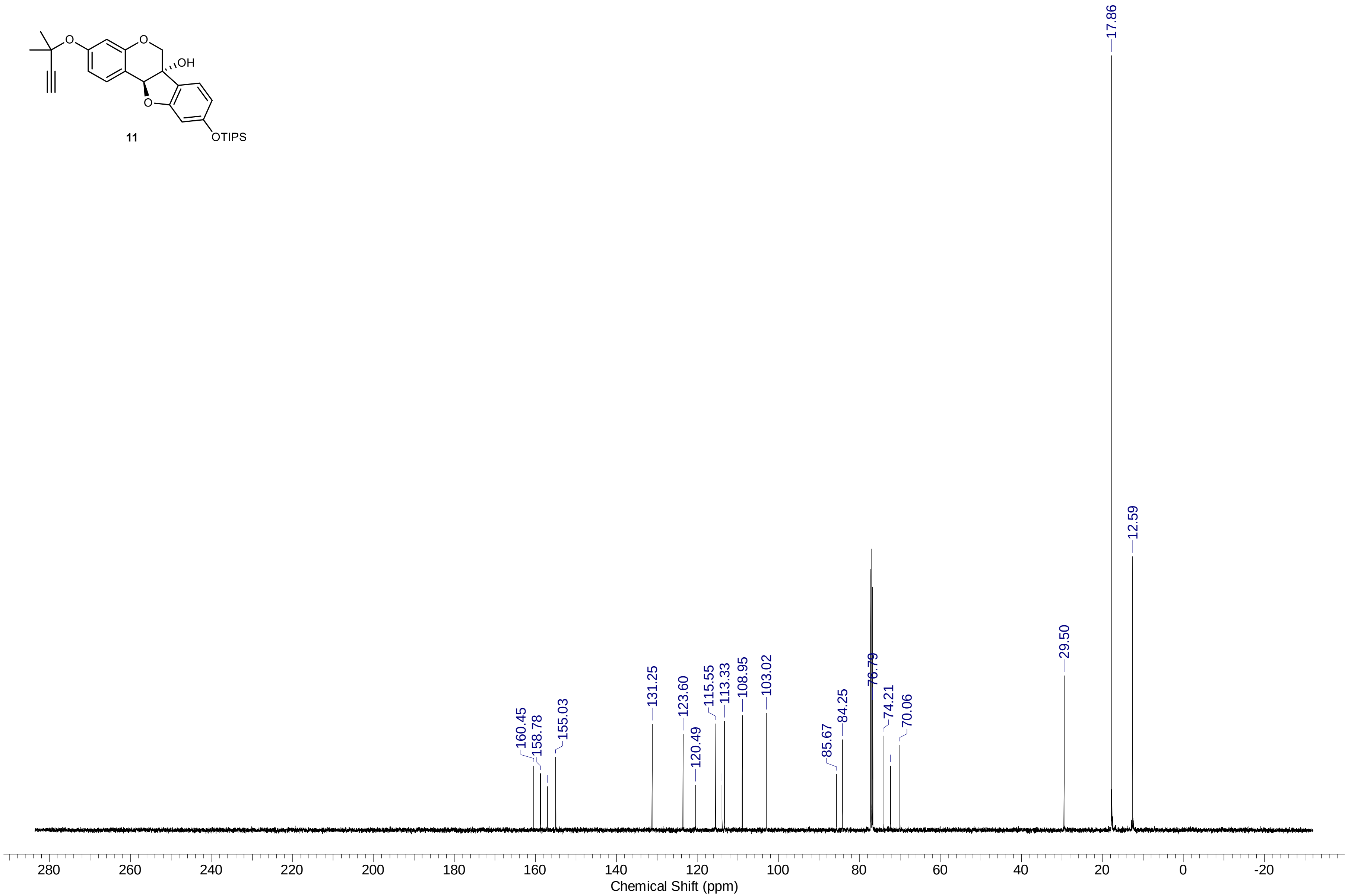

Supplementary Figure 66. ^{13}C NMR of **11**

Chiral HPLC trace of *rac*-**11** (bottom) and (*S*,*S*)-**11** (top, >99% *ee*); Method: Lux® Cellulose-1, hexane:isopropanol 90:10 *v/v*, 0.5 mL/min

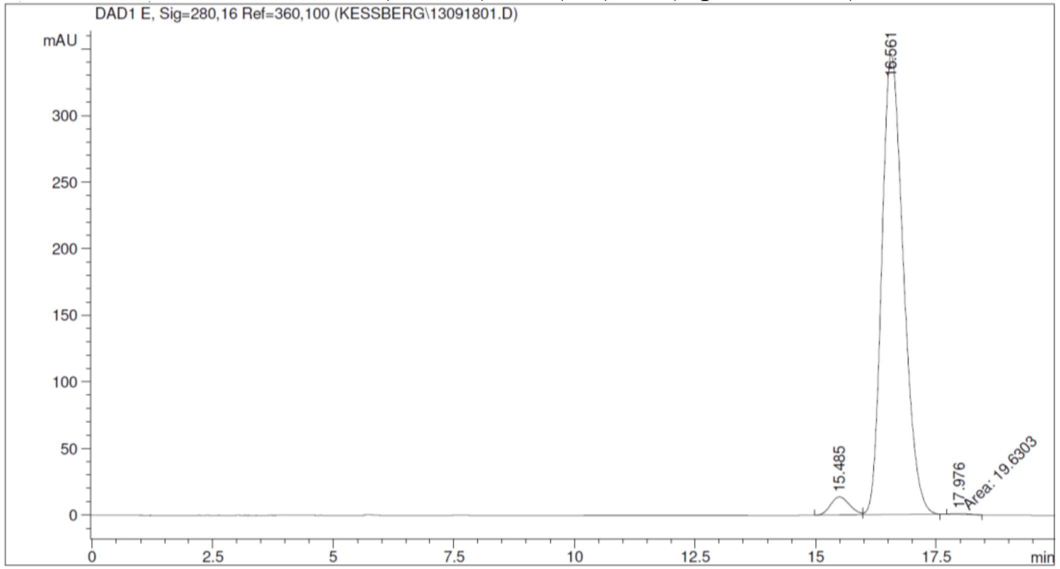

Peak #	RetTime [min]	Type	Width [min]	Area [mAU*s]	Height [mAU]	Area %
1	15.485	PV	0.4160	372.11862	13.65382	3.3359
2	16.561	VB	0.4830	1.07632e4	345.94046	96.4881
3	17.976	MM T	0.4003	19.63026	8.17358e-1	0.1760
Totals :				1.11550e4	360.41164	

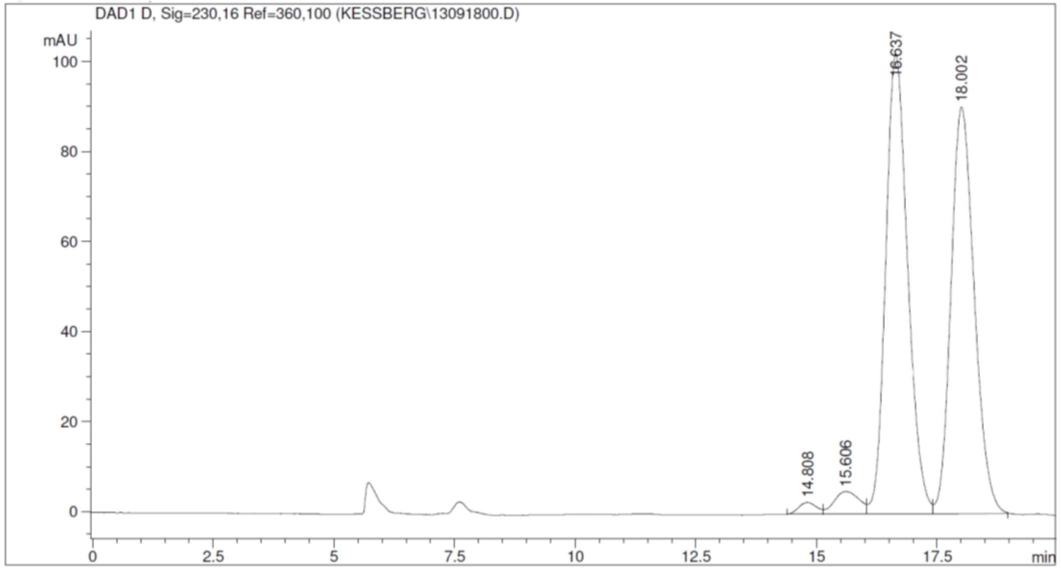

Supplementary Figure 67. Chiral HPLC trace of **11**

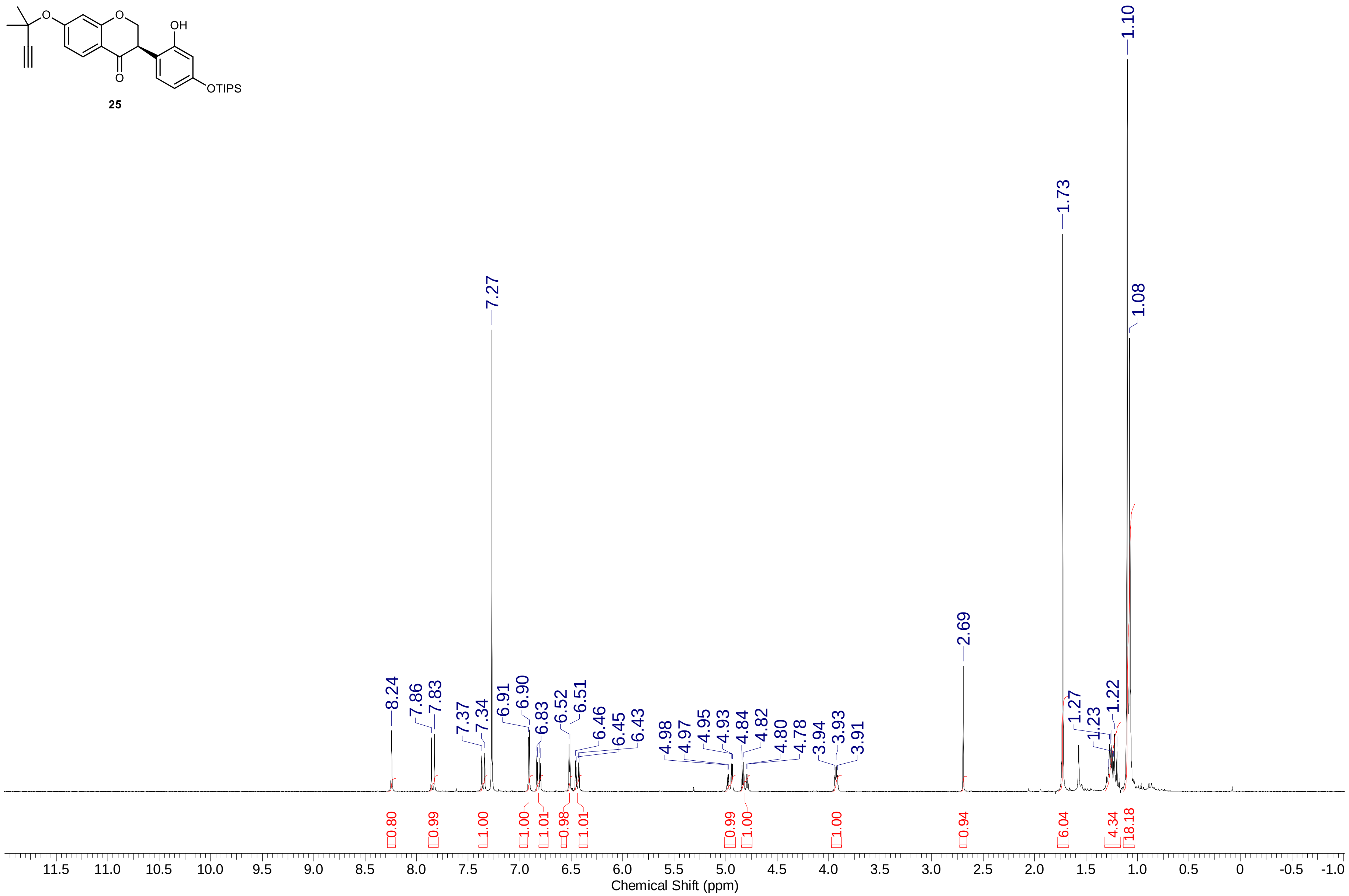

Supplementary Figure 68. ^{1}H NMR of **25**

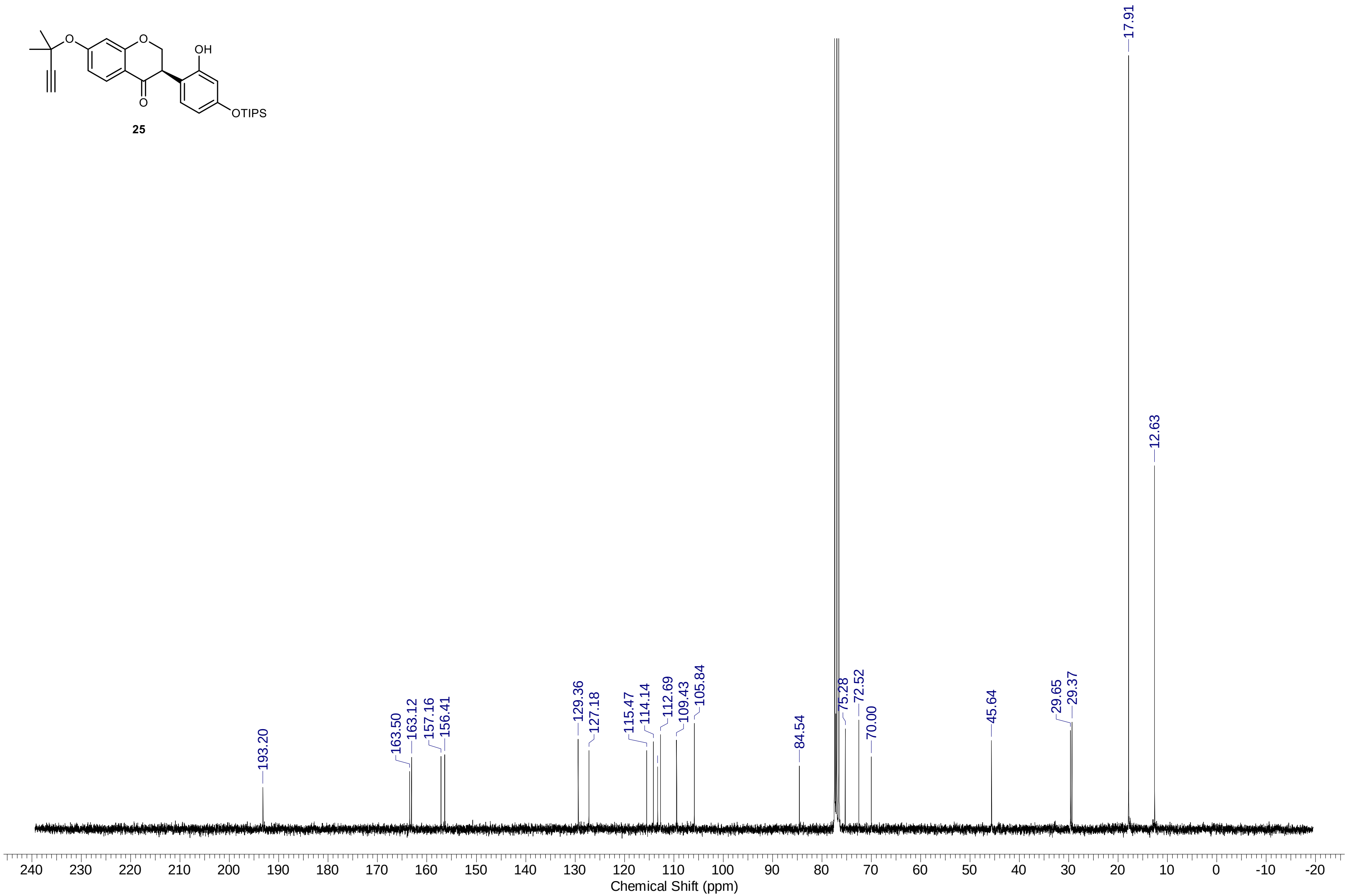

Supplementary Figure 69. ^{13}C NMR of **25**

Chiral HPLC trace of *rac*-**25** (bottom) and (*R*)-**25** (top, 92% *ee*); Method: Chiralpak® AD, hexane:isopropanol 90:10 *v/v*, 0.6 mL/min

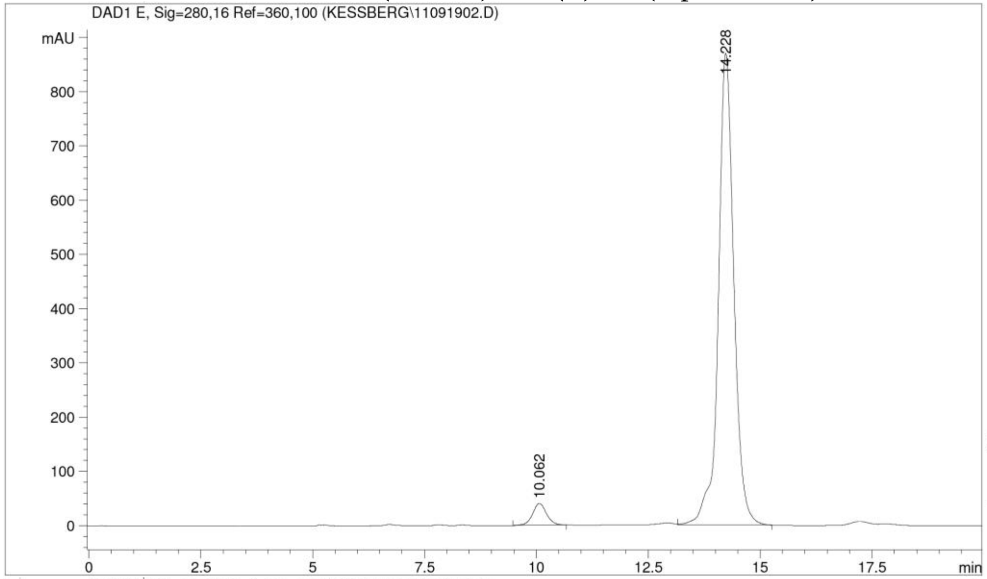

Peak #	RetTime [min]	Type	Width [min]	Area [mAU*s]	Height [mAU]	Area %
1	10.062	PB	0.3351	891.58655	40.45115	4.0709
2	14.228	VB	0.3658	2.10097e4	869.89215	95.9291

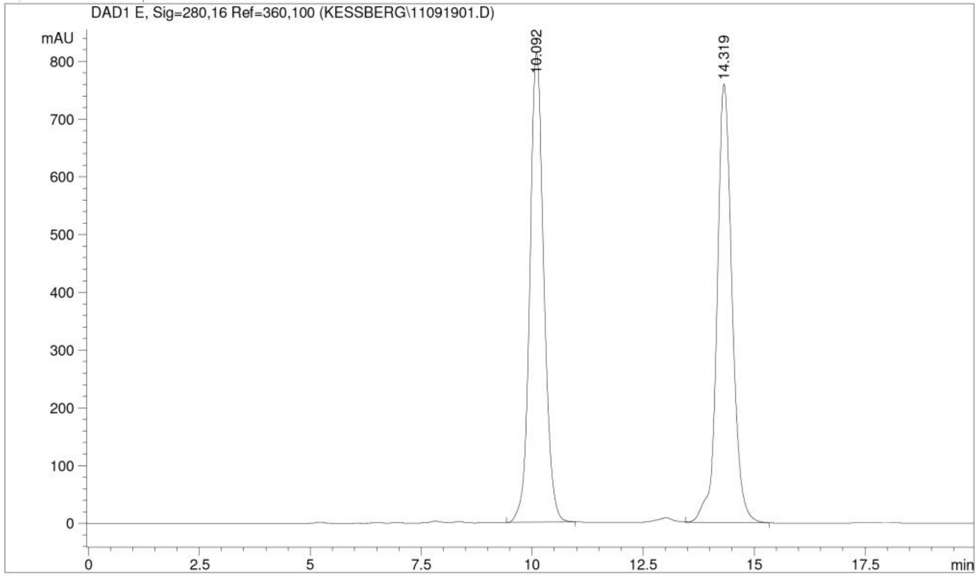

Supplementary Figure 70. Chiral HPLC trace of **25**

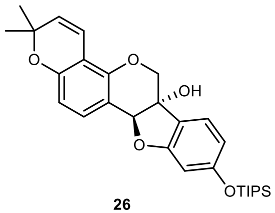

26

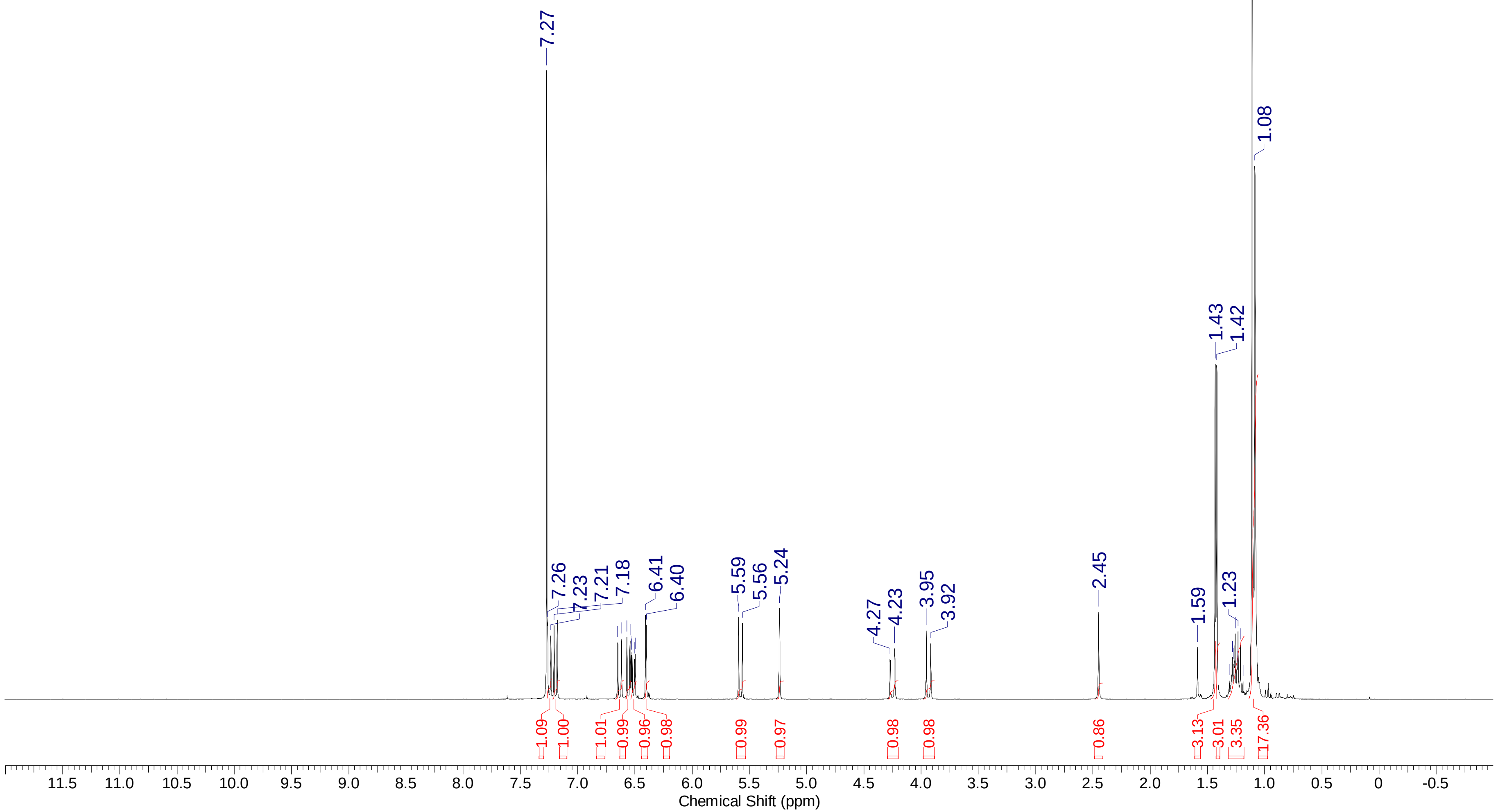

Supplementary Figure 71. ^{1}H NMR of **26**

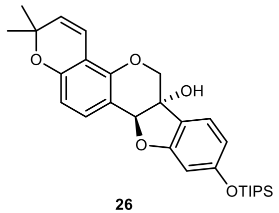

26

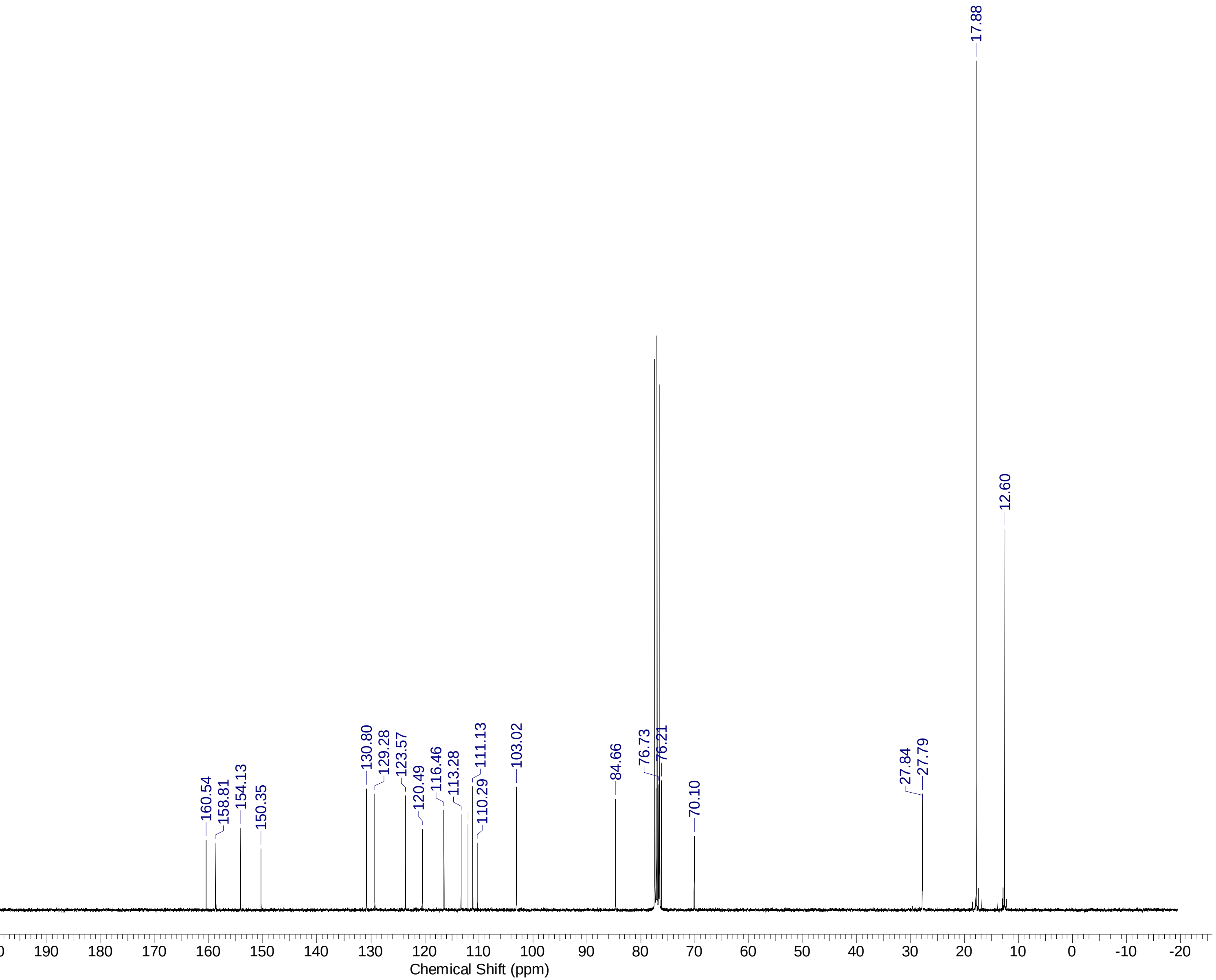

Supplementary Figure 72. ^{13}C NMR of **26**

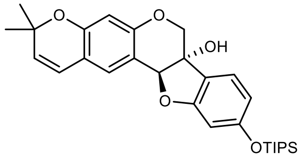

27

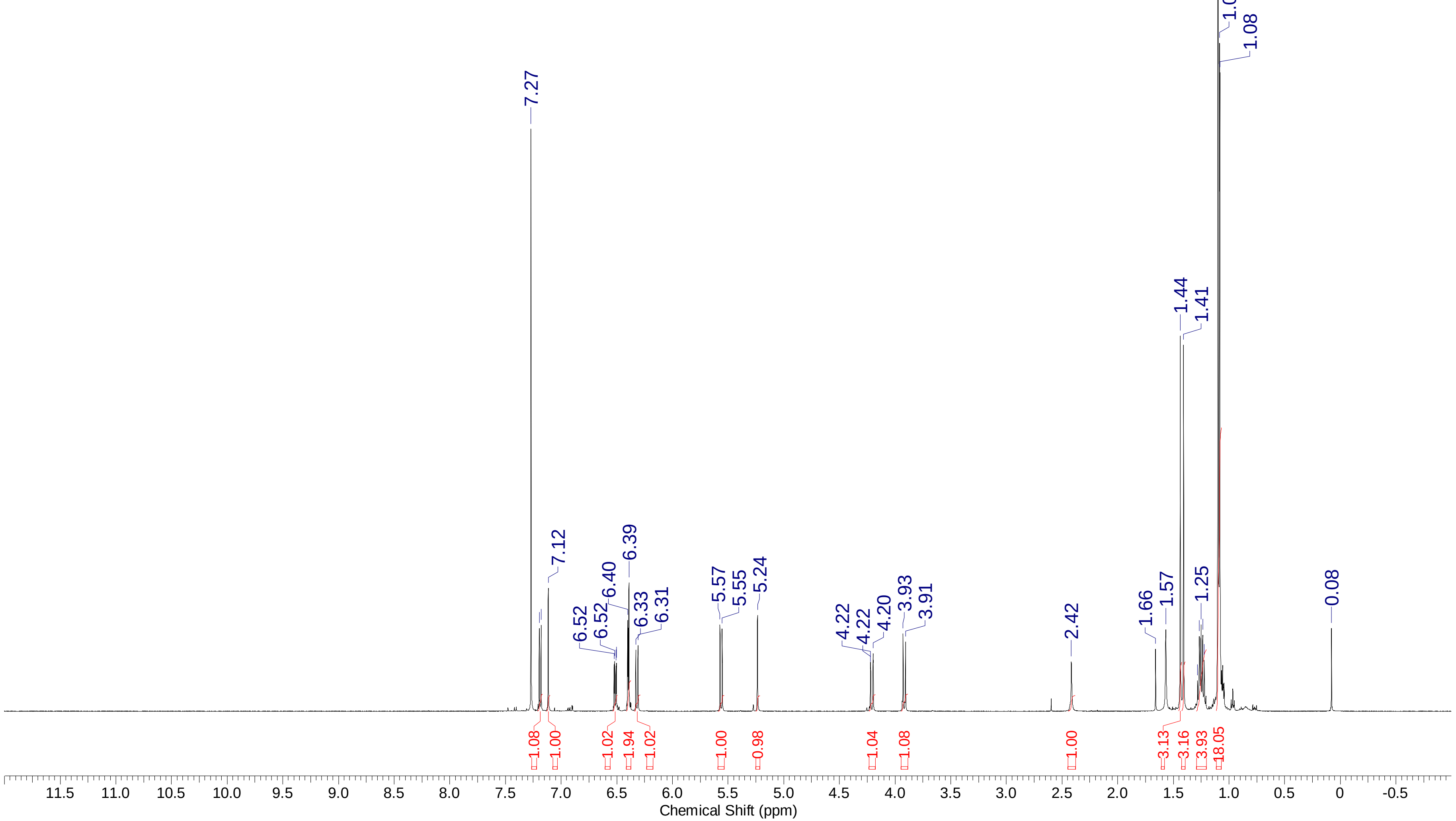

Supplementary Figure 73. ^{1}H NMR of **27**

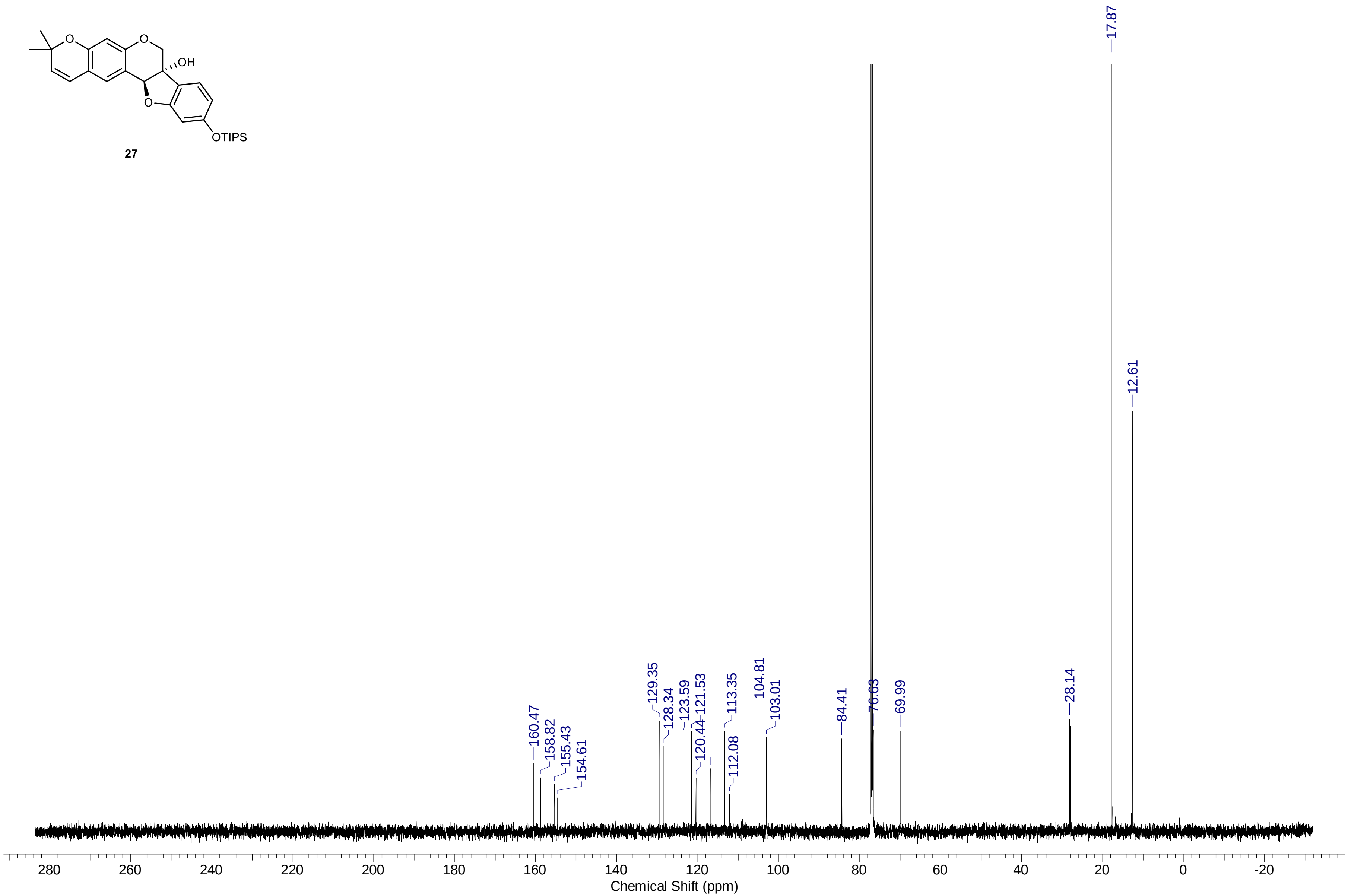

Supplementary Figure 74. ^{13}C NMR of **27**

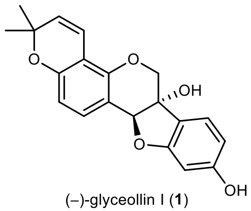

(–)-glyceollin I (**1**)

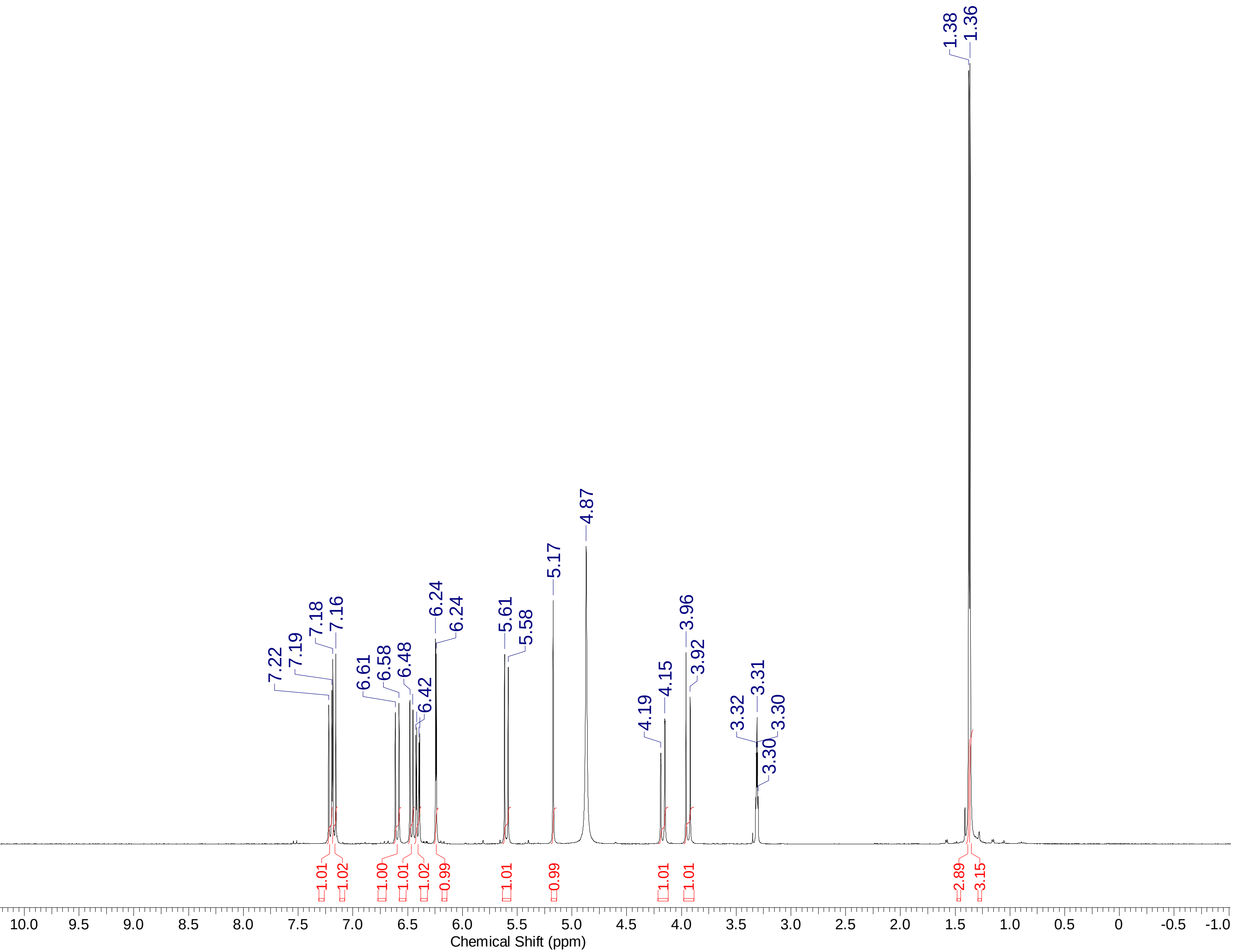

Supplementary Figure 75. ^{1}H NMR of **1**

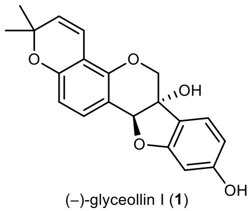

(–)-glyceollin I (**1**)

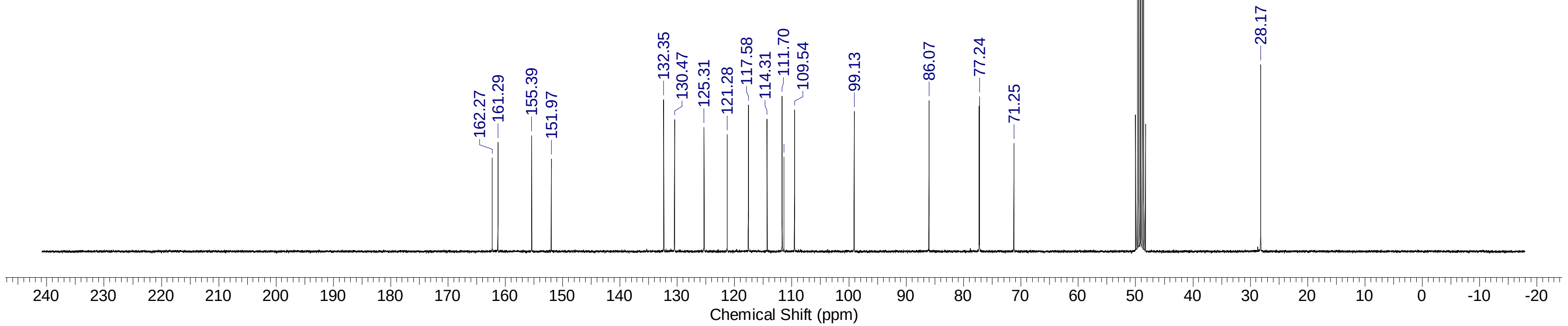

Supplementary Figure 76. ^{13}C NMR of **1**

Chiral HPLC trace of *rac*-glyceollin I (**1**, bottom) and (*S*,*S*)-glyceollin I (**1**, top, >99% *ee*); Method: Chiralpak® AD, hexane:isopropanol 80:20 *v/v*, 0.8 mL/min

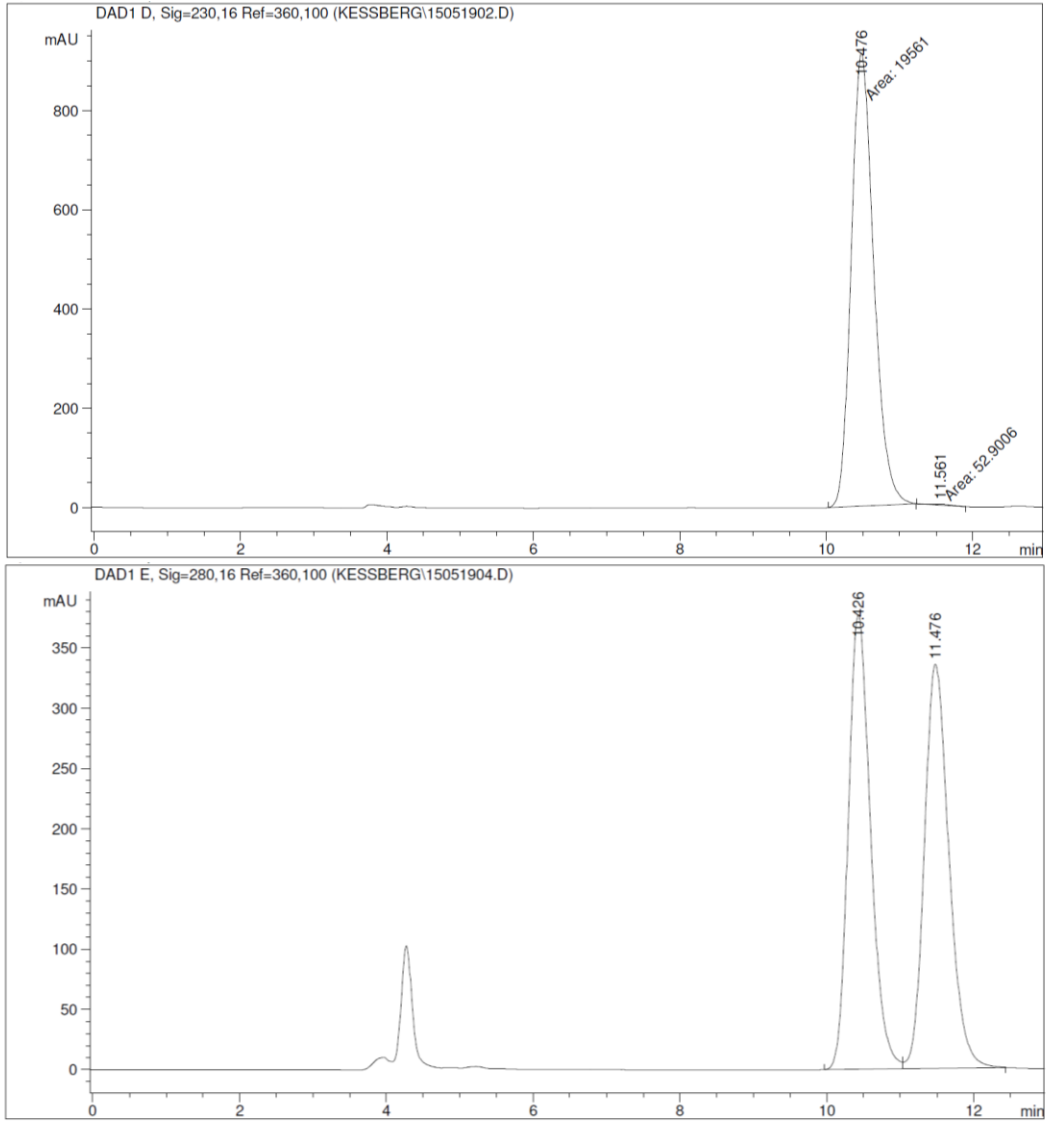

Peak #	RetTime [min]	Type	Width [min]	Area [mAU*s]	Height [mAU]	Area %
1	10.476	MM T	0.3569	1.95610e4	913.46295	99.7303
2	11.561	MM T	0.3528	52.90062	2.71504	0.2697
Totals :				1.96139e4	916.17799	

Supplementary Figure 77. Chiral HPLC trace of **1**

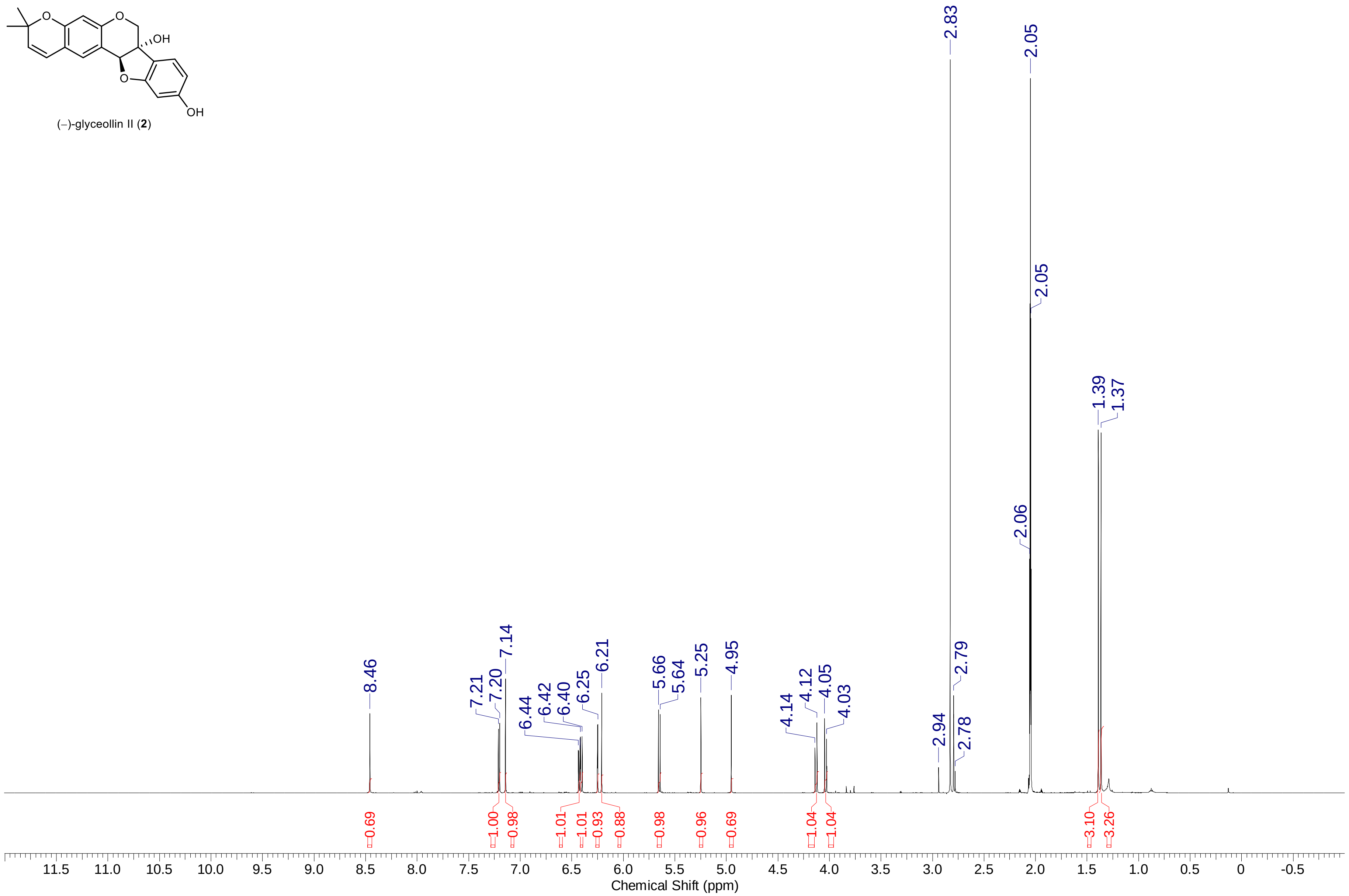

Supplementary Figure 78. ^{1}H NMR of **2**

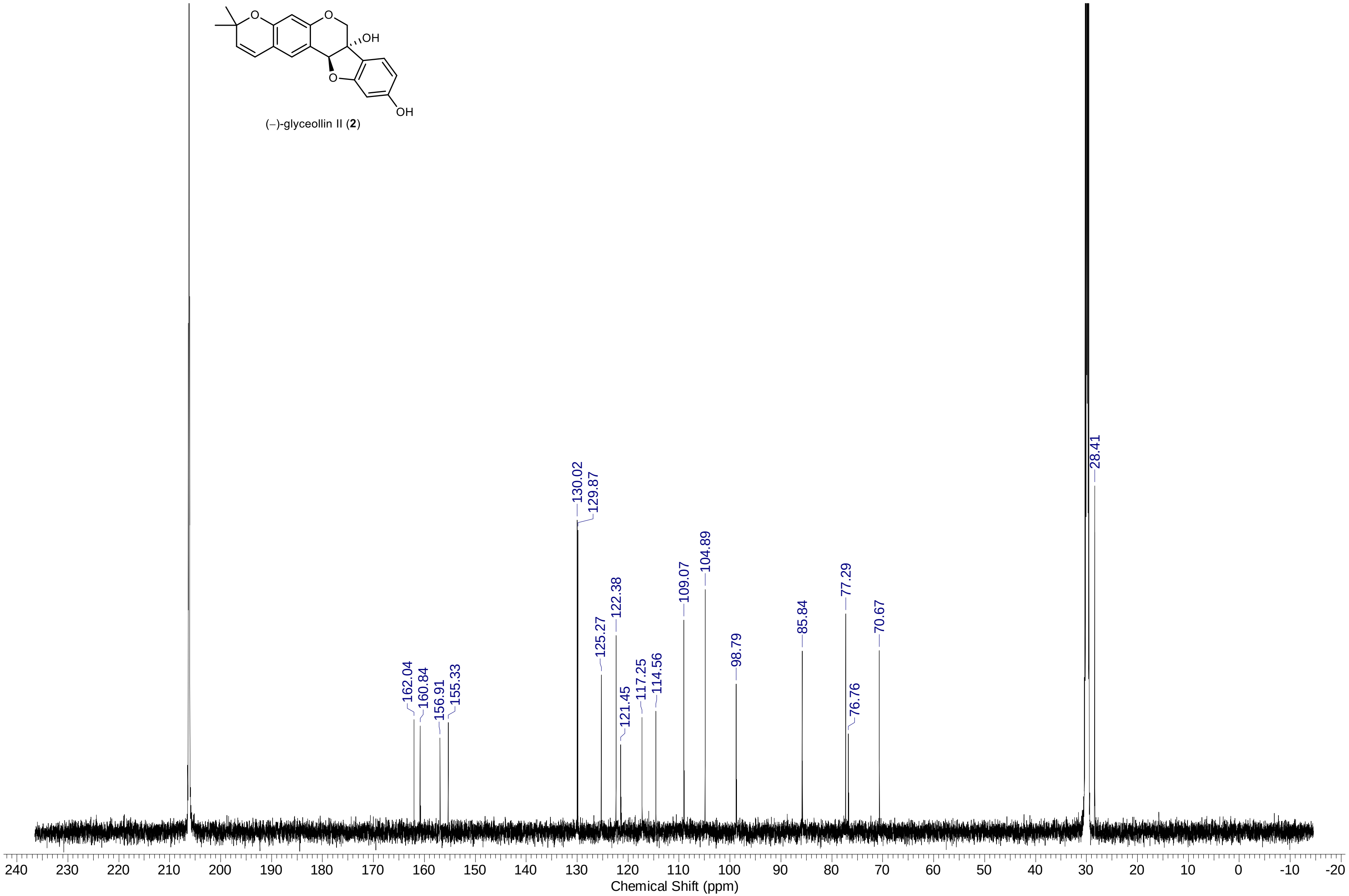

Supplementary Figure 79. ^{13}C NMR of **2**

Chiral HPLC trace of *rac*-glyceollin II (**2**, bottom) and (*S*,*S*)-glyceollin II (**2**, top, >99% *ee*); Method: Chiralpak® AD, hexane:isopropanol 80:20 *v/v*, 0.8 mL/min

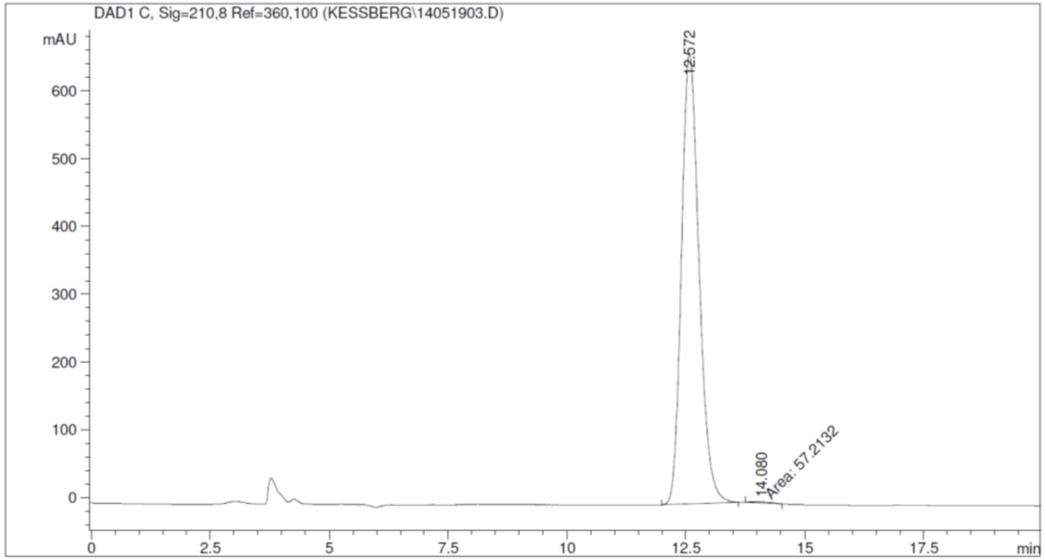

Peak #	RetTime [min]	Type	Width [min]	Area [mAU*s]	Height [mAU]	Area %
1	12.572	PB	0.3953	1.70694e4	665.28790	99.6659
2	14.080	MM T	0.5183	57.21320	1.83969	0.3341
Totals :				1.71267e4	667.12759	

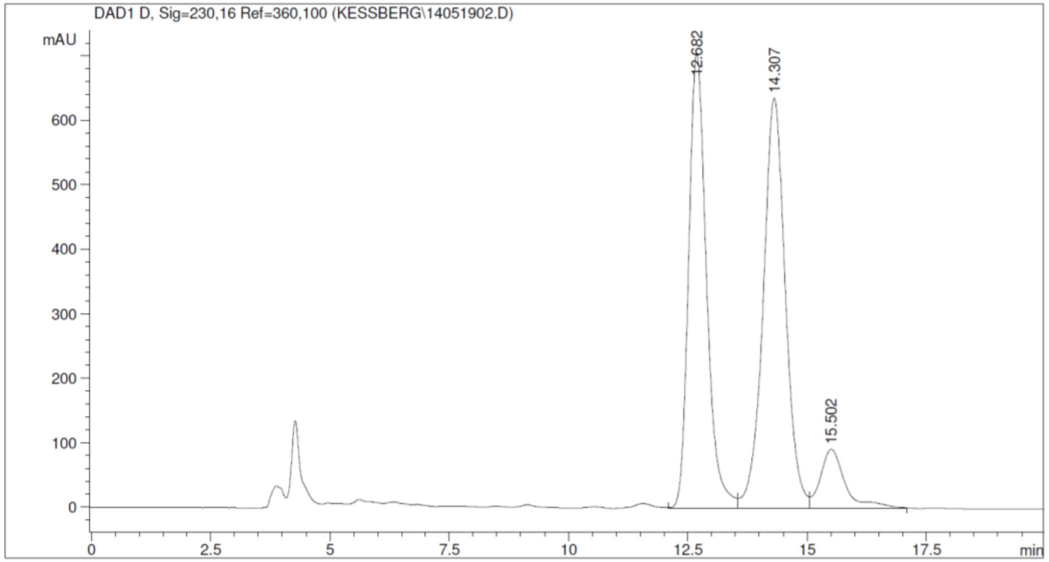

Supplementary Figure 80. Chiral HPLC trace of **2**

Supplementary References

1. Baier, S. Totalsynthesen der Hydroxypterocarpane (±)-Glyceollin I and II sowie einiger verwandter Vertreter. (PhD thesis Technische Universität Dresden, 2013).
2. Ciesielski, P. & Metz, P. A conceptually novel construction of the 6a-hydroxypterocarpan skeleton – Synthesis of (±)-variabilin. *Bioorg. Med. Chem.* **25**, 6175–6177 (2017).
3. Russell, G. B., Sutherland, O. R. W., Hutchins, R. F. N. & Christmas, P. E. Vestitol: A phytoalexin with insect feeding-deterrent activity. *J. Chem. Ecol.* **4**, 571–579 (1978).
4. Yahara, S. *et al.* Isoflavan and related compounds from dalbergia odorifera. I. *Chem. Pharm. Bull. (Tokyo)* **37**, 979–987 (1989).
5. Chang, L. C. *et al.* Activity-guided isolation of constituents of tephrosia purpurea with the potential to induce the phase II enzyme, quinone reductase. *J. Nat. Prod.* **60**, 869–873 (1997).
6. Bezuidenhoudt, B. C. B., Brandt, E. V. & Ferreira, D. Flavonoid analogues from pterocarpus species. *Phytochemistry* **26**, 531–535 (1987).
7. van Aardt, T. G., van Rensburg, H. & Ferreira, D. Synthesis of isoflavonoids. Enantiopure *cis*- and *trans*-6a-hydroxypterocarpans and a racemic *trans*-pterocarpan. *Tetrahedron* **57**, 7113–7126 (2001).
8. Visser, F. R. & Lane, G. A. Selectivity of the hydrogenation of 2’,4’,7-tribenzyloxyisoflavone. *Aust. J. Chem.* **40**, 1705–1711 (1987).
9. Basha, G. M. *et al.* A mild and efficient protocol to synthesize chromones, isoflavones, and homoisoflavones using the complex 2,4,6-trichloro-1,3,5-triazine/dimethylformamide. *Can. J. Chem.* **91**, 763–768 (2013).
10. Aghoramurthy, K., Kukla, A. S. & Seshadri, T. R. Synthesis of 2’-hydroxy-4,7’-dimethoxyisoflavone. *J. Indian Chem. Soc.* **38**, 914–918 (1961).

11. Gammill, R. B. A new and efficient synthesis of 3-halogenated 4*H*-1-benzopyran-4-ones. *Synthesis* **1979**, 901–903 (1979).

12. Foster, A. B. *et al.* Hydroxy derivatives of tamoxifen. *J. Med. Chem.* **28**, 1491–1497 (1985).

13. Dandepally, S. R. & Williams, A. L. Liebeskind–Srogl cross coupling mediated synthesis of verbenachalcone. *Tetrahedron Lett.* **51**, 5753–5756 (2010).

14. Luniwal, A. *et al.* Multigram synthesis of glyceollin I. *Org. Process Res. Dev.* **15**, 1149–1162 (2011).

15. Lyne, R. L., Mulheirn, L. J. & Leworthy, D. P. New pterocarpinoid phytoalexins of soybean. *J. Chem. Soc. Chem. Commun.* 497–498 (1976).
